# Unified approach to prenylated indole alkaloids: total syntheses of (–)-17-hydroxy-citrinalin B, (+)-stephacidin A, and (+)-notoamide I†
†Electronic supplementary information (ESI) available. CCDC 1400755 and 1400756. For ESI and crystallographic data in CIF or other electronic format see DOI: 10.1039/c5sc01977j


**DOI:** 10.1039/c5sc01977j

**Published:** 2015-06-18

**Authors:** Eduardo V. Mercado-Marin, Richmond Sarpong

**Affiliations:** a Department of Chemistry , University of California–Berkeley , Berkeley , CA 94720 , USA . Email: rsarpong@berkeley.edu

## Abstract

A unified strategy for the synthesis of congeners of the prenylated indole alkaloids is presented. This strategy has yielded the first synthesis of the natural product (–)-17-hydroxy-citrinalin B as well as syntheses of (+)-stephacidin A and (+)-notoamide I. An enolate addition to an *in situ* generated isocyanate was utilized in forging a key bicyclo[2.2.2]diazaoctane moiety, and in this way connected the two structural classes of the prenylated indole alkaloids through synthesis.

## Abstract 2

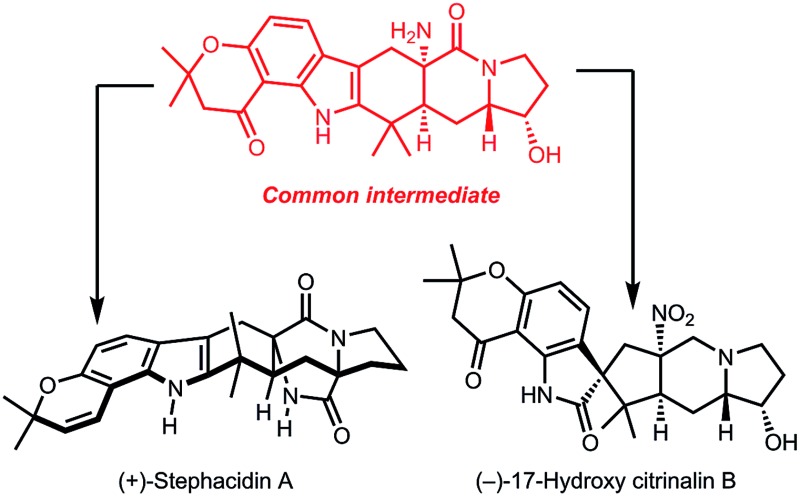
The first strategy that provides reverse-prenylated indole alkaloids that bear a characteristic bicyclo[2.2.2]diazaoctane as well as those that lack this structural motif is reported.

## Introduction

Historically, the undertaking of total syntheses of natural products has focused on ‘target-oriented’ syntheses whereby a single compound is targeted for synthesis to investigate its biological relevance or aspects of its structure.^1^ This practice has inspired many new synthesis developments. Recently, however, exercises in complex molecule total synthesis are placing a growing emphasis on the preparation of diverse molecular skeletons from a common intermediate.^2^ This practice, which mirrors the biological production of many secondary metabolites *but does not necessarily follow along biosynthetic lines*, maximizes the opportunities for, and efficiency of, accessing molecular diversity to facilitate structure–activity relationship studies. Over the last 30 years, this concept has led to remarkable unified strategies for the syntheses of various families of natural products.^3^ Here, we present the extension of this idea to the syntheses of congeners in the prenylated indole alkaloid family which features a powerful Dieckmann-type cyclization to forge a key [2.2.2]bicycle.

**Fig. 1:**
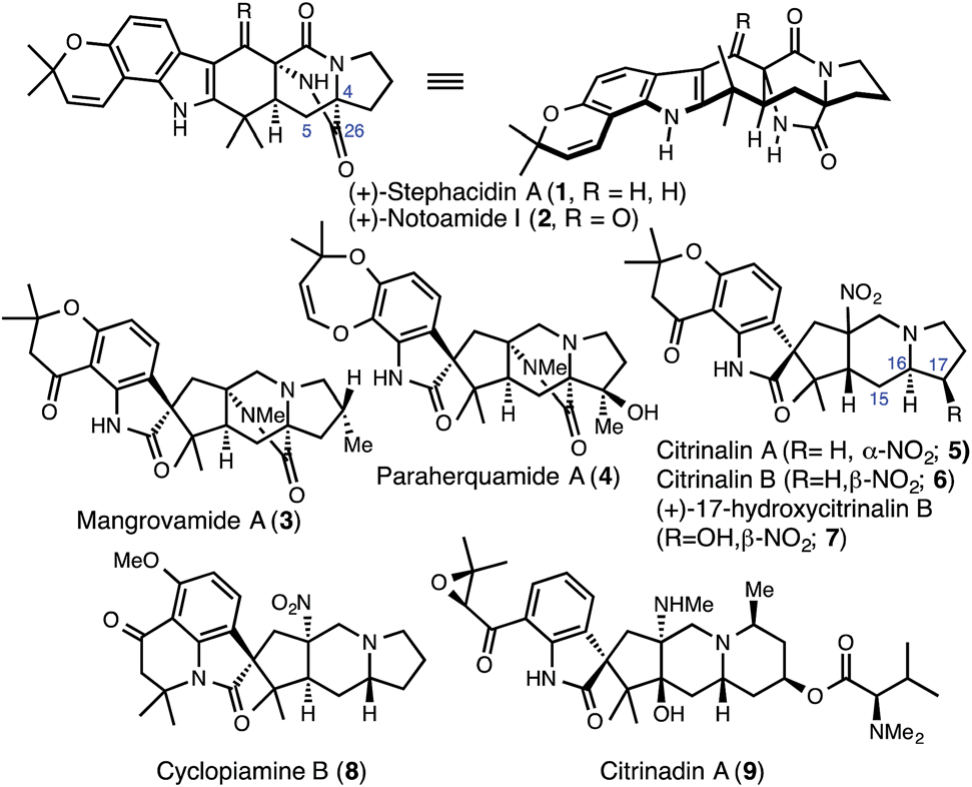
Selected prenylated indole alkaloids.

The prenylated indole alkaloids include some of the most structurally diverse secondary metabolites isolated to date (see Fig. 1 for selected examples). Many congeners such as stephacidin A (**1**), notoamide I (**2**), mangrovamide A (**3**) and paraherquamide A (**4**) contain a bicyclo[2.2.2]diazaoctane structural moiety.^4^ Over the last decade however, additional members of the family that lack the bicyclo[2.2.2]diazaoctane core have begun to emerge. This includes the citrinalins (*e.g.*, **5**), citrinadins (*e.g.*, **9**) and the cyclopiamines (*e.g.*, **8** – albeit isolated in 1979).^5^ While myriad bioactivity has been discovered for various prenylated indole alkaloids (especially anthelmintic activity),^4^ the recent emergence of the citrinadins and related compounds^6^ that lack the bicyclo[2.2.2]diazaoctane structural motif as potent anti-tumor compounds has heightened interest in the whole family of secondary metabolites.

## Results and discussion

**Scheme 1:**
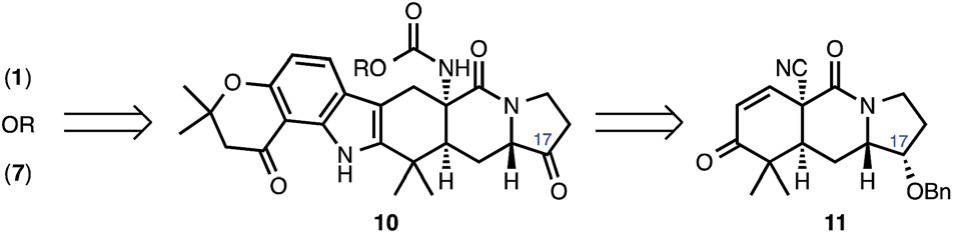
Unified retrosynthetic approach.

From our perspective, a unified synthetic approach that affords prenylated indole alkaloid congeners bearing the bicyclo[2.2.2] diazaoctane core as well as those lacking this structural moiety would provide the most *strategically efficient* approach to these natural products. However, to date, such an approach has not been reported. All the existing syntheses of this family of molecules have targeted either the subset that contains the [2.2.2] diazaoctane bicycle or those molecules that lack this structural feature.^7^,^8^ In this manuscript, we present our studies toward identifying a common intermediate that can be advanced to natural products representative of both prenylated indole alkaloid structural motifs. These studies have led to the identification of **10** (Scheme 1) as such a common intermediate, which now provides the first total synthesis of (–)-17-hydroxy-citrinalin B (**7**) as well as a synthesis of (+)-stephacidin A (**1**) and (+)-notoamide I (**2**). Our synthetic strategy to these two natural products, which rests on ‘network analysis’^9^ considerations, diverges only at a late stage. Thus, strategic bond disconnection of the maximally bridging ring in, for example, **1** (*i.e.*, the 2,5-diketopiperazine ring) leads back to carbamate **10**, where a bond can be formed at a late stage between C16 and the carbamate carbonyl group.^10^ In this way, the two sub-families of the prenylated indole alkaloids (*e.g.*, **1** and **7**) can be connected by a synthesis sequence characterized by a progressive increase in structural complexity, which distinguishes this approach from prior syntheses of related prenylated indole alkaloids.^7^ Hexacycle **10** can in turn arise from tricycle **11** using an indole annulation reaction, which would provide opportunities to prepare other natural products such as paraherquamide A (**4**) that differ in their indole substitution pattern.

A general strategy for the synthesis of the prenylated indole alkaloids that encompasses the two main structural types (of which **1** and **7** are representative) has not been explored before. In all of the previous syntheses of the bicyclo[2.2.2] diazaoctane bearing congeners, the tetrasubstituted center at the bicyclo[2.2.2] bridgehead (*e.g.*, C4 – stephacidin numbering – in **1**) is constructed at an early stage or through C4–C5 bond formation, which would necessitate its late-stage cleavage (in a complexity minimizing manipulation) in order to form compounds such as **7** from **1**. In this latter scenario, an amide hydrolysis of the bicyclo[2.2.2]diazaoctane, decarboxylation, and a *diastereoselective* protonation at the ring junction (the diastereoselectivity of which is not certain outside of an enzyme pocket)^11^ would be required for a conversion of **1** to the sub-family that lacks the diazaoctane structural motif. Our approach to this collection of molecules is complementary.

### Synthesis of divergent intermediate **23**

**Scheme 2:**
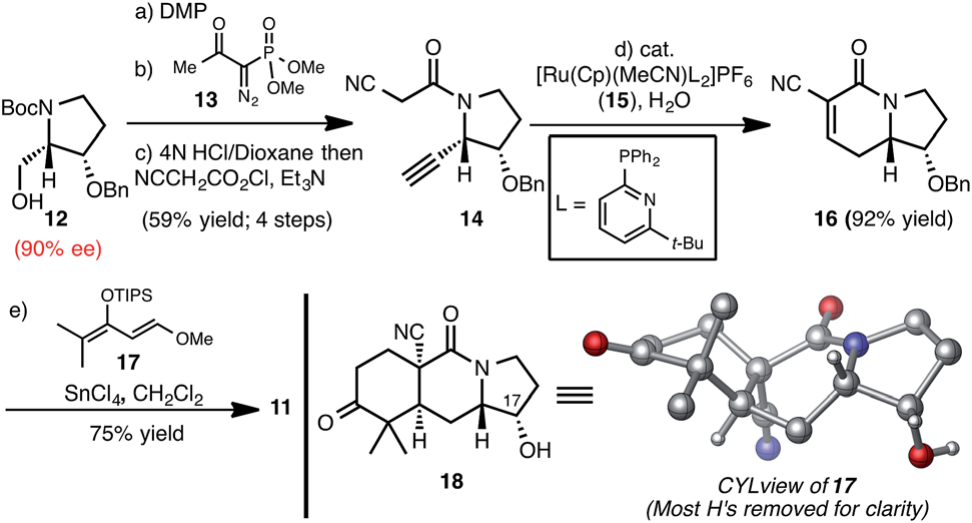
Preparation of **11**. (a) DMP (1.1 equiv.), NaHCO_3_, CH_2_Cl_2_, 0 °C to rt, 2.5 h, >87%; (b) **13** (1.5 equiv.), NaOMe (5.0 equiv.), THF, –78 °C to –50 °C, 2 h, 91%; (c) 4 N HCl/dioxane (4.0 equiv.), 0 °C to rt, 30 min; concentrate; then cyanoacetyl chloride (2.5 equiv.), Et_3_N (2.5 equiv.), 0 °C to rt, 3 h, 75%; (d) **15** (8 mol%), acetone/H_2_O, 70 °C, 24 h, 92%; (e) **17** (2.66 equiv.), SnCl_4_ (1.2 equiv.), CH_2_Cl_2_, –78 °C to –42 °C, 30 min, then rt, 30 min, 75%. DMP = Dess–Martin periodinane; Bn = benzyl.

Our studies commenced with the preparation of **11** (Scheme 2), which is available in 6 steps from known enantioenriched alcohol **12**.^12^ Analogous to our previously established sequence,8*d* oxidation of the alcohol group of **12** and alkynylative homologation of the resulting aldehyde using the Ohira–Bestmann reagent (**13**) affords an alkyne, which upon Boc-cleavage and acylation with α-cyano acetylchloride gives alkyne **14**.^13^ A formal cycloisomerization of **14**, presumably proceeding by anti-Markovnikov hydration of the terminal alkyne followed by Knoevanagel condensation of the incipient aldehyde, was effected using the Grotjahn complex (**15**)^14^ to yield bicycle **16**. Diels–Alder cycloaddition of **16** with diene **17**, facilitated by SnCl_4_ gives enone **11** upon basic workup. Even though we had previously accomplished the analogous synthesis of a tricycle lacking the benzyloxy group at C17 (see numbering in **11**), it was unclear what influence this added substituent would exert on the diastereoselectivity of the cycloaddition step and so we were gratified to obtain diastereomer **11** in good yield. The structure of **11** is supported by the X-ray structure of keto-alcohol **18** (see CYLview in Scheme 2), which we obtained following hydrogenation and BBr_3_-mediated cleavage of the benzyl group of **11**.^15^

**Scheme 3:**
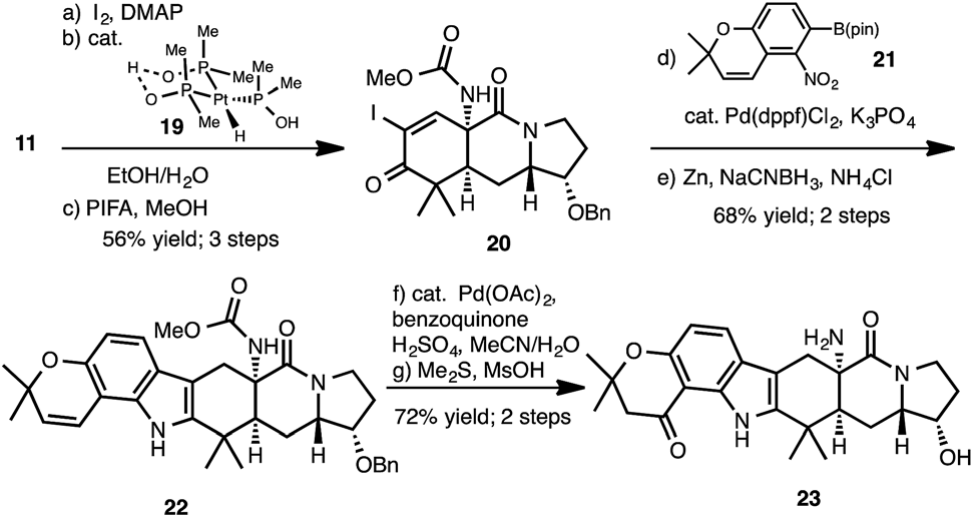
Synthesis of fused hexacycle **23**. (a) Iodine (3.0 equiv.), DMAP (3.0 equiv.), pyridine/CCl_4_, 60 °C, 22 h, 77%; (b) **19** (30 mol%), EtOH/H_2_O, rt, 4 days, 82%; (c) PIFA (1.1 equiv.), MeOH, 0 °C to rt, 16 h, 89%; (d) **21** (1.5 equiv.), dppfPdCl_2_ (10 mol%), K_3_PO_4_ (3.75 equiv.), DMF, 40 °C, 16 h, 94%; (e) Zn (excess), NaCNBH_3_ (5.0 equiv.), sat. aq. NH_4_Cl, MeOH, rt, 2 h, 71%; (f) Pd(OAc)_2_ (0.40 equiv.), benzoquinone (1.5 equiv.), cat. H_2_SO_4_, MeCN/H_2_O, rt, 17 h, 77%; (g) Me_2_S (20 equiv.), MsOH, 40 °C, 15 h, 93%. DMAP = 4-dimethylaminopyridine; PIFA = [bis(trifluoroacetoxy)iodo]benzene; dppf = 1,1′-bis(diphenylphosphino)ferrocene; DMF = dimethylformamide; Ms = methanesulfonyl; Bn = benzyl.

A Johnson iodination^16^ of **11** (Scheme 3), followed by hydration of the nitrile group using the Ghaffar–Parkins complex (**19**)^17^ and subsequent Hofmann rearrangement of the resulting carboxamide using phenyliodoso trifluoromethyl acetate (PIFA) in the presence of methanol provided carbamate **20**.^18^ Following the precedent of Myers and Herzon,7*d* Suzuki cross-coupling of iodide **20** with pincacol boronic ester **21** followed by reductive cyclization yielded indole annulated hexacycle **22**. At this stage, Wacker oxidation^19^ of the chromene moiety and treatment of the resulting chromanone with dimethyl sulfide in the presence of methane sulfonic acid unveiled the amine and hydroxyl groups to provide **23**, which would serve as the common intermediate to access both 17-hydroxy-citrinalin B as well as stephacidin A. Of note, while the synthesis of 17-hydroxy-citrinalin B would take advantage of the chromanone unit, a synthesis of stephacidin A from **23** would require a reconstitution of the chromene moiety. However, in our hands, the chromanone moiety proved to be more robust (as compared to the chromene) in many of the subsequent steps and so **23** served as a more effective intermediate even to stephacidin A.

### Synthesis of (–)-17-hydroxy-citrinalin B (**7**) from **23**

**Scheme 4:**
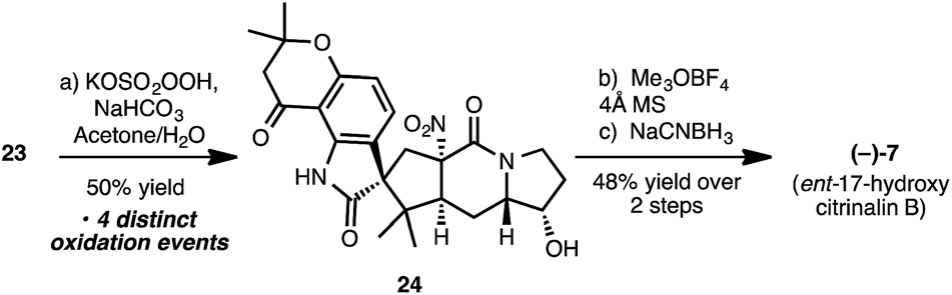
Completion of the synthesis of 17-hydroxy-citrinalin B (**7**). (a) Oxone® (11.4 equiv.), NaHCO_3_, acetone/H_2_O, 0 °C to rt, 2 h, 50%; (b) Me_3_OBF_4_ (12 equiv.), CH_2_Cl_2_, 4 Å MS, 45 °C, 16 h, then NaCNBH_3_ (excess), MeOH, 0 °C, 30 min, 48%. Oxone® = potassium peroxomonosulfate; MS = molecular sieves.

To complete a synthesis of 17-hydroxy-citrinalin B, **23** was subjected to oxidation using oxone as previously described in the synthesis of citrinalin B by our group (Scheme 4).8*d* The ensuing series of remarkable chemoselective oxidations accomplished the conversion of the indole to the spirooxindole (corresponding to the desired diastereomer for **7**) as well as the oxidation of the amino group to the nitro group – all in the presence of the free secondary hydroxy group.^20^

With **24** in hand, chemoselective reductive removal of the tertiary amide carbonyl group (in the presence of several other groups that are susceptible to reduction) was accomplished following an adaptation of a procedure first reported by Borch.^21^ Thus, subjection of **24** to Me_3_OBF_4_ followed by NaCNBH_3_ proceeded in respectable yield to give (–)-17-hydroxy-citrinalin B.^22^ Of note, in our hands, it was the TFA salt of **7** that provided analytical data identical in all respects to that of the natural isolate, which had been reported as the neutral compound.8*d*

Although our synthetic sequence for the preparation of 17-hydroxy-citrinalin B mirrors closely our previous synthesis of citrinalin B (**6**),^23^ it required a much more stringent level of chemoselectivity in the endgame. The success of this route is a testament to the robustness of our synthetic plan, which proceeded without event (especially in the endgame) even in the presence of a free hydroxyl group at C17 from intermediate **23** onwards.

### Synthesis of (+)-stephacidin A (**1**) and (+)-notoamide I (**2**) from **23**

**Scheme 5:**
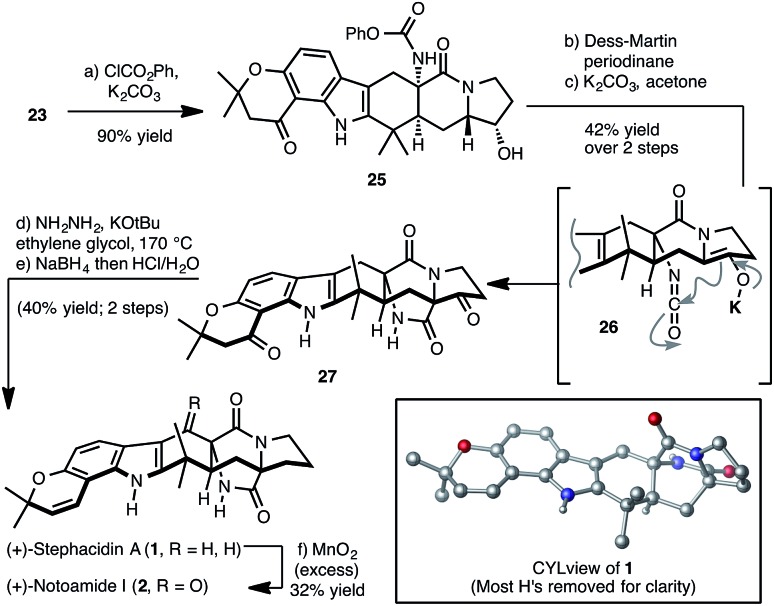
Completion of the synthesis of (+)-stephacidin A (**1**) and (+)-notoamide I (**2**). (a) ClCO_2_Ph (excess), K_2_CO_3_, acetone, rt, 16 h, 90%; (b) DMP (1.5 equiv.), NaHCO_3_, CH_2_Cl_2_, rt, 20 min; (c) K_2_CO_3_, acetone, 50 °C, 2 h, 42% over two steps; (d) NH_2_NH_2_ (1.1 equiv.), ethylene glycol, 70 °C, 17 h, then *t*-BuOK (5.0 equiv.), 170 °C, 2 h, 57%; (e) NaBH_4_ (10.0 equiv.), THF, 16 h, then 0.6 N HCl/H_2_O, 60 °C, 30 min, 71%. (f) MnO_2_ (excess), EtOAc, rt, 30 min, 32%. DMP = Dess–Martin periodinane; THF = tetrahydrofuran.

In an initial demonstration of the utility of **23** as an intermediate for the synthesis of prenylated indole alkaloid congeners possessing the bicyclo[2.2.2]diazaoctane structural motif, we have completed a synthesis of (+)-stephacidin A and (+)-notoamide I as outlined in Scheme 5. Thus chemoselective carbamoylation of the primary amine of **23** was achieved in high yield in the presence of the secondary hydroxyl to afford phenyl carbamate **25**. At this point, oxidation of the hydroxy group and treatment of the resulting ketone with K_2_CO_3_ forges the bicyclo[2.2.2]diazaoctane framework of stephacidin A, presumably through a Dieckmann condensation *via* isocyanate/enolate intermediate **26**.^24^ These exceedingly simple conditions effectively accomplish the task of synthetically connecting the two major sub-families of the prenylated indole alkaloids. From our perspective, polycycle **27** represents a versatile framework that may be advanced to myriad prenylated indole alkaloids including mangrovamide A (**3**) and paraherquamide A (**4**). To complete the synthesis of (+)-stephacidin A, removal of the ketone group in the pyrrolidine ring using a Wolff–Kishner protocol followed by reduction of the chromanone carbonyl group and elimination of the resulting hydroxyl gave (+)-stephacidin A (**1**) in 40% yield over the two steps.^25^ Analytical data for synthetic stephacidin A prepared by us matched perfectly previously reported spectra.7a,e Furthermore, our X-ray analysis of a single crystal of **1** provided unambiguous support for the structure of the natural isolate (see CYLview in Scheme 5). Stephacidin A is a versatile starting point for the preparation of other prenylated indole alkaloids. For example, **1** was easily converted to (+)-notoamide I (**2**) upon treatment with MnO_2_ in EtOAc (32% yield). Our synthesis of **1** also constitutes formal syntheses of (–)-notoamide B, (+)-avrainvillamide and (–)-stephacidin B.^26^

## Conclusions

In conclusion, we have achieved the first unified approach to the two sub-families of the prenylated indole alkaloids (*i.e.*, that either lack or possess the bicyclo[2.2.2]diazaoctane structural motif). Our strategy has been exemplified with the first preparation of the natural product (–)-17-hydroxy-citrinalin B and of (+)-stephacidin A. Key to the success of the approach was the identification of a late-stage common intermediate (**23**), which could be advanced to either subclass of the prenylated indole alkaloids using a remarkably diastereoselective spiro-oxindole formation attended by a chemoselective oxidation of an amino group to a nitro group. Our synthesis of stephacidin A also featured a complexity building isocyanate capture to forge a [2.2.2]bicycle. Our studies now set the stage for the broad-ranging syntheses of congeners of the prenylated indole alkaloid family to facilitate in-depth studies on their biosynthesis and biological activity.

## Supplementary Material

Supplementary information

Crystal structure data
