# Supplementary material for: Unified approach to prenylated indole alkaloids: total syntheses of (–)-17-hydroxy-citrinalin B, (+)-stephacidin A, and (+)-notoamide I
Source: Chem Sci. 2015 Jun 18;6(8):5048–52. doi: 10.1039/c5sc01977j (PMC4583210; doi:10.1039/c5sc01977j)

Electronic Supplementary Material (ESI) for Chemical Science.
This journal is © The Royal Society of Chemistry 2015

Supporting Information for:

Unified Approach to Prenylated Indole Alkaloids: Total Syntheses of (–)-17-hydroxy-Citrinalin B, (+)-Stephacidin A, and (+)-Notoamide I.

Eduardo V. Mercado-Marin and Richmond Sarpong*

Department of Chemistry, University of California, Berkeley, California 94720, USA.

*Corresponding Author: Richmond Sarpong (rsarpong@berkeley.edu)

Table of Contents:

I. General Experimental for the Synthesis of Compounds 11–27…………......…………….....03

II. Experimental Procedures and Characterization Data for Compounds 11–27……...…..04

III. Crystallographic Data for Compounds 1 and 18………...……………..…………………...28

IV. ^{1}H & ^{13}C NMR Spectra for Compounds 11–27…....…………………………………......47

I. General Experimental for the Synthesis of Compounds 11–27:

Unless otherwise noted, all reactions were carried out under an atmosphere of nitrogen, and all reagents were purchased from commercial suppliers and used without further purification. All reactions were carried out in flame-dried glassware under a positive pressure of nitrogen in dry solvents using standard Schlenk techniques. Tetrahydrofuran (THF), diethyl ether (Et_2O), benzene, toluene (PhMe), methanol (MeOH) and triethylamine (Et_3N) were dried over alumina under an argon atmosphere in a GlassContour solvent system. Dichloromethane (CH_2Cl_2) was distilled over calcium hydride under a nitrogen atmosphere. All other solvents and reagents were used as received unless otherwise noted. Reaction temperatures above room temperature (RT), 23 °C, were controlled by an IKA® temperature modulator. Reaction progress were monitored by thin layer chromatography using SiliCycle silica gel 60 F254 precoated plates (0.25 mm) which were visualized using UV light (254 nm) and ninhydrin or $KMnO_4$ stain. Sorbtech silica gel (particle size 40-63 μm) was used for flash chromatography. Melting points were recorded on a Mel-Temp II by Laboratory Devices Inc., USA. Optical rotation was recorded on a Perkin Elmer Polarimeter 241 at the D line (1.0 dm path length), *c* = mg/mL. ^1H and ^{13}C NMR were recorded on a Bruker AV-600 MHz spectrometer with ^{13}C operating frequency of 150 MHz, in $CDCl_3$, CD_3OD or $(CD_3)_2SO$ at 23 °C. Chemical shifts (δ) are reported in ppm relative to the residual solvent signal ($CDCl_3$ δ = 7.26 for ^1H NMR and δ = 77.16 for ^{13}C NMR; CD_3OD δ = 3.35 for ^1H NMR and δ = 49.3 for ^{13}C NMR $(CD_3)_2SO$ δ = 2.50 for ^1H NMR and δ = 39.52 for ^{13}C NMR). Data for ^1H NMR are reported as follows: chemical shift (multiplicity, coupling constant, number of hydrogens). Multiplicity is abbreviated as follows: s (singlet), d (doublet), t (triplet), q (quartet), m (multiplet), br (broad). IR spectra were recorded on either a Nicolet MAGNA-IR 850 spectrometer or a Bruker Alpha Platinum ATR spectrometer and are reported in frequency of absorption (cm^{-1}). High-resolution mass spectral data were obtained from the University of California, Berkeley Mass Spectral Facility, on a VG Prospec Micromass spectrometer for EI.

II. Experimental Procedures and Characterization Data for Compounds 11–27

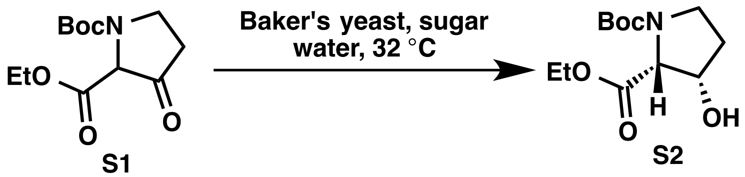

This procedure was adapted from a known procedure.[1] To a 2 L Erlenmeyer flask was added in succession 1-(*tert*-butyl) 2-ethyl 3-oxopyrrolidine-1,2-dicarboxylate[2] (**S1)** (12.0 g, 46.7 mmol, 1.00 equiv), sugar (Trader Joe's Organic sugar™, 185 g, 1.03 mol, 22 equiv), and distilled water (940 mL, 0.05M). The mixture was placed in a water bath preheated to 32 °C and stirred until all the sugar had dissolved after which dry Baker's yeast (120 g, Red Star™) was added in one portion. The resulting mixture was stirred at 32 °C for 24 h then filtered using a Büchner funnel. The aqueous layer was extracted with ethyl acetate (5 x 700 mL) and the combined organic extracts were dried over Na_2SO_4, filtered, and concentrated *in vacuo*. The crude oil was purified by silica gel chromatography (150 mL SiO_2 with 2:3 ethyl acetate:hexanes) to yield **S2** (9.65 grams, 37.2 mmol, 80%) as a clear, colorless oil. TLC (ethyl acetate:hexanes, 3:2 v/v): R_f=0.28; **^{1}H NMR** (600 MHz, $CDCl_3$, mixture of amide conformers 1:2) δ = 4.60–4.52 (m, 1H), 4.36 (d, *J* = 6.8 Hz, 0.33H), 4.29 (d, *J* = 6.8 Hz, 0.66H), 4.25 – 4.12 (m, 2H), 3.65 – 3.54 (m, 1H), 3.48 – 3.36 (m, 1H), 3.08 (br s, 1H), 2.11 – 2.02 (m, 1H), 2.02 – 1.93 (m, 1H), 1.42 (s, 3H), 1.38 (s, 6H), 1.29 – 1.22 (m, 3H); **^{13}C NMR** (150 MHz, $CDCl_3$, mixture of amide conformers 1:2) δ = 170.5, 170.3, 154.3, 153.8, 80.1, 80.0, 72.2, 71.3, 63.9, 63.4, 61.1, 61.0, 44.2, 43.7, 32.6, 32.1, 28.3, 28.2, 14.2, 14.1; $[\alpha]^{25}_D$ + 21.6 (c 1.21, CH_2Cl_2). The spectroscopic data were consistent with those previously reported.[1,2]

---

[1] R. M. Williams, J. Cao, H. Tsujishima, R. J. Cox, *J. Am. Chem. Soc.,* 2003, **125**, 12172.

[2] (a) J. Cooper, P. T. Gallagher, D. W. Knight, *J. Chem. Soc. Chem. Commun.,* 1988, **8**, 509. (b) J. Cooper, P. T. Gallagher, D. W. Knight, *J. Chem. Soc. Perkin. Trans. 1*, 1993, 1313. Note: The methyl ester of **S1** was previously reported by Sorenson and co-workers by an intramolecular N-H insertion of a diazo-compound (R. Moreau, E. J. Sorensen, *Tetrahedron*, 2007, **63**, 6446.); this method was employed on large scale to access **S1** by substituting methyl potassium malonate for ethyl potassium malonate in accordance with Sorensen's procedure.

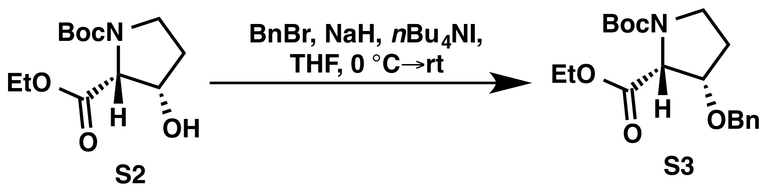

S2   S3

To a solution of 1-(*tert*-butyl) 2-ethyl (2*R*,3*S*)-3-hydroxypyrrolidine-1,2-dicarboxylate **(S2)** (9.65 g, 37.2 mmol, 1.00 equiv), tetrabutylammonium iodide (3.71 g, 11.2 mmol, 0.30 equiv), and benzyl bromide (6.65 mL, 55.9 mmol, 1.5 equiv) in THF (250 mL, 0.15M) at 0 °C was added NaH (60% dispersion in mineral oil, 1.64 g, 40.9 mmol, 1.10 equiv) in three equal portions. The reaction mixture was slowly warmed to room temperature by allowing the ice bath to expire. After 4 h, additional benzyl bromide (4.0 mL, 16.2 mmol, 0.44 equiv) was added at room temperature and the resulting solution stirred for 15 h. The reaction was quenched by the addition of ice-cold water (100 mL) and the aqueous layer was extracted with ethyl acetate (4 x 150 mL). The combined organic layers were washed with brine (1 x 200 mL), dried over Na_2SO_4, filtered, and concentrated *in vacuo*. The crude oil was purified by silica gel chromatography (250 mL SiO_2 with 1:9 ethyl acetate:hexanes) to yield **S3** (10.5 g, 30.1 mmol, 81%) as a clear, colorless oil. TLC (ethyl acetate:hexanes, 1:2 v/v): R_f=0.42; ^{1}**H NMR** (600 MHz, $CDCl_3$, mixture of amide conformers 1:2) δ = 7.36 – 7.28 (m, 5H), 4.73 – 4.62 (m, 1H), 4.61 – 4.54 (m, 1.4H), 4.47 (d, J = 7.3 Hz, 0.6H), 4.35 – 4.13 (m, 3H), 3.73 – 3.60 (m, 1H), 3.41 – 3.31 (m, 1H), 2.22 – 2.11 (m, 1H), 2.10 – 2.02 (m, 1H), 1.46 (s, 3H), 1.43 (s, 6H), 1.28 – 1.19 (m, 3H); ^{13}**C NMR** (150 MHz, $CDCl_3$, mixture of amide conformers 1:2) δ = 170.1, 170.0, 154.3, 153.8, 137.7, 137.6, 128.4, 128.3, 127.7, 127.6, 127.4, 80.1, 80.0, 78.8, 77.9, 72.1, 72.0, 62.0, 61.3, 60.9, 60.8, 43.9, 43.4, 29.8, 29.1, 28.4, 28.3, 28.2, 14.3, 14.1; **IR** (NaCl, thin film) ν_{max}: 3065, 2978, 1744, 1708, 1693, 1455, 1402 cm^{-1}; **HRMS** (ESI) calcd for $C_{19}H_{28}O_5N$ ($[M]^+$), 350.1962; found, 350.1968.

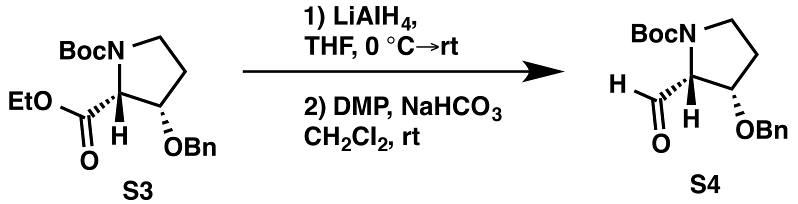

To a solution of 1-(*tert*-butyl) 2-ethyl (2*R*,3*S*)-3-(benzyloxy)pyrrolidine-1,2-dicarboxylate (**S3**) (10.5 g, 30.1 mmol, 1.0 equiv) in THF (250 mL, 0.1M) cooled to 0 °C was added $LiAlH_4$ (2.85 g, 75.2 mmol, 2.5 equiv) in four equal portions. The resulting solution was stirred at 0 °C for 50 min then diluted with Et_2O (250 mL). The solution was cooled to 0 °C then 4.2 mL of distilled water was added dropwise, followed by 4.2 mL of 15% aqueous NaOH. After 5 min, 12.5 mL of distilled water was added and the solution was warmed to room temperature and stirred for 30 min. $MgSO_4$ (50 g) was then added and the solution was stirred at room temperature for 1.5 h, then filtered, and concentrated *in vacuo*. Analysis of the crude oil gave spectroscopic data consistent with those previously reported.[3] [**^{1}H NMR** (600 MHz, $CDCl_3$, mixture of amide conformers 1:2) δ = 7.38 – 7.28 (m, 5H), 4.68 – 4.56 (m, 1H), 4.54 – 4.45 (m, 1H), 4.35 – 4.22 (m, 1H), 4.19 – 4.10 (m, 0.7H), 4.02 – 3.71 (m, 3.3H), 3.49 – 3.32 (m, 2H), 2.73 (br s, 0.5H), 2.12 (br s, 0.5H), 2.05 – 1.88 (m, 2H), 1.46 (s, 9H); **^{13}C NMR** (150 MHz, $CDCl_3$, mixture of amide conformers 1:2) δ = 156.1, 154.3, 137.6, 137.3, 128.5, 128.4, 127.9, 127.8, 127.5, 80.0, 79.9, 79.5, 78.7, 71.9, 71.6, 63.2, 61.9, 61.8, 59.0, 44.4, 43.3, 29.2, 28.3.] The crude oil was dissolved in CH_2Cl_2 (170 mL, 0.18M) and $NaHCO_3$ (12.6 g, 150.3 mmol, 5.0 equiv) was added. The resulting solution was cooled to 0 °C and Dess-Martin periodinane (DMP) (14.0 g, 33.1 mmol, 1.1 equiv) was added in three equal portions. After 2.5 h, the resulting yellow solution was warmed to room temperature then poured into a separatory funnel containing 600 mL (1:1 v/v) saturated aqueous $NaHCO_3$ and saturated aqueous $Na_2S_2O_3$ and the layers separated. The aqueous layer was extracted with ethyl acetate (3 x 300 mL) and the combined organic extracts

[3] T. K. Chakraborty, P. Srinivasu, R. V. Rao, S. K. Kumar, A. C. Kunwar, *J. Org. Chem.,* 2004, **69**, 7399.

were dried over Na_2SO_4, filtered, and concentrated *in vacuo*. The crude oil was purified by silica gel chromatography (200 mL SiO_2 with 1:4 ethyl acetate:hexanes) to yield **S4** (7.95 g, 26.1 mmol, 87% over 2 steps) as a clear, colorless oil. TLC (ethyl acetate:hexanes, 1:1 v/v): R_f=0.52; **^{1}H NMR** (600 MHz, $CDCl_3$, mixture of amide conformers 1:2) δ = 9.56 (d, *J* = 2.4 Hz, 0.33H), 9.50 (d, *J* = 3.6 Hz, 0.66H), 7.38 – 7.23 (m, 5H), 4.58 – 4.52 (m, 1H), 4.50 – 4.40 (m, 2H), 4.27 – 4.21 (m, 0.33H), 4.15 – 4.09 (m, 0.66H), 3.73 – 3.61 (m, 1.66H), 3.58 – 3.51 (m, 0.33H), 2.16 – 2.07 (m, 1H), 2.00 – 1.88 (m, 1H), 1.47 (s, 3H), 1.41 (s, 6H); **^{13}C NMR** (150 MHz, $CDCl_3$, mixture of amide conformers 1:2) δ = 200.1, 154.7, 153.8, 137.2, 128.4, 127.9, 127.6, 127.5, 81.4, 80.8, 80.4, 80.2, 71.8, 71.6, 67.9, 67.6, 44.6, 44.5, 30.4, 29.8, 28.3, 28.2, 28.0; **IR** (NaCl, thin film) ν_{max}: 3032, 2977, 1737, 1704, 1455, 1397, 1367 cm^{-1}; **HRMS** (ESI) calcd for $C_{17}H_{23}O_4N_1Na$ ($[M+Na]^+$): 328.1519; found 328.1524.

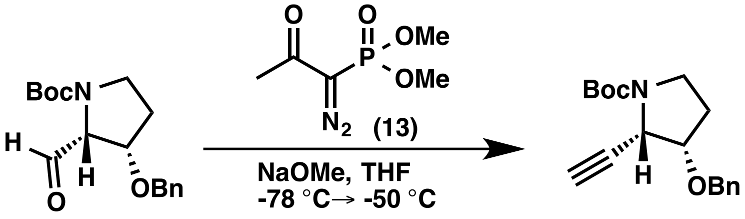

S4　　　　S5

A solution of dimethyl (1-diazo-2-oxopropyl)phosphonate (**13**)[4] (15.04 g, 78.3 mmol, 1.5 equiv) in THF (200 mL, 0.39M) was added via cannula to a stirring suspension of NaOMe (13.5 g, 261 mmol, 5.0 equiv) in THF (200 mL, 1.30M) at –78 °C (dry ice/acetone) and stirred for 30 min. To this solution was added a cooled solution (–78 °C) of *tert*-butyl (2*R*,3*S*)-3-(benzyloxy)-2-formylpyrrolidine-1-carboxylate (**S4**) (15.93 g, 52.2 mmol, 1.0 equiv) in THF (200 mL, 0.26M) via cannula along the side of the flask. The resulting solution was slowly warmed to –50 °C by allowing the dry ice/acetone bath to expire. Dry ice was added as needed to maintain a temperature of ≤ –50 °C. After TLC analysis indicated complete consumption of the starting material, saturated aqueous $NaHCO_3$ (400 mL) was added followed by Et_2O (500 mL). The solution was warmed to room temperature, the layers were separated, and the aqueous layer was extracted with Et_2O (3 x 500 mL). The combined organic extracts were dried over $MgSO_4$, filtered, and concentrated *in vacuo*. The crude yellow oil was purified by silica gel chromatography (300 mL SiO_2 with 1:9 ethyl acetate:hexanes) to yield **S5** (14.27 g, 47.4 mmol,

[4] S. Ohira, *Synth. Commun.*, 1989, **19**, 561.

91%) as a white solide. M.P. 111-113 °C; TLC (ethyl acetate:hexanes, 1:1 v/v): R_f=0.75; **^{1}H NMR** (600 MHz, $CDCl_3$, mixture of amide conformers ~1:1) δ = 7.39 (d, *J* = 7.4 Hz, 2H), 7.35 (t, *J* = 7.4, 7.3 Hz, 2H), 7.30 (t, *J* = 7.3, 7.3 Hz, 1H), 4.76 – 4.69 (m, 1.5H), 4.60 (d, *J* = 6.2 Hz, 0.5H), 4.56 – 4.49 (m, 1H), 4.06 – 3.96 (m, 1H), 3.56 – 3.44 (m, 1H), 3.29 – 3.21 (m, 1H), 2.39 (s, 0.5H), 2.35 (s, 0.5H), 2.18 – 2.08 (m, 2H), 1.51 – 1.42 (m, 9H); **^{13}C NMR** (150 MHz, $CDCl_3$, mixture of amide conformers ~1:1) δ = 154.1, 137.6, 128.6, 128.1, 128.0, 80.3, 80.2, 79.9, 77.8, 77.4, 77.2, 77.02, 76.95, 73.3, 72.9, 72.2, 50.8, 50.2, 42.9, 42.3, 29.6, 28.8, 28.5; **IR** (NaCl, thin film) ν_{max}: 3288, 2978, 2892, 1694, 1497, 1477, 1455, 1393 cm^{-1}; **HRMS** (ESI) calcd for $C_{18}H_{23}O_3N_1Na$ ($[M+Na]^+$): 324.1570, found 324.1569.

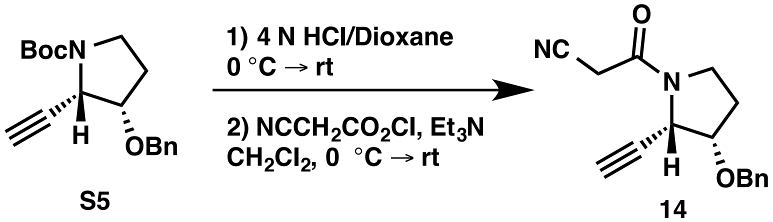

To a flask charged with *tert*-butyl (2*S*,3*S*)-3-(benzyloxy)-2-ethynylpyrrolidine-1-carboxylate (**S5**) (14.2 g, 47.2 mmol, 1.0 equiv) was added 4 N HCl/dioxane (60 mL, 236 mmol, 5.0 equiv) dropwise at 0 °C. The resulting solution was then warmed to room temperature and stirred for 30 min at which point the solvent was removed *in vacuo*. The excess HCl/dioxane was removed by azeotropic distillation with Et_2O (2 x 100 mL) and then hexanes (2 x 100 mL) to give a beige solid which was dried *in vacuo* overnight. The resulting crude mixture was suspended in CH_2Cl_2 (100 mL) and Et_3N (16.5 mL, 118 mmol, 2.5 equiv) was added dropwise at 0 °C, followed by the dropwise addition of cyanoacetylchloride (12.2 g, 118 mmol, 2.5 equiv) as a solution in CH_2Cl_2 (60 mL, 1.97 M). The resulting red solution was stirred at 0 °C for 2 h then warmed to room temperature and stirred for an additional 1 h. Saturated aqueous $NaHCO_3$ (200 mL) was added and the layers were separated. The aqueous layer was extracted with ethyl acetate (4 x 250 mL) and the combined organic extracts were dried over Na_2SO_4, filtered, and concentrated *in vacuo*. The crude red oil was purified by silica gel chromatography (400 mL SiO_2 with 2:3 to 3:2 ethyl acetate:hexanes) to yield **14** (9.52 g, 35.5 mmol, 75% over 2 steps) as an orange solid. M.P. 123-124 °C; TLC (ethyl acetate:hexanes, 4:1 v/v): R_f=0.47; **^{1}H NMR** (600 MHz, $CDCl_3$, mixture of amide conformers ~2:3) δ = 7.41 – 7.30 (m, 5H), 4.94 (dd, *J* = 6.4, 2.3 Hz, 0.4H), 4.74 (d, *J* = 11.6 Hz, 0.4H), 4.71 (d, *J* = 11.6 Hz, 0.6H), 4.64 – 4.58 (m, 1.2H), 4.55 (d, *J* = 11.6 Hz, 0.4H), 4.18 (dt, *J* = 9.1, 6.2 Hz, 0.6H), 4.06 (dt, *J* = 9.7, 6.3 Hz, 0.4H), 3.76 – 3.59 (m, 2H), 3.50 – 3.37

(m, 2H), 2.60 (d, J = 2.4 Hz, 0.6H), 2.44 (d, J = 2.2 Hz, 0.4H), 2.37 – 2.21 (m, 1H), 2.21 – 2.08 (m, 1H); **^{13}C NMR** (150 MHz, $CDCl_3$, mixture of amide conformers ~2:3) δ = 160.9, 160.4, 137.2, 137.1, 128.68, 128.65, 128.3, 128.2, 128.1, 128.0, 113.9, 113.5, 78.3, 77.9, 77.7, 76.6, 76.4, 74.3, 72.58, 72.5, 51.71, 50.67, 44.1, 43.7, 29.9, 28.4, 25.8, 25.4; **IR** (NaCl, thin film) ν_{max}: 3273, 3248, 2939, 2888, 2361, 1663, 1454, 1434, 1399 cm^{-1}; **HRMS** (ESI) calcd for $C_{16}H_{16}O_2N_2Na$ ([M+Na]$^+$): 291.1104, found 291.1103.

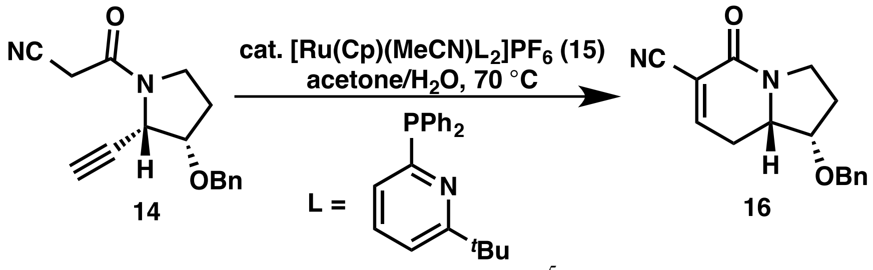

This procedure was adapted from a known procedure.[5] To a Schlenk flask charged with 3-((2*S*,3*S*)-3-(benzyloxy)-2-ethynylpyrrolidin-1-yl)-3-oxopropanenitrile (**14**) (832 mg, 3.10 mmol, 1.0 equiv) and a stir bar in a nitrogen atmosphere glove box was added acetonitrilebis[2-diphenylphosphino-6-*t*-butylpyridine]cyclopentadienylruthenium(II) hexafluorophosphate (**15**) (248 mg, 0.250 mmol, 0.08 equiv). A deoxygenated by via three cycles of freeze/pump/thaw method) solutiong of acetone (6.3 mL) and HPLC grade water (0.280 mL) was added to the Schlenk flask in the glove box via syringe. The reaction vessel was then capped and removed from the glove box and the resulting yellow solution was placed in a preheated oil bath and stirred at 70 °C for 24 h, at which time the reaction mixture was diluted with ethyl acetate (10 mL) and concentrated *in vacuo*. The resulting yellow oil was purified by silica gel chromatography (40 mL SiO_2 with 1:1 to 2:1 ethyl acetate:hexanes) to yield **16** (770 mg, 2.87 mmol, 93%) as a yellow solid. M.P. 94-96 °C; TLC (ethyl acetate:hexanes, 4:1 v/v): R_f=0.36; **^{1}H NMR** (600 MHz, $CDCl_3$) δ = 7.37 – 7.27 (m, 6H), 4.66 (d, J = 11.9 Hz, 1H), 4.46 (d, J = 11.9 Hz, 1H), 4.11 (t, J = 3.6 Hz, 1H), 3.86 (dt, J = 13.9, 4.5 Hz, 1H), 3.76 – 3.70 (m, 1H), 3.57 (td, J = 11.4, 7.3 Hz, 1H), 2.93 (ddd, J = 18.9, 14.0, 2.4 Hz, 1H), 2.47 (ddd, J = 18.9, 6.6, 5.0 Hz, 1H), 2.28 (dd, J = 13.9, 7.2 Hz, 1H), 1.87 (dtd, J = 13.8, 10.0, 3.7 Hz, 1H); **^{13}C NMR** (150 MHz, $CDCl_3$) δ = 157.3, 153.3, 137.6, 128.6 (2C), 128.0, 127.6 (2C), 114.8, 113.6, 78.0, 71.1, 59.6,

[5] D. B. Grotjahn, D. A. Lev, *J. Am. Chem. Soc.,* 2004, **126**, 12232.

42.9, 28.1, 25.4; **IR** (NaCl, thin film) ν_{max}: 3032, 2952, 2233, 1665, 1608, 1453, 1346, 1302, 1210 cm^{-1}; **HRMS** (ESI) calcd for $C_{16}H_{17}O_2N_2$ ($[M+H]^+$): 269.1285, found 269.1282.

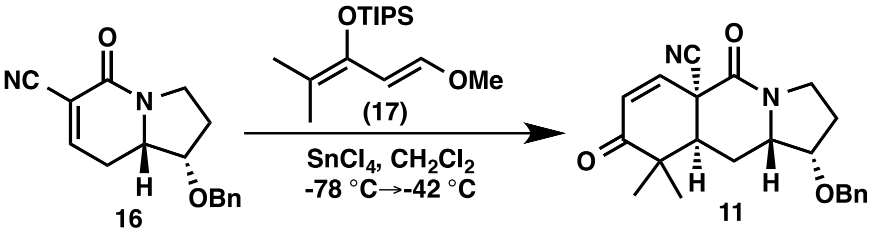

A solution of (1*S*,8a*S*)-1-(benzyloxy)-5-oxo-1,2,3,5,8,8a-hexahydroindolizine-6-carbonitrile (**16**) (2.82 g, 10.52 mmol, 1.0 equiv) and (*E*)-triisopropyl((1-methoxy-4-methylpenta-1,3-dien-3-yl)oxy)silane (**17**)[6] (5.97 g, 21.0 mmol, 2.0 equiv) in CH_2Cl_2 (105 mL, 0.1M) was cooled to –78 °C and then $SnCl_4$ (1.0 M in CH_2Cl_2, 12.6 mL, 12.62 mmol, 1.2 equiv) was added dropwise. The resulting red solution was then warmed to –42 °C (MeCN/dry ice) and after 40 min, additional (*E*)-triisopropyl((1-methoxy-4-methylpenta-1,3-dien-3-yl)oxy)silane (**17**) (2.0 g, 7.03 mmol, 0.66 equiv) was added and the MeCN/dry ice bath removed. The solution was allowed to warm to room temperature and stirred for 30 min, then saturated aqueous $NaHCO_3$ (100 mL) was added and the mixture was stirred vigorously for 3 h. The resulting mixture was vacuum filtered through a fritted funnel and the layers separated. The aqueous layer was extracted with CH_2Cl_2 (3 x 100 mL), and the combined organic extracts were dried over Na_2SO_4, filtered, and concentrated *in vacuo*. The resulting oil was purified by silica gel chromatography (200 mL SiO_2 with 3:7 to 7:3 ethyl acetate:hexanes) to yield **11** (2.86 g, 7.85 mmol, 75%) as a yellow foam. TLC (ethyl acetate:hexanes, 2:1 v/v): R_f=0.30; **^{1}H NMR** (600 MHz, $CDCl_3$) δ = 7.38 – 7.27 (m, 5H), 6.81 (d, *J* = 10.1 Hz, 1H), 6.17 (d, *J* = 10.1 Hz, 1H), 4.66 (d, *J* = 12.2 Hz, 1H), 4.44 (d, *J* = 12.2 Hz, 1H), 3.98 (t, *J* = 3.7 Hz, 1H), 3.81 (ddd, *J* = 12.1, 9.8, 8.0 Hz, 1H), 3.58 – 3.52 (m, 1H), 3.48 (ddd, *J* = 12.1, 9.6, 2.2 Hz, 1H), 2.81 (dd, *J* = 7.5, 3.6 Hz, 1H), 2.53 (ddd, *J* = 14.9, 10.8, 7.5 Hz, 1H), 2.23 – 2.16 (m, 1H), 1.97 – 1.90 (m, 1H), 1.82 (dtd, *J* = 13.8, 9.8, 4.0 Hz, 1H), 1.39 (s, 3H), 1.15 (s, 3H); **^{13}C NMR** (150 MHz, $CDCl_3$) δ = 200.8, 161.8, 138.8, 137.7, 129.6, 128.6 (2C), 128.0, 127.5 (2C), 118.2, 78.2, 70.9, 59.6, 45.6, 45.3, 45.1, 44.3, 28.0, 24.8,

[6] E. V. Mercado-Marin, P. Garcia-Reynaga, S. Romminger, E. F. Pimenta, D. K. Romney, M. W. Lodewyk, D. E. Williams, R. J. Andersen, S. J. Miller, D. J. Tantillo, R. G. S. Berlinck, R. Sarpong, *Nature,* 2014, **509**, 318.

22.6, 22.4; **IR** (NaCl, thin film) ν_{max}: 2948, 2868, 2250, 1660, 1589, 1454, 1392, 1345 cm^{-1}; **HRMS** (ESI) calcd for $C_{22}H_{24}O_3N_2Na$ ($[M+Na]^+$): 387.1679, found 387.1676.

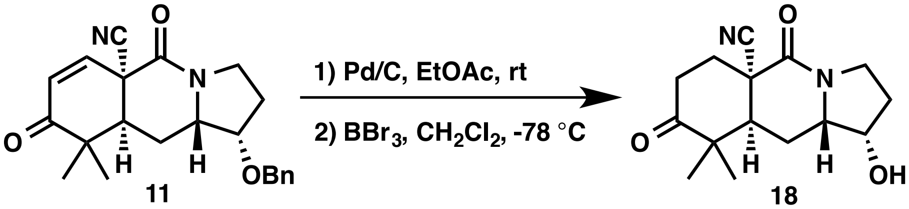

11 18

To a round-bottomed flask containing (1*S*,5a*R*,9a*S*,10a*S*)-1-(benzyloxy)-9,9-dimethyl-5,8-dioxo-1,2,3,8,9,9a,10,10a-octahydropyrrolo[1,2-*b*]isoquinoline-5a(5*H*)-carbonitrile (**11**) (930 mg, 2.55 mmol, 1.0 equiv) was added Pd/C (93 mg, 10 wt%) and ethyl acetate (2 mL) and the atmosphere purged with H_2 (three cycles of evacuation/backfill). Additional ethyl acetate (45 mL, 0.6M) was added and the resulting mixture was stirred at room temperature overnight. After 16 h, the reaction mixture was filtered through Celite and washed with ethyl acetate. The solvent was removed *in vacuo* and the resulting pale yellow oil was used without further purification. The crude oil was dissolved in CH_2Cl_2 (78 mL, 0.033M) and cooled to –78 °C. BBr_3 (1.10 mL, 11.7 mmol, 4.6 equiv) was then added dropwise along the side of the flask and stirred for 15 min at which point saturated aqueous $NaHCO_3$ (80 mL) was added and the mixture warmed to room temperature. The layers were separated and the aqueous layer was extracted with CH_2Cl_2 (4 x 80 mL). The combined organic extracts were dried over Na_2SO_4, filtered, and concentrated *in vacuo*. The resulting oil was purified by silica gel chromatography (40 mL SiO_2 with 2% to 5% methanol:dichloromethane) to yield **18** (524 mg, 1.89 mmol, 74% over 2 steps) as a white foam. TLC (methanol:dichloromethane, 1:9 v/v): R_f=0.60; **^{1}H NMR** (600 MHz, $CDCl_3$) δ = 4.24 – 4.14 (m, 1H), 3.93 (dt, *J* = 12.5, 9.0 Hz, 1H), 3.56 – 3.49 (m, 1H), 3.48 – 3.41 (m, 1H), 3.34 (ddd, *J* = 12.8, 7.7, 5.2 Hz, 1H), 2.73 – 2.63 (m, 2H), 2.60 – 2.36 (m, 4H), 2.00 (td, *J* = 8.5, 8.1, 3.3 Hz, 2H), 1.91 (dt, *J* = 14.7, 6.3 Hz, 1H), 1.37 (s, 3H), 1.12 (s, 3H); **^{13}C NMR** (150 MHz, $CDCl_3$) δ = 212.3, 164.2, 121.5, 72.3, 60.1, 48.0, 46.1, 43.9, 43.0, 34.3, 31.4, 29.8, 26.3, 22.8, 21.9; **IR** (NaCl, thin film) ν_{max}: 3413, 2927, 2360, 1712, 1647, 1456 cm^{-1}; **HRMS** (ESI) calcd for $C_{15}H_{20}O_3N_2Na$ ($[M+Na]^+$): 299.1366, found 299.1367.

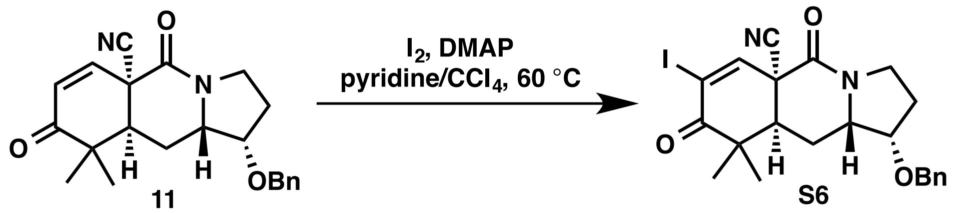

Iodine (2.10 g, 8.24 mmol, 1.5 equiv) and 4-dimethylaminopyridine (1.00g, 8.24 mmol, 1.5 equiv) were added sequentially to a solution of (1*S*,5a*R*,9a*S*,10a*S*)-1-(benzyloxy)-9,9-dimethyl-5,8-dioxo-1,2,3,8,9,9a,10,10a-octahydropyrrolo[1,2-*b*]isoquinoline-5a(5*H*)-carbonitrile (**11**) (2.00 g, 5.50 mmol, 1.0 equiv) in a mixture of pyridine/CCl_4 (1:1 v/v,14.0 mL, 0.40M). The reaction flask was wrapped in aluminum foil and the resulting dark brown mixture was stirred at 60 °C in the dark for 15 h and then the reaction cooled to room temperature. Additional iodine (2.10 g, 8.24 mmol, 1.5 equiv) and 4-dimethylaminopyridine (1.00g, 8.24 mmol, 1.5 equiv) were added sequentially to the solution, which was then stirred at 60 °C for 6 h, at which time the reaction mixture was cooled to room temperature. The reaction mixture was then poured into saturated aqueous $Na_2S_2O_3$ (80 mL) and the aqueous layer was extracted with a mixture of ethyl acetate/hexanes (1:1 v/v, 4 x 80 mL). The combined organic extracts were dried over Na_2SO_4, filtered, and concentrated *in vacuo*. The resulting dark oil was purified by silica gel chromatography (50 mL SiO_2 with 1:4 to 3:2 ethyl acetate:hexanes) to yield **S6** (2.08 g, 4.24 mmol, 77%) as a beige foam. TLC (ethyl acetate:hexanes, 4:1 v/v): R_f= 0.65; **^{1}H NMR** (600 MHz, $CDCl_3$) δ = 7.55 (s, 1H), 7.37 – 7.27 (m, 5H), 4.65 (d, *J* = 12.1 Hz, 1H), 4.43 (d, *J* = 12.2 Hz, 1H), 3.99 (t, *J* = 3.8 Hz, 1H), 3.84 – 3.77 (m, 1H), 3.53 – 3.45 (m, 2H), 2.85 (dd, *J* = 7.4, 3.7 Hz, 1H), 2.52 (ddd, *J* = 15.0, 10.7, 7.4 Hz, 1H), 2.20 (ddd, *J* = 14.1, 8.1, 2.3 Hz, 1H), 1.91 (dt, *J* = 15.0, 4.5 Hz, 1H), 1.88 – 1.80 (m, 1H), 1.44 (s, 3H), 1.19 (s, 3H); **^{13}C NMR** (150 MHz, $CDCl_3$) δ = 194.8, 160.6, 145.5, 137.6, 128.6 (2C), 128.0, 127.4 (2C), 117.1, 105.6, 78.2, 70.9, 59.5, 48.2, 45.8, 45.1, 44.4, 27.9, 25.2, 22.9, 22.5; **IR** (neat) ν_{max}: 3032, 2921, 1697, 1660, 1448, 1346, 1204 cm^{-1}; **HRMS** (ESI) calcd for $C_{22}H_{23}O_3N_2INa$ ($[M+Na]^+$): 513.0646, found 513.0670.

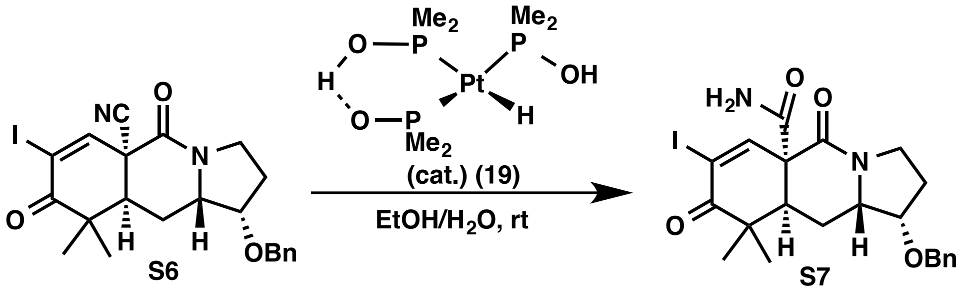

$(Me_2POH)_2Pt(H)(Me_2PO)$ (**19**)[7] (88 mg, 0.20 mmol, 0.2 equiv) was added in one portion to a solution of (1*S*,5a*S*,9a*S*,10a*S*)-1-(benzyloxy)-7-iodo-9,9-dimethyl-5,8-dioxo-1,2,3,8,9,9a,10,10a-octahydropyrrolo[1,2-*b*]isoquinoline-5a(5*H*)-carbonitrile (**S6**) (500 mg, 1.02 mmol, 1.0 equiv) in a mixture of $EtOH/H_2O$ (4:1 v/v, 5.1 mL, 0.2M). The reaction vessel was wrapped in aluminum foil and the resulting mixture stirred in the absence of light at room temperature. After 62 h, additional $(Me_2POH)_2Pt(H)(Me_2PO)$ (**19**) (44 mg, 0.10 mmol, 0.1 equiv) was added and the mixture stirred in the absence for light at room temperature for an additional 36 h. The resulting solution was then diluted with CH_2Cl_2 (10 mL) and passed through a short column containing silica gel (20 mL) layered with Na_2SO_4 (20 mL) and washed with 10% $MeOH/CH_2Cl_2$ (50 mL). The filtrate was concentrated *in vacuo* and purified by silica gel chromatography (20 mL SiO_2 with 2% to 5% methanol:dichloromethane) to yield **S7** (423 mg, 0.832 mmol, 82%) as a yellow foam. TLC (methanol:dichloromethane, 1:19 v/v): R_f= 0.33; **^{1}H NMR** (600 MHz, $CDCl_3$) δ = 7.84 (s, 1H), 7.39 – 7.26 (m, 5H), 7.25 (s, 1H), 5.30 (s, 1H), 4.60 (d, *J* = 12.0 Hz, 1H), 4.45 (d, *J* = 12.0 Hz, 1H), 3.94 (t, *J* = 3.3 Hz, 1H), 3.86 (dt, *J* = 12.6, 9.1 Hz, 1H), 3.72 (ddd, *J* = 12.0, 5.4, 2.8 Hz, 1H), 3.45 (ddd, *J* = 12.6, 10.3, 2.2 Hz, 1H), 3.24 (dd, *J* = 5.4, 2.8 Hz, 1H), 2.35 (ddd, *J* = 14.9, 11.9, 5.4 Hz, 1H), 2.23 (ddd, *J* = 14.0, 8.6, 2.2 Hz, 1H), 2.07 – 2.01 (m, 1H), 1.91 – 1.83 (m, 1H), 1.34 (s, 3H), 1.16 (s, 3H); **^{13}C NMR** (150 MHz, $CDCl_3$) δ = 196.0, 168.0, 167.3, 152.6, 137.7, 128.6 (2C), 128.0, 127.5 (2C), 103.0, 77.7, 70.8, 60.9, 59.1, 45.2, 43.7, 39.4, 27.9, 23.3, 22.6, 20.7; **IR** (neat) ν_{max}: 3416, 3323, 2970, 2921, 1691, 1636, 1584, 1455, 1344, 1205 cm^{-1}; **HRMS** (ESI) calcd for $C_{22}H_{25}O_4N_2INa$ ($[M+Na]^+$): 531.0751, found 531.0739.

[7] T. Ghaffar, A. W. Parkins, *Tetrahedron Lett.,* 1995, **36**, 8657.

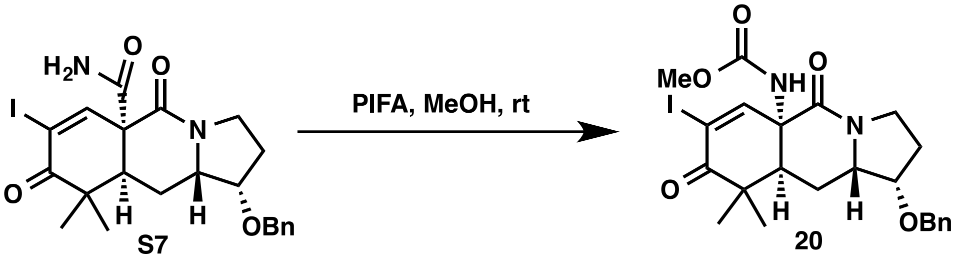

To a solution of (1*S*,5a*S*,9a*S*,10a*S*)-1-(benzyloxy)-7-iodo-9,9-dimethyl-5,8-dioxo-1,2,3,8,9,9a,10,10a-octahydropyrrolo[1,2-*b*]isoquinoline-5a(5*H*)-carboxamide (**S7**) (700 mg, 1.38 mmol, 1.0 equiv) in methanol (14.0 mL, 0.1M) cooled to 0 °C was added [Bis(trifluoroacetoxy)iodo]benzene (PIFA) (650 mg, 1.51 mmol, 1.1 equiv) in one portion. The resulting mixture was stirred at room temperature for 16 h then poured into saturated aqueous $NaHCO_3$ (60 mL) and the aqueous layer extracted with ethyl acetate (4 x 60 mL). The combined organic extracts were dried over Na_2SO_4, filtered, and concentrated *in vacuo*. The resulting orange oil was purified by silica gel chromatography (40 mL SiO_2 with 2:3 to 4:1 ethyl acetate:hexanes) to yield **20** (658 mg, 1.22 mmol, 89%) as a pale yellow foam. TLC (ethyl acetate:hexanes, 4:1 v/v): R_f= 0.39; **^{1}H NMR** (600 MHz, $CDCl_3$) δ = 7.41 (s, 1H), 7.35 – 7.26 (m, 5H), 5.63 (bs, 1H), 4.61 (d, *J* = 12.0 Hz, 1H), 4.42 (d, *J* = 12.0 Hz, 1H), 3.95 (td, *J* = 3.8, 1.1 Hz, 1H), 3.78 – 3.71 (m, 1H), 3.64 (s, 3H), 3.45 – 3.38 (m, 2H), 3.11 (s, 1H), 2.39 (ddd, *J* = 14.7, 11.7, 7.7 Hz, 1H), 2.15 (ddt, *J* = 13.9, 7.5, 1.7 Hz, 1H), 1.90 (ddd, *J* = 14.8, 4.4, 2.0 Hz, 1H), 1.82 – 1.75 (m, 1H), 1.36 (s, 3H), 1.19 (s, 3H); **^{13}C NMR** (150 MHz, $CDCl_3$) δ = 197.0, 166.3, 155.8, 152.0, 137.7, 128.6 (2C), 128.0, 127.5 (2C), 104.0, 78.7, 71.1, 61.7, 59.5, 52.5, 45.5, 44.4, 44.2, 28.3, 27.4, 23.6, 22.4; **IR** (neat) ν_{max}: 3279, 3031, 2949, 1724, 1692, 1654, 1526, 1454, 1346, 1260 cm^{-1}; **HRMS** (ESI) calcd for $C_{23}H_{28}O_5N_2I$ ($[M+H]^+$): 539.1038, found 539.1049.

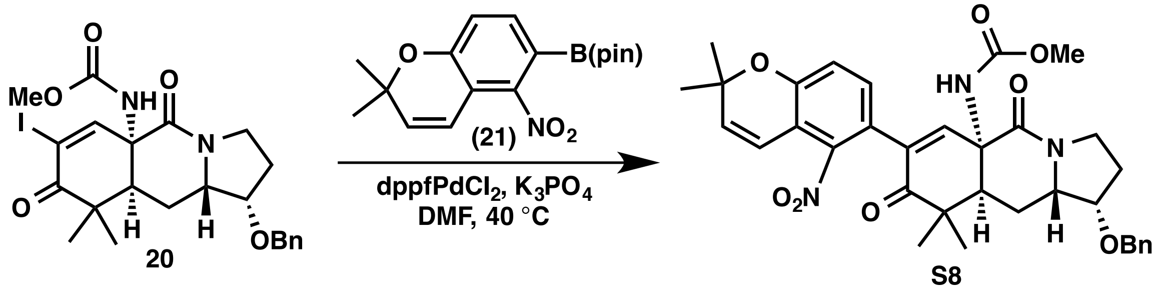

A round-bottomed flask was charged with methyl ((1*S*,5a*R*,9a*S*,10a*S*)-1-(benzyloxy)-7-iodo-9,9-dimethyl-5,8-dioxo-1,2,3,8,9,9a,10,10a-octahydropyrrolo[1,2-*b*]isoquinolin-5a(5*H*)-yl)carbamate (**20**) (670 mg, 1.24 mmol, 1.0 equiv), 2-(2,2-dimethyl-5-nitro-2*H*-chromen-6-yl)-4,4,5,5-

tetramethyl-1,3,2-dioxaborolane (**21**)[8] (620 mg, 1.87 mmol, 1.50 equiv), dppfPdCl$_2$ (101 mg, 0.124 mmol, 0.10 equiv) and K$_3$PO$_4$ (987 mg, 4.65 mmol, 3.75 equiv). *N,N*-dimethylformamide (12.4 mL, 0.1M) was added and the head space was deoxygenated with N$_2$ (three cycles of evacuation/backfill). The resulting brown mixture was stirred at 40 ºC. After 16 h, the reaction mixture was cooled to room temperature, poured into saturated aqueous NH$_4$Cl (100 mL) and the aqueous layer extracted with EtOAc (4 x 100 mL). The combined organic extracts were dried with Na$_2$SO$_4$, filtered, and concentrated *in vacuo*. The resulting dark oil was purified by silica gel chromatography (40 mL SiO$_2$ with 2:3 to 4:1 ethyl acetate:hexanes) to yield **S8** (715 mg, 1.16 mmol, 94%) as a dark brown foam. TLC (ethyl acetate:hexanes, 3:2 v/v): R_f= 0.23; **^{1}H NMR** (600 MHz, CDCl$_3$) δ = 7.37 – 7.27 (m, 5H), 6.95 (d, *J* = 8.3 Hz, 1H), 6.92 (d, *J* = 8.3 Hz, 1H), 6.62 (s, 1H), 6.40 (d, *J* = 10.3 Hz, 1H), 5.81 (d, *J* = 10.3 Hz, 1H), 5.29 (bs, 1H), 4.62 (d, *J* = 12.0 Hz, 1H), 4.44 (d, *J* = 12.0 Hz, 1H), 3.99 (t, *J* = 3.8 Hz, 1H), 3.79 (td, *J* = 11.3, 7.4 Hz, 1H), 3.69 (s, 3H), 3.53 – 3.47 (m, 2H), 3.02 (d, *J* = 7.8 Hz, 1H), 2.38 (ddd, *J* = 14.6, 11.6, 8.0 Hz, 1H), 2.18 (dd, *J* = 13.7, 7.2 Hz, 1H), 1.99 – 1.93 (m, 1H), 1.90 – 1.82 (m, 1H), 1.46 (s, 6H), 1.39 (s, 3H), 1.20 (s, 3H); **^{13}C NMR** (150 MHz, CDCl$_3$) δ = 200.9, 167.3, 156.4, 154.0, 146.8, 141.6, 137.9, 136.1, 134.2, 131.2, 128.5 (2C), 127.8, 127.5 (2C), 122.0, 119.5, 116.7, 114.6, 79.2, 76.8, 71.1, 59.5, 59.1, 52.3, 45.4, 45.0, 44.2, 28.5, 28.3, 28.0, 27.8, 23.5, 22.9; **IR** (neat) ν_{max}: 3289, 2977, 1722, 1654, 1527, 1456, 1353, 1278, 1205, 1119 cm^{-1}; **HRMS** (ESI) calcd for C$_{34}$H$_{37}$O$_8$N$_3$Na ([M+Na]$^+$): 638.2473, found 638.2473.

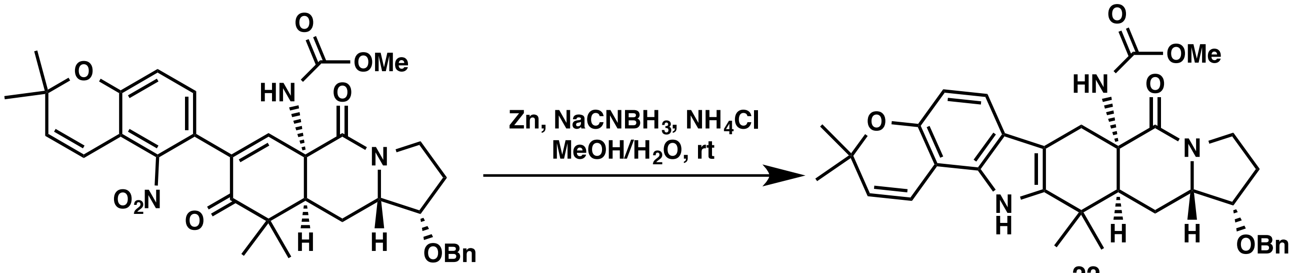

S8 **22**

This procedure was adapted from a known procedure.[9] To a solution of methyl ((1*S*,5a*S*,9a*S*,10a*S*)-1-(benzyloxy)-7-(2,2-dimethyl-5-nitro-2*H*-chromen-6-yl)-9,9-dimethyl-5,8-dioxo-1,2,3,8,9,9a,10,10a-octahydropyrrolo[1,2-*b*]isoquinolin-5a(5*H*)-yl)carbamate (**S8**) (110 mg, 0.179 mmol, 1.0 equiv) and saturated aqueous NH$_4$Cl (4.7 mL) in methanol (14.3 mL,

[8] S. B. Herzon, A. G. Myers, *J. Am. Chem. Soc.,* 2005, **127**, 5342.
[9] T. C. Turner, K. Shibayama, D. L. Boger, *Org. Lett.,* 2013, **15**, 1100.

0.0125M) was added $NaCNBH_3$ (56 mg, 0.895 mmol, 5.0 equiv) followed by zinc dust[10] (89 mg, 1.43 mmol, 8.0 equiv). The resulting solution was then stirred at room temperature and zinc dust (267 mg, 4.29 mmol, 24 equiv) was added in three portions (3 x 89 mg) at 30 minute intervals. Two hours after the last addition, the reaction mixture was filtered using a Büchner funnel to remove the solids and to the resulting filtrate was added saturated aqueous $NaHCO_3$ (60 mL). The aqueous layer was extracted with ethyl acetate (3 x 60 mL) and the combined organic extracts were dried over Na_2SO_4, filtered, and concentrated *in vacuo*. The resulting beige powder was purified by silica gel chromatography (60 mL SiO_2 with 1% to 2% to 5% methanol:dichloromethane) to yield **22** (72 mg, 0.126 mmol, 71%) as an off white powder. TLC (methanol:dichloromethane, 1:19 v/v): R_f = 0.24; **^{1}H NMR** (600 MHz, $CDCl_3$) δ = 7.68 (s, 1H), 7.42 – 7.33 (m, 4H), 7.31 – 7.27 (m, 1H), 7.11 (d, *J* = 8.4 Hz, 1H), 6.65 (d, *J* = 8.4 Hz, 1H), 6.60 (d, *J* = 9.6 Hz, 1H), 5.68 (d, *J* = 9.6 Hz, 1H), 4.87 (s, 1H), 4.77 (d, *J* = 12.6 Hz, 1H), 4.41 (d, *J* = 12.6 Hz, 1H), 4.06 – 3.98 (m, 1H), 3.89 – 3.85 (m, 1H), 3.62 (s, 3H), 3.54 – 3.49 (m, 1H), 3.42 – 3.34 (m, 1H), 3.26 (dd, *J* = 13.3, 6.0 Hz, 1H), 3.03 (d, *J* = 16.5 Hz, 1H), 2.79 (d, *J* = 16.5 Hz, 1H), 2.37 – 2.30 (m, 1H), 2.21 – 2.14 (m, 1H), 1.90 – 1.80 (m, 1H), 1.76 – 1.68 (m, 1H), 1.47 (s, 3H), 1.46 (s, 6H), 1.33 (s, 3H); **^{13}C NMR** (150 MHz, $CDCl_3$) δ = 171.0, 156.2, 148.8, 138.8, 138.6, 132.8, 130.0, 128.5, 127.6, 122.0, 117.8, 117.3, 110.4, 105.3, 102.9, 80.0, 75.7, 70.5, 59.4, 57.8, 52.0, 43.3, 40.8, 34.8, 30.1, 28.7, 28.10, 27.56, 27.55, 27.4, 22.4; **IR** (neat) ν_{max}: 3434, 3307, 2972, 2923, 1715, 1638, 1502, 1455, 1356, 1253 cm^{-1}; **HRMS** (ESI) calcd for $C_{34}H_{39}O_5N_3Na$ ($[M+Na]^+$): 592.2782, found 592.2788.

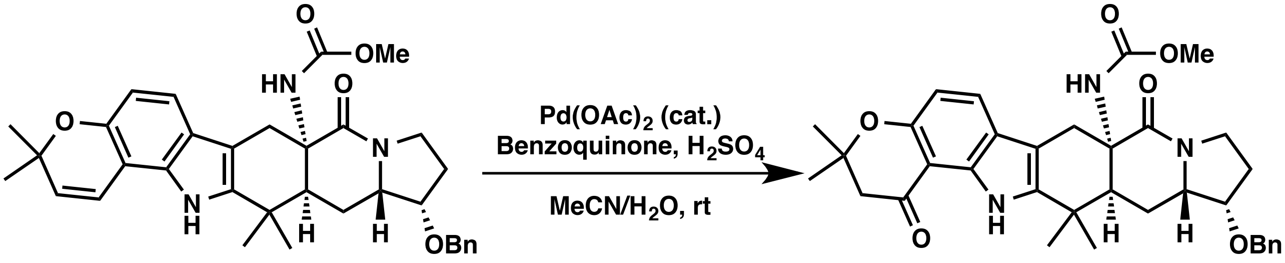

22 **S9**

A 20 mL vial was charged with $Pd(OAc)_2$ (13.4 mg, 0.060 mmol, 0.40 equiv) and *p*-benzoquinone (22.4 mg, 0.224 mmol, 1.50 equiv). The vial was fitted with a septum, purged

[10] Note: Zinc dust was freshly activated by sequential washing with 0.1 M aq. HCl (3 x 10 mL) and H_2O (10 mL). The solid was collected by filtration and washed with EtOH (2 x 10 mL) and Et_2O (2 x 10 mL), then dried under high vacuum to give a soft blue-gray solid, which was ground to a powder.

with N_2 (three cycles of evacuation/backfill) and then MeCN (3.6 mL) and H_2O (1.06 mL) were added via syringe to give an orange solution. H_2SO_4 (11.4 µL, 95% wt in H_2O) was then added via syringe and the resulting pale yellow solution was stirred at room temperature for 5 min. In a separate vial, methyl ((7a*S*,12*S*,12a*S*,13a*S*)-12-(benzyloxy)-3,3,14,14-tetramethyl-8-oxo-3,7,10,11,12,12a,13,13a,14,15-decahydroindolizino[6,7-*h*]pyrano[3,2-*a*]carbazol-7a(8*H*)-yl)carbamate (**22**) (85 mg, 0.149 mmol, 1.0 equiv) was dissolved in MeCN (3.6 mL, 0.041M) under an N_2 atmosphere. To this was added, drop-wise, the solution of the catalyst as described above and the resulting dark red mixture was stirred at room temperature. After 17 h, the resulting dark brown reaction mixture was poured into saturated aqueous $NaHCO_3$ (50 mL) and the aqueous layer extracted with EtOAc (3 x 50 mL). The combined organic extracts were dried over Na_2SO_4, filtered and concentrated *in vacuo*. The resulting red oil residue was purified by silica gel chromatography (30 mL SiO_2 with 1% to 2% to 3% methanol:dichloromethane) to yield **S9** (67 mg, 0.114 mmol, 77%) as a yellow foam. TLC (methanol:dichloromethane, 1:19 v/v): R_f= 0.33; **^{1}H NMR** (600 MHz, $CDCl_3$) δ = 9.74 (s, 1H), 7.46 (d, *J* = 8.4 Hz, 1H), 7.41 – 7.31 (m, 4H), 7.30 – 7.26 (m, 1H), 6.63 (d, *J* = 8.4 Hz, 1H), 4.89 (bs, 1H), 4.75 (d, *J* = 12.5 Hz, 1H), 4.39 (d, *J* = 12.5 Hz, 1H), 4.00 (dt, *J* = 12.1, 8.5 Hz, 1H), 3.85 (t, *J* = 3.9 Hz, 1H), 3.60 (s, 3H), 3.53 – 3.45 (m, 1H), 3.38 – 3.31 (m, 1H), 3.26 (dd, *J* = 13.1, 6.1 Hz, 1H), 3.02 (d, *J* = 16.6 Hz, 1H), 2.82 (d, *J* = 16.6 Hz, 1H), 2.73 (s, 2H), 2.36 – 2.29 (m, 1H), 2.19 – 2.10 (m, 1H), 1.88 – 1.78 (m, 1H), 1.73 – 1.63 (m, 1H), 1.49 – 1.44 (m, 9H), 1.34 (s, 3H); **^{13}C NMR** (150 MHz, $CDCl_3$) δ = 194.1, 170.8, 157.4, 156.1, 139.8, 138.5, 134.0, 128.4, 127.6, 127.5, 126.8, 121.6, 109.9, 105.3, 102.7, 79.9, 79.5, 70.4, 59.3, 57.7, 51.9, 48.8, 43.1, 40.6, 34.7, 29.90, 28.3, 27.9, 27.5, 26.7, 26.6, 22.3; **IR** (neat) ν_{max}: 3442, 3357, 2974, 2950 1732, 1709, 1653, 1618, 1580, 1457, 1369 cm^{-1}; **HRMS** (ESI) calcd for $C_{34}H_{40}O_6N_3$ ($[M+H]^+$): 586.2912, found 586.2918; calcd for $C_{34}H_{39}O_6N_3Na$ ($[M+Na]^+$): 608.2731, found 608.2726.

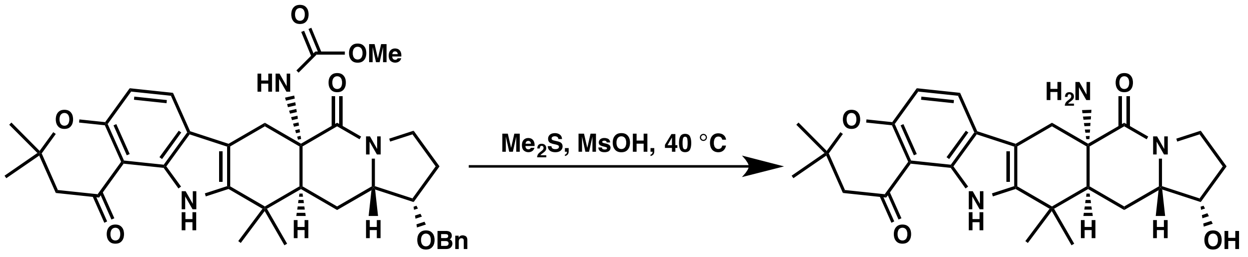

S9 23

Dimethylsulfide (0.310 mL, 4.27 mmol, 20.0 equiv) was added to a solution of methyl ((7a*S*,12*S*,12a*S*,13a*S*)-12-(benzyloxy)-3,3,14,14-tetramethyl-1,8-dioxo-1,2,3,7,10,11,12,12a,13,13a,14,15-dodecahydroindolizino[6,7-*h*]pyrano[3,2-*a*]carbazol-7a(8*H*)-yl)carbamate (**S9**) (125 mg, 0.214 mmol, 1.0 equiv) in MsOH (4.3 mL, 0.05M) at room temperature. The reaction mixture was stirred at 40 ºC for 15 h and then cooled to room temperature. The resulting dark red reaction mixture was added dropwise to a stirring solution of saturated aqueous K_2CO_3 (50 mL) at 0 ºC. Stirring was continued until bubbling had ceased and then the aqueous solution was transferred to a separatory funnel and extracted with ethyl acetate (4 x 50 mL). The combined organic extracts were dried over Na_2SO_4, filtered, and concentrated *in vacuo*. The resulting yellow oil was purified by silica gel chromatography (20 mL SiO_2 with 2% to 20% methanol:dichloromethane) to yield **23** (87 mg, 0.199 mmol, 93%) as a yellow foam. TLC (methanol:dichloromethane, 1:9 v/v): R_f= 0.03; **^{1}H NMR** (600 MHz, $CDCl_3$) δ = 9.72 (s, 1H), 7.51 (d, *J* = 8.5 Hz, 1H), 6.63 (d, *J* = 8.5 Hz, 1H), 4.12 (t, *J* = 2.8 Hz, 1H), 3.97 (dt, *J* = 12.8, 8.7 Hz, 1H), 3.53 – 3.44 (m, 1H), 3.30 (dt, *J* = 13.1, 7.4 Hz, 1H), 3.03 (d, *J* = 15.8 Hz, 1H), 2.96 (bs, 2H), 2.75 (s, 2H), 2.70 (d, *J* = 15.8 Hz, 1H), 2.40 (ddd, *J* = 14.4, 6.4, 3.1 Hz, 1H), 2.18 (dd, *J* = 11.8, 6.2 Hz, 1H), 2.04 – 1.95 (m, 2H), 1.74 (ddd, *J* = 14.1, 11.8, 9.8 Hz, 1H), 1.61 (s, 3H), 1.49 (s, 6H), 1.41 (s, 3H); **^{13}C NMR** (150 MHz, $CDCl_3$) δ = 194.3, 174.0, 157.6, 139.1, 134.3, 127.3, 122.1, 109.8, 105.4, 104.5, 79.6, 73.5, 59.0, 57.2, 48.9, 45.6, 42.9, 35.1, 31.7, 31.0, 30.4, 28.0, 26.8, 26.7, 22.1; **IR** (neat) ν_{max}: 3440, 3395, 3364, 2976, 2934, 1620, 1584, 1463, 1370 cm^{-1}; **HRMS** (ESI) calcd for $C_{25}H_{31}O_4N_3$ ($[M+H]^+$): 438.2387, found 438.2384.

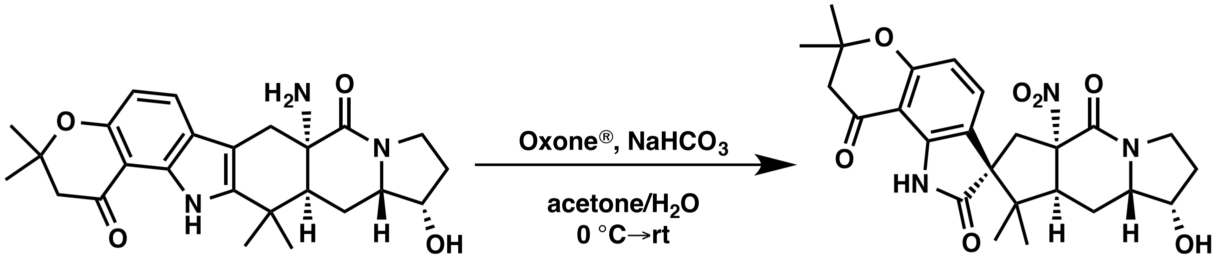

23 24

A saturated aqueous solution of $NaHCO_3$ (1.5 mL) was added to a solution of (7a*S*,12*S*,12a*S*,13a*S*)-7a-amino-12-hydroxy-3,3,14,14-tetramethyl-2,3,7,7a,10,11,12,12a,13,13a,14,15-dodecahydroindolizino[6,7-*h*]pyrano[3,2-*a*]carbazole-1,8-dione (**23**) (20.0 mg, 0.046 mmol, 1.0 equiv) in acetone (2.0 mL, 0.023M) at 0 ºC, resulting in precipitate formation. A solution of Oxone® (80 mg, 0.526 mmol, 11.4 equiv) in deionized water (1.00 mL, 0.53M) was added drop-wise and the mixture was warmed to room temperature by allowing the ice bath to expire. After 2 h, the resulting mixture was diluted with deionized water (3.0 mL) and extracted with ethyl acetate (4 x 5.0 mL). The combined organic extracts were dried with Na_2SO_4, filtered, and concentrated *in vacuo*. The resulting beige powder was purified by silica gel chromatography (10 mL SiO_2 with 1% to 2% to 3% to 5% methanol:dichloromethane) to yield **24** (11.1 mg, 0.023 mmol, 50%) as a white powder. TLC (methanol:dichloromethane, 1:19 v/v): R_f= 0.29; **^{1}H NMR** (600 MHz, $(CD_3)_2SO$) δ = 10.21 (s, 1H), 7.46 (d, *J* = 8.4 Hz, 1H), 6.52 (d, *J* = 8.4 Hz, 1H), 5.07 (d, *J* = 4.1 Hz, 1H), 4.15 (q, *J* = 3.6 Hz, 1H), 3.74 (d, *J* = 11.9 Hz, 1H), 3.62 (d, *J* = 7.6 Hz, 1H), 3.59 – 3.51 (m, 1H), 3.41 – 3.35 (m, 1H), 3.18 (d, *J* = 16.1 Hz, 1H), 2.83 – 2.74 (m, 3H), 2.46 (dd, *J* = 13.2, 6.9 Hz, 1H), 1.98 – 1.88 (m, 2H), 1.84 (dd, *J* = 13.3, 7.9 Hz, 1H), 1.40 (s, 3H), 1.39 (s, 3H), 1.11 (s, 3H), 0.77 (s, 3H); **^{13}C NMR** (150 MHz, $(CD_3)_2SO$) δ = 192.6, 181.0, 162.6, 158.8, 142.7, 133.0, 118.7, 109.0, 104.9, 94.2, 79.2, 70.6, 61.1, 58.6, 49.8, 49.0, 48.0, 43.9, 40.1, 30.9, 26.3, 26.0, 22.7, 21.6, 19.0; **IR** (neat) ν_{max}: 3459, 3404, 2966, 2938, 1717, 1673, 1642, 1625, 1549, 1465, 1370, 1348, 1322, 1255 cm^{-1}; **HRMS** (ESI) calcd for $C_{25}H_{30}O_7N_3$ ($[M+H]^+$): 484.2078, found 484.2096.

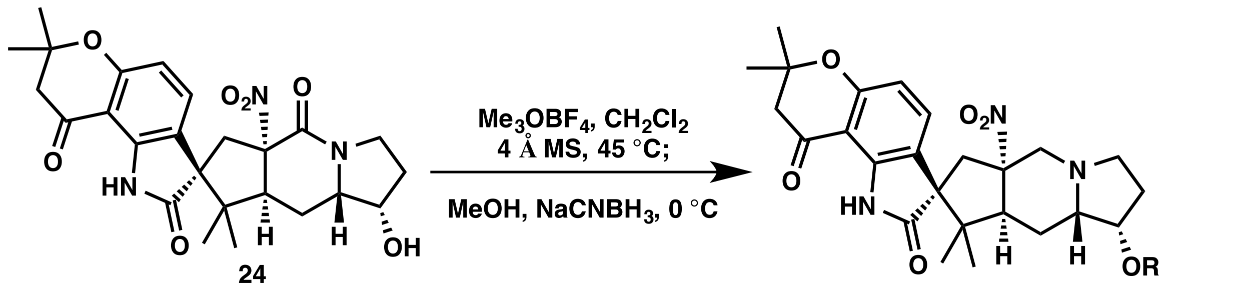

24

***ent*-17-hydroxycitrinalin B (7), R = H**
methyl ether (7b), R = Me

To a Schlenk tube charged with (1*S*,5a*S*,7*R*,8a*S*,9a*S*)-1-hydroxy-7',7',8,8-tetramethyl-5a-nitro-2,3,5a,6,7',8,8a,8',9,9a-decahydro-1*H*,2'*H*,5*H*-spiro[cyclopenta[*f*]indolizine-7,3'-pyrano[2,3-*g*]indole]-2',5,9'(1'*H*)-trione (**24**) (6.4 mg, 0.0132 mmol, 1.0 equiv) and a stir bar was added Me_3OBF_4 (23.5 mg, 0.159 mmol, 12.0 equiv) and activated 4 Å MS (64 mg) in a nitrogen atmosphere glove box. The reaction vessel was then removed from the glove box and CH_2Cl_2 (0.90 mL, 0.015M) was added by syringe under a nitrogen atmosphere and the mixture was stirred at 45 ºC for 16 h. After cooling to 0 ºC, anhydrous MeOH (0.90 mL) was added drop-wise followed by $NaCNBH_3$ (16.7 mg, 0.264 mmol, 20.0 equiv) in one portion. After 5 min, more $NaCNBH_3$ (16.7 mg, 0.264 mmol, 20.0 equiv) was added in one portion and the reaction mixture stirred at 0 ºC for 30 min. The resulting reaction mixture was subsequently quenched by the addition of saturated aqueous $NaHCO_3$ (3.0 mL) and extracted with EtOAc (4 x 3.0 mL). The combined organic extracts were dried over Na_2SO_4, filtered, and concentrated *in vacuo*. The resulting yellow oil was purified by silica gel chromatography (5 mL SiO_2 with 1% to 2% to 3% to 5% methanol:toluene) to yield (–)-17-hydroxy-citrinalin B (**7**) (3.0 mg, 0.0064 mmol, 48%) as a yellow oil, methyl ether (**7b**) (1.2 mg, 0.0025 mmol, 19%) as a yellow oil, and recovered **24** (0.7 mg, 0.0014 mmol, 11% recovery).

(–)-17-hydroxy-citrinalin B (**7**): TLC (methanol:toluene, 1:9 v/v): R_f= 0.31; ^{1}**H NMR** (600 MHz, CD_3OD) δ = 7.47 (d, *J* = 8.4 Hz, 1H), 6.60 (d, *J* = 8.4 Hz, 1H), 4.18 (ddd, *J* = 7.3, 4.6, 2.3 Hz, 1H), 3.95 (d, *J* = 9.1 Hz, 1H), 3.85 (d, *J* = 13.1 Hz, 1H), 3.07 – 3.03 (m, 1H), 2.86 – 2.76 (m, 3H), 2.67 – 2.60 (m, 2H), 2.39 (td, *J* = 14.5, 14.1, 9.5 Hz, 1H), 2.28 (dt, *J* = 14.3, 7.3 Hz, 1H), 2.10 – 1.92 (m, 3H), 1.74 – 1.66 (m, 2H), 1.50 (s, 3H), 1.49 (s, 3H), 1.12 (s, 3H), 0.88 (s, 3H); ^{13}**C NMR** (150 MHz, CD_3OD) δ = 195.19, 185.00, 161.25, 144.54, 134.17, 121.35, 111.10, 106.84, 96.06, 80.83, 72.98, 67.67, 66.54, 60.75, 53.64, 50.74, 49.3, 45.32, 44.00, 34.16, 27.05, 27.03, 24.43, 23.80, 21.46; **IR** (neat) ν_{max}: 3282, 2965, 2887, 1720, 1624, 1550, 1460, 1367 cm^{-1}; **HRMS** (ESI) calcd for $C_{25}H_{32}O_6N_3$ ($[M+H]^+$): 470.2286, found 470.2281.

methyl ether (**7b**): $[\alpha]^{22}_{D}$= -95.2 degrees (*c* = 1.46 , MeOH); TLC (methanol:toluene, 1:9 v/v): R_f= 0.41; **^{1}H NMR** (600 MHz, CD_3OD) δ = 7.46 (d, *J* = 8.4 Hz, 1H), 6.60 (d, *J* = 8.4 Hz, 1H), 3.92 (d, *J* = 9.2 Hz, 1H), 3.87 – 3.81 (m, 2H), 3.33 (s, 3H), 3.02 (td, *J* = 8.9, 1.8 Hz, 1H), 2.86 – 2.78 (m, 3H), 2.66 – 2.62 (m, 2H), 2.43 (ddd, *J* = 14.5, 12.7, 9.3 Hz, 1H), 2.19 – 2.13 (m, 1H), 2.10 – 2.01 (m, 2H), 1.80 (dtd, *J* = 13.7, 9.0, 2.5 Hz, 1H), 1.73 (dd, *J* = 14.5, 3.7 Hz, 1H), 1.50 (s, 3H), 1.48 (s, 3H), 1.12 (s, 3H), 0.87 (s, 3H); **^{13}C NMR** (150 MHz, CD_3OD) δ = 195.23, 184.96, 161.21, 144.50, 134.17, 121.29, 111.10, 106.80, 95.96, 82.41, 80.82, 67.34, 66.55, 60.71, 57.46, 53.91, 50.74, 49.3, 45.27, 44.02, 30.99, 27.05, 27.00, 24.36, 23.76, 21.32; **IR** (neat) ν_{max}: 3237, 2976, 2934, 1726, 1672, 1610, 1544, 1465, 1370 cm^{-1}; **HRMS** (ESI) calcd for $C_{26}H_{34}O_6N_3$ ($[M+H]^+$): 484.2442, found 484.2437.

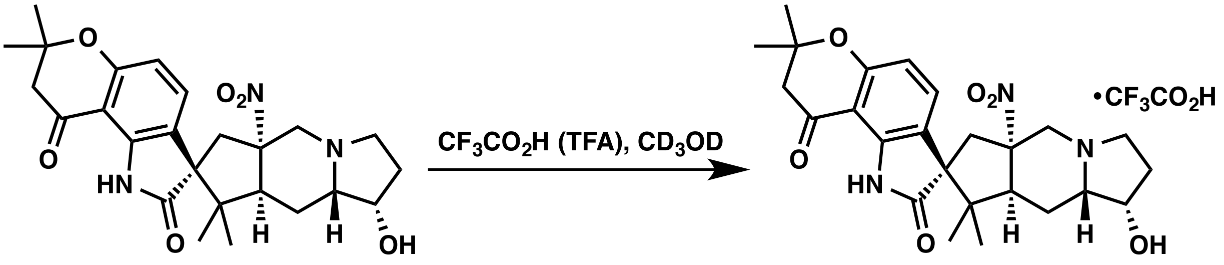

ent-17-hydroxycitrinalin B (7) *ent*-17-hydroxycitrinalin B•TFA (7a)

To a solution of (–)-17-hydroxycitrinalin B (**7**) (1.7 mg, 0.0036 mmol, 1.0 equiv) in methanol-d_4 (1.0 mL) was added trifluoroacetic acid (TFA) (0.033 mL, 0.432 mmol, 120.0 equiv) at room temperature. The solvents were then removed *in vacuo*.

(–)-17-hydroxycitrinalin B•TFA (**7a**): $[\alpha]^{22}_{D}$= -70.5 degrees (*c* = 1.8, MeOH) **^{1}H NMR** (600 MHz, CD_3OD) δ = 7.42 (d, *J* = 8.4 Hz, 1H), 6.60 (d, *J* = 8.4 Hz, 1H), 4.49 (dd, *J* = 5.8, 3.2 Hz, 1H), 4.41 (d, *J* = 14.5 Hz, 1H), 4.00 (d, *J* = 8.7 Hz, 1H), 3.80 – 3.71 (m, 2H), 3.25 (d, *J* = 13.3 Hz, 1H), 3.20 – 3.12 (m, 1H), 3.09 (d, *J* = 16.1 Hz, 1H), 2.77 (s, 2H), 2.75 – 2.67 (m, 1H), 2.66 – 2.58 (m, 1H), 2.55 – 2.45 (m, 1H), 2.12 (dd, *J* = 15.7, 3.6 Hz, 1H), 2.02 (dt, *J* = 14.0, 8.5 Hz, 1H), 1.46 (s, 3H), 1.45 (s, 3H), 1.17 (s, 3H), 0.88 (s, 3H); **^{13}C NMR** (150 MHz, CD_3OD) δ = 195.09, 184.21, 161.56, 144.58, 134.09, 120.23, 111.44, 107.03, 93.76, 80.97, 70.19, 69.46, 61.00, 60.69, 54.28, 51.11, 49.3, 46.09, 43.19, 32.52, 27.05, 26.99, 23.02 (2C), 20.02.

Reported Data for (+)-17-hydroxy-citrinalin B:[6] $[\alpha]_D$ +76.9 degrees (*c* 1.6, MeOH)); **^{1}H NMR** (600 MHz, CD_3OD) δ = 7.38 (d, *J* = 8.4 Hz, 1H), 6.60 (d, *J* = 8.4 Hz, 1H), 4.47 (dd, *J* = 5.4, 3.2 Hz, 1H), 4.40 (d, *J* = 14.5 Hz, 1H), 3.99 (d, *J* = 8.7 Hz, 1H), 3.76 (m, 1H), 3.70 (d, *J* = 14.5 Hz, 1H), 3.20 (bd, *J* = 13.5 Hz, 1H), 3.12 (m, 1H), 3.08 (d, *J* = 16.1 Hz, 1H), 2.77 (d, *J* = 16.8 Hz, 1H), 2.75 (d, *J* = 16.8 Hz, 1H), 2.67 (d, *J* = 16.1 Hz, 1H), 2.60 (ddd, *J* = 15.6, 13.5, 8.7 Hz, 1H), 2.48 (m, 1H), 2.10 (dd, *J* = 15.6, 3.9 Hz, 1H), 2.00 (dt, *J* = 13.0, 8.7 Hz, 1H), 1.45 (s, 3H), 1.44 (s, 3H), 1.15 (s, 3H), 0.88 (s, 3H); **^{13}C NMR** (150 MHz, CD_3OD) δ = 195.02, 184.21, 161.58, 144.67, 133.98, 120.25, 111.38, 107.06, 93.74, 80.92, 70.19, 69.48, 61.00, 60.3 54.26, 51.09, 49.0, 45.99, 43.28, 32.55, 27.03, 26.99, 23.03 (2C), 20.05.[11]

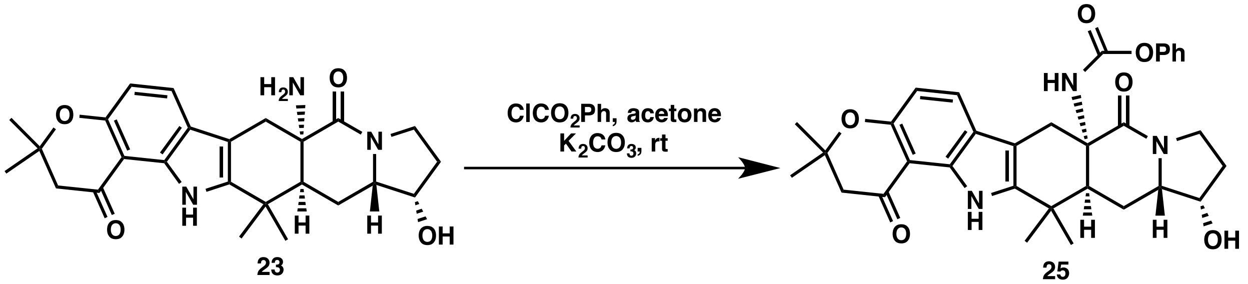

To a solution of (7a*S*,12*S*,12a*S*,13a*S*)-7a-amino-12-hydroxy-3,3,14,14-tetramethyl-2,3,7,7a,10,11,12,12a,13,13a,14,15-dodecahydroindolizino[6,7-*h*]pyrano[3,2-*a*]carbazole-1,8-dione (**23**) (20 mg, 0.0457 mmol, 1.0 equiv) and K_2CO_3 (63 mg, 0.457 mmol, 10.0 equiv) in anhydrous acetone (1.0 mL, 0.05M) was added phenyl chloroformate (0.060 mL, 0.457 mmol, 10.0 equiv) dropwise at room temperature. The resulting solution was stirred for 5 h then additional phenyl chloroformate (0.060 mL, 0.457 mmol, 10.0 equiv) was added dropwise, followed by more K_2CO_3 (63 mg, 0.457 mmol, 10.0 equiv). The resulting solution was stirred at room temperature for 16 h, at which time H_2O (2 mL) was added and the aqueous layer was extracted with ethyl acetate (4 x 2 mL). The combined organic extracts were dried over Na_2SO_4, filtered, and concentrated *in vacuo*. The resulting oil residue was purified by silica gel chromatography (5 mL SiO_2 with 1% to 2% to 5% methanol:dichloromethane) to yield **25** (23 mg, 0.0412 mmol, 90%) as a yellow oil. TLC (methanol:dichloromethane, 1:19 v/v): R_f= 0.23; **^{1}H NMR** (600 MHz, $CDCl_3$) δ = 9.79 (s, 1H), 7.53 (d, *J* = 8.5 Hz, 1H), 7.28 – 7.26 (m, 1H), 7.26 – 7.24 (m, 1H), 7.12 (t, *J* = 7.4 Hz, 1H), 7.04 (d, *J* = 8.0 Hz, 2H), 6.68 (d, *J* = 8.5 Hz, 1H), 5.38 (s, 1H), 4.06 (dt, *J* = 12.2, 8.3 Hz, 2H), 3.42 – 3.36 (m, 1H), 3.26 (td, *J* = 11.5, 3.6 Hz, 1H), 3.20

---

[11] The spectroscopic data for the TFA salt were consistent with those previously reported for the neutral form of the natural product 17-hydroxy-citrinalin B (**7**); see spectra below, pages 63-64.

(dd, J = 13.7, 5.7 Hz, 1H), 3.12 (d, J = 16.7 Hz, 1H), 2.98 (d, J = 16.7 Hz, 1H), 2.78 (s, 2H), 2.76 – 2.69 (m, 1H), 2.43 (dd, J = 14.2, 5.7 Hz, 1H), 2.05 – 2.01 (m, 1H), 1.91 (ddd, J = 13.8, 9.3, 3.6 Hz, 1H), 1.78 (td, J = 14.0, 9.4 Hz, 1H), 1.60 (s, 3H), 1.51 (s, 6H), 1.44 (s, 3H); **^{13}C NMR** (150 MHz, $CDCl_3$) δ = 194.3, 171.1, 157.7, 154.4, 150.9, 139.8, 134.3, 129.3, 127.0, 125.4, 121.9, 121.5, 110.2, 105.5, 102.3, 79.7, 74.4, 59.5, 58.7, 48.9, 42.4, 40.8, 34.8, 32.0, 30.8, 28.5, 27.9, 26.8, 26.7, 22.3; **IR** (neat) ν_{max}: 3442, 3348, 2974, 1733, 1647, 1619, 1580, 1461, 1370, 1203 cm^{-1}; **HRMS** (ESI) calcd for $C_{32}H_{36}O_6N_3$ ($[M+H]^+$): 558.2599, found 558.2617.

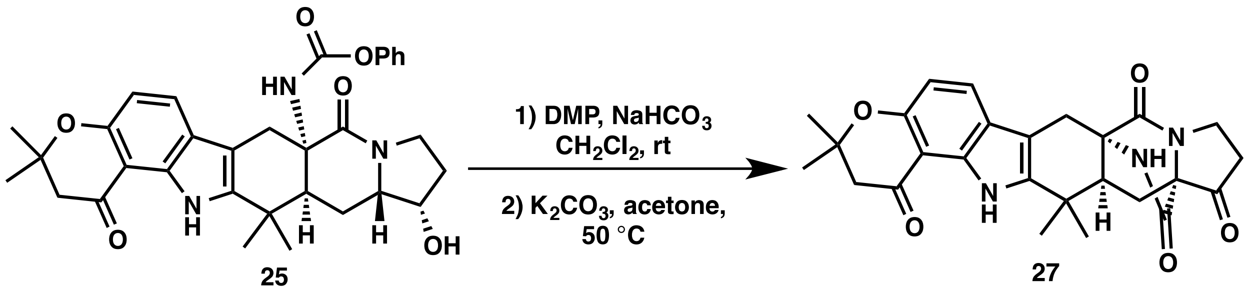

To a solution of phenyl ((7a*S*,12*S*,12a*S*,13a*S*)-12-hydroxy-3,3,14,14-tetramethyl-1,8-dioxo-1,2,3,7,10,11,12,12a,13,13a,14,15-dodecahydroindolizino[6,7-*h*]pyrano[3,2-*a*]carbazol-7a(8*H*)-yl)carbamate (**25**) (20.0 mg, 0.0359 mmol, 1.0 equiv) and $NaHCO_3$ (15.0 mg, 0.180 mmol, 5.0 equiv) in CH_2Cl_2 (0.720 mL, 0.05M) was added Dess-Martin periodinane (DMP) (23.0 mg, 0.0538 mmol, 1.5 equiv) in three portions (3 x 7.6 mg) at 5 minute intervals. The resulting solution was stirred at room temperature for 30 min then additional DMP (15.2 mg, 0.0359 mmol, 1.0 equiv) was added in two portions (2 x 7.6 mg) at 5 minute intervals. After 20 minutes at room temperature saturated aqueous $NaHCO_3$ (2.0 mL) was added and the mixture was stirred until the organic layer was no longer cloudy. The layers were separated and the aqueous layer was extracted with CH_2Cl_2 (4 x 2 mL) and the combined organic extracts were dried over Na_2SO_4, filtered, and concentrated *in vacuo*. The reaction mixture was subsequently diluted with CH_2Cl_2 and passed through a short column containing silica gel (2 mL) with 2:3 to 4:1 ethyl acetate:hexanes. The fractions containing the product were collected and concentrated *in vacuo*. [TLC (ethyl acetate:hexanes, 4:1 v/v): R_f=0.26]. The residue was dissolved in anhydrous acetone (1.4 mL, 0.025M) and K_2CO_3 (9.9 mg, 0.0718 mmol, 2.0 equiv) was added at room temperature and then the reaction was heated to 50 °C. After 2 h, the solution was cooled to 0 °C and saturated aqueous NH_4Cl (1 mL) was added and the aqueous layer was extracted with ethyl acetate (4 x 2 mL). The combined organic extracts were dried over Na_2SO_4, filtered and

concentrated *in vacuo*. The resulting residue was purified by silica gel chromatography (4 mL SiO_2 with 1% to 3% to 5% methanol:dichloromethane) to yield **27** (6.9 mg, 0.0150 mmol, 42% over 2-steps) as a yellow oil. TLC (methanol:dichloromethane, 1:19 v/v): R_f= 0.31; **^{1}H NMR** (600 MHz, $CDCl_3$) δ = 9.67 (s, 1H), 7.61 (d, *J* = 8.5 Hz, 1H), 6.91 (s, 1H), 6.65 (d, *J* = 8.5 Hz, 1H), 3.95 (td, *J* = 11.1, 4.5 Hz, 1H), 3.81 (d, *J* = 15.4 Hz, 1H), 3.69 (dt, *J* = 11.9, 8.2 Hz, 1H), 2.93 (ddd, *J* = 18.2, 10.4, 7.4 Hz, 1H), 2.82 – 2.77 (m, 1H), 2.75 (s, 2H), 2.71 (d, *J* = 15.4 Hz, 1H), 2.66 (dd, *J* = 10.3, 4.7 Hz, 1H), 2.51 (dd, *J* = 13.6, 10.4 Hz, 1H), 2.01 (dd, *J* = 13.6, 4.7 Hz, 1H), 1.50 (s, 6H), 1.33 (s, 3H), 1.11 (s, 3H); **^{13}C NMR** (150 MHz, $CDCl_3$) δ = 204.3, 194.3, 169.6, 169.5, 157.9, 139.0, 134.5, 127.8, 121.2, 110.0, 105.3, 104.5, 79.7, 67.0, 61.5, 48.9, 48.8, 38.6, 36.5, 34.9, 28.5, 27.8, 26.8, 26.7, 24.6, 22.6; **IR** (neat) ν_{max}: 3441, 3234, 2972, 2929, 1768, 1695, 1583, 1461, 1373 cm^{-1}; **HRMS** (ESI) calcd for $C_{26}H_{28}O_5N_3$ ($[M+H]^+$): 462.2023, found 462.2022.

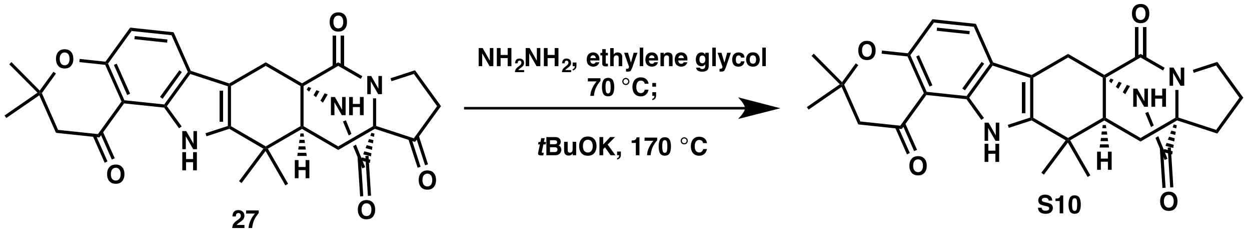

27 S10

Stock solution A: Hydrazine (5.0 μL, 0.15 mmol) in deoxygenated (via three cycles of freeze/pump/thaw) ethylene glycol (5.0 mL). To a 4 mL vial equipped with a stir bar and (7a*S*,12a*R*,13a*S*)-3,3,14,14-tetramethyl-2,3,10,11,13,13a,14,15-octahydro-8*H*,12*H*-7a,12a-(epiminomethano)indolizino[6,7-*h*]pyrano[3,2-*a*]carbazole-1,8,12,16(7*H*)-tetraone (**27**) (6.2 mg, 0.013 mol, 1.0 equiv) was added 0.50 mL of stock solution A (0.015 mmol, 1.1 equiv) under a N_2 atmosphere. The solution was then heated at 70 °C for 17 h, at which time the solution was cooled to room temperature and *t*BuOK (7.5 mg, 0.067 mmol, 5.0 equiv) was added in one portion at room temperature and the solution was then placed in a preheated heating block at 170 °C. After 2 h, the reaction was cooled to room temperature and saturated aqueous NH_4Cl (2.0 mL) was added and the aqueous layer was extracted with ethyl acetate (4 x 2 mL). The combined organic layers were dried over Na_2SO_4, filtered, and concentrated *in vacuo*. The resulting residue was purified by silica gel chromatography (2 mL SiO_2 with 1% to 3% to 5% methanol:dichloromethane) to yield **S10** (3.3 mg, 0.0074 mmol, 57%) as a beige powder. TLC (methanol:dichloromethane, 1:19 v/v): R_f= 0.33; **^{1}H NMR** (600 MHz, $CDCl_3$) δ = 9.66 (s, 1H),

7.61 (d, J = 8.6 Hz, 1H), 6.66 (d, J = 8.6 Hz, 1H), 5.95 (s, 1H), 3.82 (d, J = 15.0 Hz, 1H), 3.55 (dt, J = 12.5, 6.5 Hz, 1H), 3.41 (dt, J = 11.4, 7.1 Hz, 1H), 2.81 (dt, J = 13.3, 6.7 Hz, 1H), 2.75 (s, 2H), 2.61 (d, J = 15.0 Hz, 1H), 2.60 – 2.56 (m, 1H), 2.25 (dd, J = 13.5, 10.3 Hz, 1H), 2.06 – 1.97 (m, 3H), 1.89 (dt, J = 14.5, 7.5 Hz, 1H), 1.50 (s, 6H), 1.34 (s, 3H), 1.13 (s, 3H); **^{13}C NMR** (150 MHz, $CDCl_3$) δ = 194.3, 173.7, 168.6, 157.8, 139.5, 134.5, 127.7, 121.4, 109.9, 105.3, 104.8, 79.6, 66.7, 60.6, 49.6, 48.9, 44.3, 34.9, 31.1, 29.5, 28.6, 26.9, 26.6, 25.1, 24.7, 22.3; **IR** (neat) ν_{max}: 3441, 2964, 2930, 1686, 1656, 1618, 1582, 1457, 1370 cm^{-1}; **HRMS** (ESI) calcd for $C_{26}H_{29}O_4N_3Na$ ([M+Na]$^+$): 470.2050, found 470.2045.

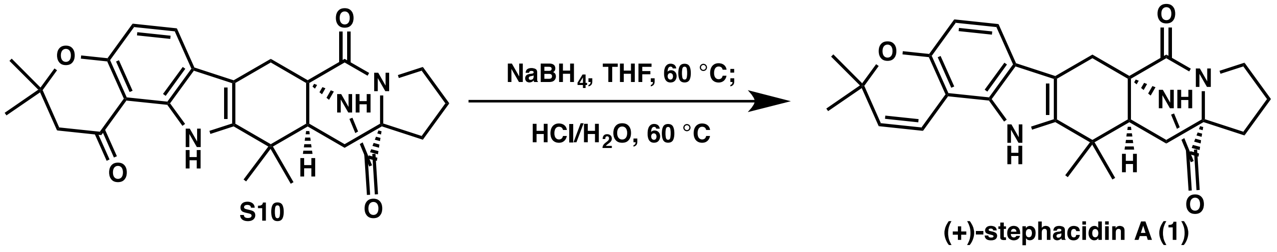

To a solution of (7a*S*,12a*S*,13a*S*)-3,3,14,14-tetramethyl-2,3,11,12,13,13a,14,15-octahydro-8*H*,10*H*-7a,12a-(epiminomethano)indolizino[6,7-*h*]pyrano[3,2-*a*]carbazole-1,8,16(7*H*)-trione (**S10**) (3.2 mg, 0.0072 mmol, 1.0 equiv) in THF (0.20 mL, 0.035M) was added $NaBH_4$ (2.7 mg, 0.072 mmol, 10.0 equiv) in one portion at room temperature. The resulting solution was heated at 60 °C for 16 h, at which time the solution was cooled to room temperature and aqueous HCl (0.25 mL, 0.6M) was added dropwise. After bubbling had ceased, the solution was heated to 60 °C for 30 min and then cooled to room temperature. Saturated aqueous $NaHCO_3$ (1.5 mL) was added slowly and the aqueous layer was extracted with ethyl acetate (4 x 1.5 mL). The combined organic extracts were dried over Na_2SO_4, filtered, and concentrated *in vacuo*. The resulting residue was purified by silica gel chromatography (2 mL SiO_2 with 1% to 3% to 5% methanol:dichloromethane) to yield **(+)-stephacidin A** (**1**) (2.2 mg, 0.0051 mmol, 71%) as a white powder. **M.P.** >340 °C (decomp); $[\alpha]^{22}_D$ = + 79.1 degrees (c = 0.63, 1:1 CH_2Cl_2/MeOH)[12]; TLC (methanol:dichloromethane, 1:19 v/v): R_f = 0.35; **^{1}H NMR** (600 MHz, $(CD_3)_2SO$) δ = 10.45 (s, 1H), 8.68 (s, 1H), 7.09 (d, J = 8.3 Hz, 1H), 6.93 (d, J = 9.8 Hz, 1H), 6.47 (d, J = 8.3 Hz, 1H),

[12] P. S. Baran, C. A. Guerrero, B. D. Hafensteiner, N. B. Ambhaikar, *Angew. Chem. Int. Ed.,* 2005, **44**, 3892. Synthetic (+)-stephacidin A $[\alpha]_D$ = + 68.5 degrees (c = 0.35, 1:1 CH_2Cl_2/MeOH): natural (+)-stephacidin A $[\alpha]_D$ = + 61.5 degrees (c = 0.26, 1:1 CH_2Cl_2/MeOH)

5.72 (d, J = 9.8 Hz, 1H), 3.38 – 3.35 (m, 1H), 3.32 – 3.29 (m, 1H), 3.27 – 3.21 (m, 1H), 2.63 (d, J = 15.5 Hz, 1H), 2.55 – 2.52 (m, 1H), 2.42 (dd, J = 10.1, 4.7 Hz, 1H), 2.10 – 2.02 (m, 1H), 2.02 – 1.94 (m, 2H), 1.88 – 1.79 (m, 2H), 1.37 (s, 3H), 1.36 (s, 3H), 1.28 (s, 3H), 1.00 (s, 3H); **^{13}C NMR** (150 MHz, $(CD_3)_2SO$) δ = 173.0, 168.4, 147.5, 139.6, 132.8, 128.9, 121.5, 118.2, 117.5, 108.6, 104.8, 103.8, 75.0, 66.0, 59.6, 49.2, 43.5, 34.6, 30.1, 28.7, 28.0, 27.1, 27.0, 24.0, 23.8, 21.5; **IR** (neat) ν_{max}: 3325, 2924, 1675, 1638, 1459 cm^{-1}; **HRMS** (ESI) calcd for $C_{26}H_{29}O_3N_3Na$ ([M+Na]$^+$): 454.2101, found 454.2097. The spectroscopic data were consistent with those previously reported.[13]

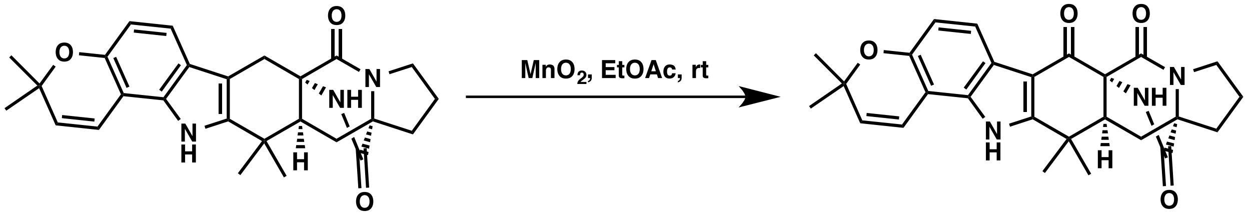

To a solution of (+)-stephacidin A (**1**) (3.6 mg, 0.0083 mmol, 1.00 equiv) in reagent grade ethyl acetate (3.6 mL, 0.0023M) was added MnO_2 (84 mg, 1.00 mmol, 120.0 equiv) in four portions (4 x 21 mg) at 2 h intervals. 30 minutes after the last addition, the reaction was filtered through Celite®, washed with ethyl acetate and the solvent removed *in vacuo*. The resulting residue was purified by silica gel chromatography (2 mL SiO_2 with 1% to 2% to 3% methanol:dichloromethane) to yield **(+)-notoamide I** (**2**) (1.2 mg, 0.0027 mmol, 32%) as a white powder. $[\alpha]^{22}_D$ = + 74.2 degrees (c = 0.22, 1:1 $CHCl_3$/MeOH); TLC (methanol:dichloromethane, 1:19 v/v): R_f = 0.30; **^{1}H NMR** (600 MHz, $(CD_3)_2SO$) δ = 11.65 (s, 1H), 8.73 (s, 1H), 7.76 (d, J = 8.4 Hz, 1H), 7.00 (d, J = 9.9 Hz, 1H), 6.70 (d, J = 8.4 Hz, 1H), 5.85 (d, J = 9.9 Hz, 1H), 3.38 – 3.34 (m, 1H), 3.32 – 3.29 (m, 1H), 2.82 (dd, J = 9.9, 5.6 Hz, 1H), 2.55 – 2.52 (m, 1H), 2.14 – 2.00 (m, 3H), 1.89 – 1.82 (m, 2H), 1.42 (s, 3H), 1.40 (s, 3H), 1.39 (s, 3H), 1.23 (s, 3H); **IR** (neat) ν_{max}: 3268, 2972, 2930, 1717, 1662, 1590, 1456, 1378 cm^{-1}; **HRMS**

[13] (a) P. S. Baran, C. A. Guerrero, N. B. Ambhaikar, B. D. Hafensteiner, *Angew. Chem. Int. Ed.,* 2005, **44**, 606.; (b) T. J. Greshock, A. W. Grubbs, S. Tsukamoto, R. M. Williams, *Angew. Chem. Int. Ed.,* 2007, **46**, 2262. See spectra below: pages 69-70.

(ESI) calcd for $C_{26}H_{28}O_4N_3$ ($[M+H]^+$): 446.2074, found 446.2078. The spectroscopic data were consistent with those previously reported.[14]

[14] S. Tsukamoto, H. Kato, M. Samizo, Y. Nojiri, H. Onuki, H Hirota, T. Ohta, *J. Nat. Prod.,* 2008, **71**, 2064. Natural (+)-notoamide I $[\alpha]_D^{29}$ = + 31.0 degrees (*c* = 0.1, 1:1 $CHCl_3$/MeOH). See spectra below: page 71.

III. Crystallographic Data for Compounds 18 and (+)-stephacidin A (1)

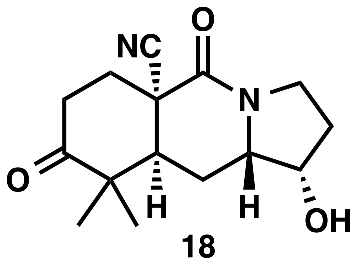

18

A colorless blade 0.100 x 0.040 x 0.020 mm in size was mounted on a Cryoloop with Paratone oil. Data were collected in a nitrogen gas stream at 100(2) K using phi and omega scans. Crystal-to-detector distance was 60 mm and exposure time was 10 seconds per frame using a scan width of 1.0°. Data collection was 99.9% complete to 67.000° in θ. A total of 10468 reflections were collected covering the indices, -9<=*h*<=9, -8<=*k*<=7, -15<=*l*<=15. 2538 reflections were found to be symmetry independent, with an R_{int} of 0.0262. Indexing and unit cell refinement indicated a primitive, monoclinic lattice. The space group was found to be P 21 (No. 4). The data were integrated using the Bruker SAINT software program and scaled using the SADABS software program. Solution by direct methods (SIR-2011) produced a complete heavy-atom phasing model consistent with the proposed structure. All non-hydrogen atoms were refined anisotropically by full-matrix least-squares (SHELXL-2012). All hydrogen atoms were placed using a riding model. Their positions were constrained relative to their parent atom using the appropriate HFIX command in SHELXL-2012. Absolute stereochemistry was unambiguously determined to be *R* at C1 and *S* at C6, C8, and C9, respectively. CCDC # 1400755 (**18**) contains the supplementary crystallographic data for this paper. These data can be obtained free of charge from The Cambridge Crystallographic Data Centre via www.ccdc.cam.ac.uk/data_request/cif.

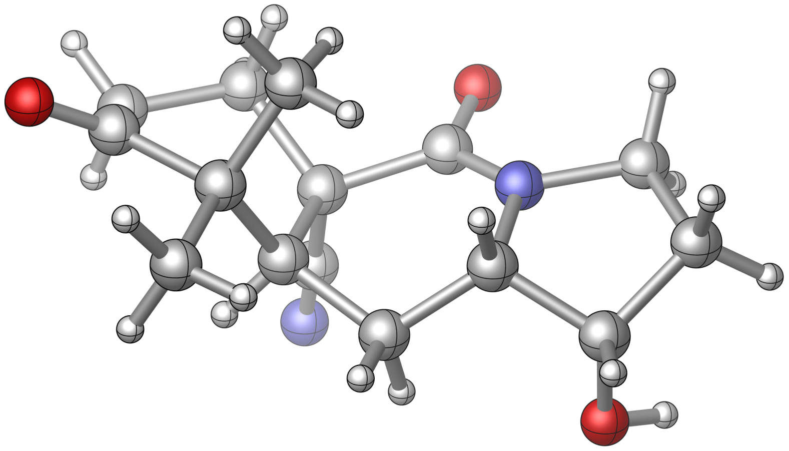

CYLview of **18**

Table 1. Crystal data and structure refinement for sarpong38.

X-ray ID	sarpong38	
Sample/notebook ID	EM03-093B	
Empirical formula	C15 H20 N2 O3	
Formula weight	276.33	
Temperature	100(2) K	
Wavelength	1.54178 Å	
Crystal system	Monoclinic	
Space group	P 21	
Unit cell dimensions	a = 7.9384(5) Å	α= 90°.
	b = 7.0246(5) Å	β= 98.464(4)°.
	c = 12.9394(8) Å	γ = 90°.
Volume	713.69(8) Å^3	
Z	2	
Density (calculated)	1.286 Mg/m^3	
Absorption coefficient	0.734 mm^{-1}	
F(000)	296	
Crystal size	0.100 x 0.040 x 0.020 mm^3	
Crystal color/habit	colorless blade	
Theta range for data collection	3.453 to 68.368°.	
Index ranges	-9<=h<=9, -8<=k<=7, -15<=l<=15	
Reflections collected	10468	
Independent reflections	2538 [R(int) = 0.0262]	
Completeness to theta = 67.000°	99.9 %	
Absorption correction	Semi-empirical from equivalents	
Max. and min. transmission	0.929 and 0.864	
Refinement method	Full-matrix least-squares on F^2	
Data / restraints / parameters	2538 / 1 / 184	
Goodness-of-fit on F^2	1.067	
Final R indices [I>2sigma(I)]	R1 = 0.0390, wR2 = 0.1064	
R indices (all data)	R1 = 0.0403, wR2 = 0.1078	
Absolute structure parameter	0.09(8)	
Extinction coefficient	n/a	
Largest diff. peak and hole	0.648 and -0.174 e.Å^{-3}	

Table 2. Atomic coordinates (x 10^4) and equivalent isotropic displacement parameters (Å^{2}x 10^3) for sarpong38. U(eq) is defined as one third of the trace of the orthogonalized U^{ij} tensor.

	x	y	z	U(eq)
C(1)	3422(3)	9265(4)	7290(2)	21(1)
C(2)	4152(3)	10654(4)	6533(2)	25(1)
C(3)	2904(3)	10899(5)	5523(2)	30(1)
C(4)	1232(3)	11738(4)	5726(2)	27(1)
C(5)	650(3)	11278(4)	6786(2)	24(1)
C(6)	1430(3)	9323(4)	7160(2)	21(1)
C(7)	845(3)	8469(4)	8138(2)	24(1)
C(8)	1608(3)	9456(4)	9136(2)	25(1)
C(9)	1460(3)	8392(5)	10146(2)	29(1)
C(10)	2824(4)	9376(5)	10920(2)	32(1)
C(11)	4310(4)	9715(4)	10300(2)	28(1)
C(12)	4368(3)	9595(4)	8408(2)	22(1)
C(13)	3927(3)	7318(4)	7019(2)	26(1)
C(14)	1176(4)	13004(4)	7498(2)	30(1)
C(15)	-1302(3)	11140(5)	6608(2)	31(1)
N(1)	3476(3)	9679(3)	9202(2)	23(1)
N(2)	4323(3)	5815(4)	6786(2)	40(1)
O(1)	400(3)	12762(4)	5101(2)	42(1)
O(2)	1768(3)	6432(3)	10013(2)	33(1)
O(3)	5946(2)	9707(3)	8531(1)	30(1)

Table 3. Bond lengths [Å] and angles [°] for sarpong38.

C(1)-C(13)	1.481(4)	C(8)-C(9)	1.525(4)
C(1)-C(12)	1.546(3)	C(8)-H(8)	1.0000
C(1)-C(2)	1.554(4)	C(9)-O(2)	1.413(4)
C(1)-C(6)	1.566(3)	C(9)-C(10)	1.528(4)
C(2)-C(3)	1.529(4)	C(9)-H(9)	1.0000
C(2)-H(2A)	0.9900	C(10)-C(11)	1.539(4)
C(2)-H(2B)	0.9900	C(10)-H(10A)	0.9900
C(3)-C(4)	1.510(4)	C(10)-H(10B)	0.9900
C(3)-H(3A)	0.9900	C(11)-N(1)	1.476(3)
C(3)-H(3B)	0.9900	C(11)-H(11A)	0.9900
C(4)-O(1)	1.204(4)	C(11)-H(11B)	0.9900
C(4)-C(5)	1.544(4)	C(12)-O(3)	1.242(3)
C(5)-C(15)	1.536(3)	C(12)-N(1)	1.333(3)
C(5)-C(14)	1.542(4)	C(13)-N(2)	1.155(4)
C(5)-C(6)	1.554(4)	C(14)-H(14A)	0.9800
C(6)-C(7)	1.532(3)	C(14)-H(14B)	0.9800
C(6)-H(6)	1.0000	C(14)-H(14C)	0.9800
C(7)-C(8)	1.512(4)	C(15)-H(15A)	0.9800
C(7)-H(7A)	0.9900	C(15)-H(15B)	0.9800
C(7)-H(7B)	0.9900	C(15)-H(15C)	0.9800
C(8)-N(1)	1.480(3)	O(2)-H(2)	0.8400

C(13)-C(1)-C(12)	104.4(2)	C(4)-C(3)-C(2)	111.7(2)
C(13)-C(1)-C(2)	106.9(2)	C(4)-C(3)-H(3A)	109.3
C(12)-C(1)-C(2)	108.7(2)	C(2)-C(3)-H(3A)	109.3
C(13)-C(1)-C(6)	107.7(2)	C(4)-C(3)-H(3B)	109.3
C(12)-C(1)-C(6)	116.1(2)	C(2)-C(3)-H(3B)	109.3
C(2)-C(1)-C(6)	112.4(2)	H(3A)-C(3)-H(3B)	107.9
C(3)-C(2)-C(1)	110.8(2)	O(1)-C(4)-C(3)	121.7(2)
C(3)-C(2)-H(2A)	109.5	O(1)-C(4)-C(5)	121.0(3)
C(1)-C(2)-H(2A)	109.5	C(3)-C(4)-C(5)	117.3(2)
C(3)-C(2)-H(2B)	109.5	C(15)-C(5)-C(14)	108.5(2)
C(1)-C(2)-H(2B)	109.5	C(15)-C(5)-C(4)	107.9(2)
H(2A)-C(2)-H(2B)	108.1	C(14)-C(5)-C(4)	106.0(2)

C(15)-C(5)-C(6)	109.7(2)
C(14)-C(5)-C(6)	116.6(2)
C(4)-C(5)-C(6)	107.7(2)
C(7)-C(6)-C(5)	116.6(2)
C(7)-C(6)-C(1)	109.0(2)
C(5)-C(6)-C(1)	113.9(2)
C(7)-C(6)-H(6)	105.4
C(5)-C(6)-H(6)	105.4
C(1)-C(6)-H(6)	105.4
C(8)-C(7)-C(6)	113.2(2)
C(8)-C(7)-H(7A)	108.9
C(6)-C(7)-H(7A)	108.9
C(8)-C(7)-H(7B)	108.9
C(6)-C(7)-H(7B)	108.9
H(7A)-C(7)-H(7B)	107.8
N(1)-C(8)-C(7)	111.7(2)
N(1)-C(8)-C(9)	101.9(2)
C(7)-C(8)-C(9)	115.8(2)
N(1)-C(8)-H(8)	109.0
C(7)-C(8)-H(8)	109.0
C(9)-C(8)-H(8)	109.0
O(2)-C(9)-C(8)	109.7(2)
O(2)-C(9)-C(10)	113.7(2)
C(8)-C(9)-C(10)	101.7(2)
O(2)-C(9)-H(9)	110.5
C(8)-C(9)-H(9)	110.5
C(10)-C(9)-H(9)	110.5
C(9)-C(10)-C(11)	104.6(2)
C(9)-C(10)-H(10A)	110.8
C(11)-C(10)-H(10A)	110.8
C(9)-C(10)-H(10B)	110.8
C(11)-C(10)-H(10B)	110.8
H(10A)-C(10)-H(10B)	108.9
N(1)-C(11)-C(10)	103.3(2)
N(1)-C(11)-H(11A)	111.1
C(10)-C(11)-H(11A)	111.1
N(1)-C(11)-H(11B)	111.1
C(10)-C(11)-H(11B)	111.1
H(11A)-C(11)-H(11B)	109.1
O(3)-C(12)-N(1)	122.6(2)
O(3)-C(12)-C(1)	118.1(2)
N(1)-C(12)-C(1)	119.2(2)
N(2)-C(13)-C(1)	178.5(3)
C(5)-C(14)-H(14A)	109.5
C(5)-C(14)-H(14B)	109.5
H(14A)-C(14)-H(14B)	109.5
C(5)-C(14)-H(14C)	109.5
H(14A)-C(14)-H(14C)	109.5
H(14B)-C(14)-H(14C)	109.5
C(5)-C(15)-H(15A)	109.5
C(5)-C(15)-H(15B)	109.5
H(15A)-C(15)-H(15B)	109.5
C(5)-C(15)-H(15C)	109.5
H(15A)-C(15)-H(15C)	109.5
H(15B)-C(15)-H(15C)	109.5
C(12)-N(1)-C(11)	121.9(2)
C(12)-N(1)-C(8)	126.3(2)
C(11)-N(1)-C(8)	111.15(19)
C(9)-O(2)-H(2)	109.5

Symmetry transformations used to generate equivalent atoms:

Table 4. Anisotropic displacement parameters ($Å^2$x 10^3) for sarpong38. The anisotropic displacement factor exponent takes the form: $-2\pi^2$[h^2 $a^{*2}U^{11}$ + ... + 2 h k a* b* U^{12}]

	U^{11}	U^{22}	U^{33}	U^{23}	U^{13}	U^{12}
C(1)	19(1)	22(1)	23(1)	-2(1)	3(1)	-1(1)
C(2)	21(1)	29(2)	27(1)	0(1)	5(1)	-4(1)
C(3)	26(1)	41(2)	25(1)	3(1)	6(1)	-2(1)
C(4)	24(1)	35(2)	22(1)	2(1)	2(1)	-3(1)
C(5)	20(1)	29(2)	21(1)	3(1)	4(1)	2(1)
C(6)	18(1)	23(1)	22(1)	-1(1)	3(1)	-4(1)
C(7)	19(1)	24(1)	28(1)	4(1)	4(1)	-1(1)
C(8)	25(1)	24(1)	28(1)	5(1)	8(1)	3(1)
C(9)	27(1)	32(2)	28(1)	9(1)	10(1)	5(1)
C(10)	42(2)	30(2)	25(1)	2(1)	10(1)	3(1)
C(11)	37(1)	26(2)	21(1)	-2(1)	4(1)	-8(1)
C(12)	22(1)	18(1)	24(1)	-1(1)	3(1)	-2(1)
C(13)	22(1)	28(2)	29(1)	-2(1)	5(1)	-1(1)
C(14)	39(2)	22(2)	30(1)	4(1)	9(1)	4(1)
C(15)	22(1)	42(2)	30(1)	7(1)	5(1)	5(1)
N(1)	26(1)	21(1)	23(1)	2(1)	4(1)	-3(1)
N(2)	37(1)	31(2)	52(2)	-9(1)	9(1)	0(1)
O(1)	34(1)	64(2)	30(1)	17(1)	5(1)	9(1)
O(2)	30(1)	26(1)	40(1)	12(1)	-6(1)	-3(1)
O(3)	21(1)	42(1)	25(1)	-2(1)	1(1)	-7(1)

Table 5. Hydrogen coordinates (x 10^4) and isotropic displacement parameters ($Å^2$x 10^3) for sarpong38.

	x	y	z	U(eq)
H(2A)	4373	11907	6877	30
H(2B)	5247	10153	6368	30
H(3A)	3418	11742	5042	36
H(3B)	2693	9645	5179	36
H(6)	1032	8411	6583	25
H(7A)	-412	8551	8067	28
H(7B)	1162	7106	8185	28
H(8)	1077	10742	9164	30
H(9)	307	8590	10353	35
H(10A)	3185	8555	11534	38
H(10B)	2396	10597	11162	38
H(11A)	4860	10961	10479	33
H(11B)	5175	8696	10436	33
H(14A)	848	14182	7114	45
H(14B)	2412	12989	7713	45
H(14C)	602	12938	8117	45
H(15A)	-1710	10955	7279	47
H(15B)	-1656	10060	6147	47
H(15C)	-1785	12317	6282	47
H(2)	2546	6065	10482	49

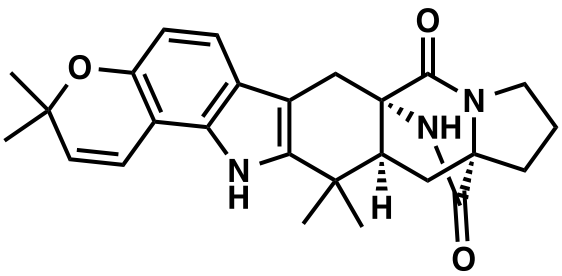

(+)-stephacidin A (1)

A colorless plate 0.060 x 0.040 x 0.020 mm in size was mounted on a Cryoloop with Paratone oil. Data were collected in a nitrogen gas stream at 100(2) K using phi and omega scans. Crystal-to-detector distance was 60 mm and exposure time was 10 seconds per frame using a scan width of 1.0°. Data collection was 97.8% complete to 67.000° in θ. A total of 39292 reflections were collected covering the indices, -10<=*h*<=10, -10<=*k*<=8, -42<=*l*<=42. 5366 reflections were found to be symmetry independent, with an R_{int} of 0.0438. Indexing and unit cell refinement indicated a primitive, orthorhombic lattice. The space group was found to be P 21 21 21 (No. 19). The data were integrated using the Bruker SAINT software program and scaled using the SADABS software program. Solution by iterative methods (SHELXT) produced a complete heavy-atom phasing model consistent with the proposed structure. All non-hydrogen atoms were refined anisotropically by full-matrix least-squares (SHELXL-2014). All hydrogen atoms were placed using a riding model. Their positions were constrained relative to their parent atom using the appropriate HFIX command in SHELXL-2014. CCDC # 1400756 (**1**) contains the supplementary crystallographic data for this paper. These data can be obtained free of charge from The Cambridge Crystallographic Data Centre via www.ccdc.cam.ac.uk/data_request/cif.

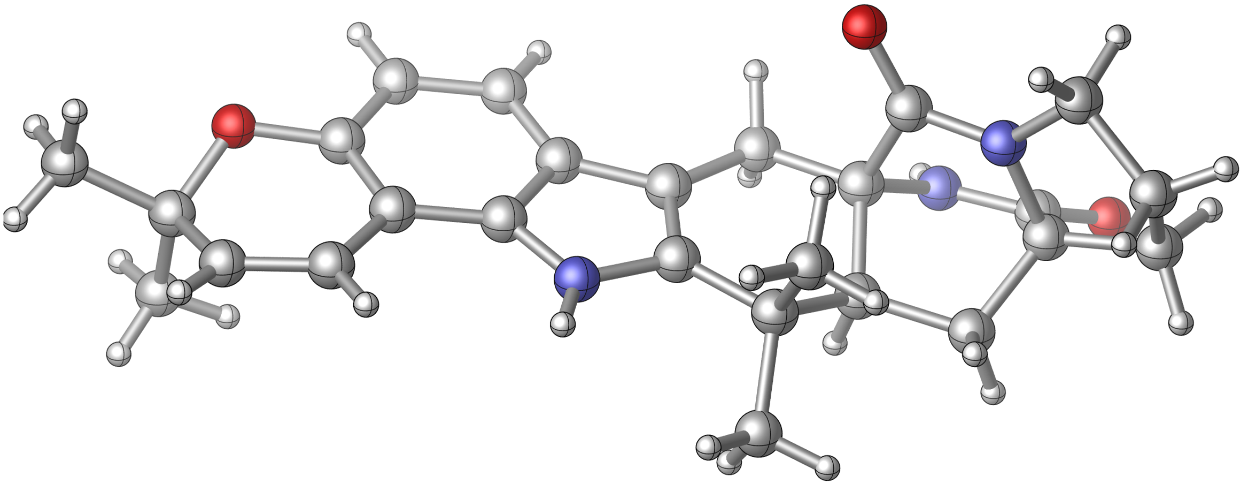

CYLview of (+)-stephacidin A (**1**)

Table 1. Crystal data and structure refinement for sarpong107.

X-ray ID	sarpong107	
Sample/notebook ID	EM07-137C	
Empirical formula	C26 H29 N3 O3	
Formula weight	431.52	
Temperature	100(2) K	
Wavelength	1.54178 Å	
Crystal system	Orthorhombic	
Space group	P 21 21 21	
Unit cell dimensions	a = 8.7961(4) Å	α= 90°.
	b = 9.7026(5) Å	β= 90°.
	c = 35.0914(18) Å	γ = 90°.
Volume	2994.9(3) $Å^3$	
Z	4	
Density (calculated)	0.957 Mg/m^3	
Absorption coefficient	0.506 mm^{-1}	
F(000)	920	
Crystal size	0.060 x 0.040 x 0.020 mm^3	
Theta range for data collection	2.518 to 68.506°.	
Index ranges	-10<=h<=10, -10<=k<=8, -42<=l<=42	
Reflections collected	39292	
Independent reflections	5366 [R(int) = 0.0438]	
Completeness to theta = 67.000°	97.8 %	
Absorption correction	Semi-empirical from equivalents	
Max. and min. transmission	0.929 and 0.777	
Refinement method	Full-matrix least-squares on F^2	
Data / restraints / parameters	5366 / 0 / 293	
Goodness-of-fit on F^2	1.055	
Final R indices [I>2sigma(I)]	R1 = 0.0628, wR2 = 0.1563	
R indices (all data)	R1 = 0.0660, wR2 = 0.1589	
Absolute structure parameter	-1.08(14)	
Extinction coefficient	n/a	
Largest diff. peak and hole	0.388 and -0.265 e.$Å^{-3}$	

Table 2. Atomic coordinates (x 10^4) and equivalent isotropic displacement parameters ($Å^2$x 10^3) for sarpong107. U(eq) is defined as one third of the trace of the orthogonalized U^{ij} tensor.

	x	y	z	U(eq)
C(1)	5760(5)	2190(5)	3456(1)	32(1)
C(2)	7122(5)	1545(5)	3268(1)	32(1)
C(3)	7302(4)	2022(5)	2870(1)	29(1)
C(4)	8333(4)	1546(4)	2581(1)	26(1)
C(5)	9424(4)	478(5)	2570(1)	29(1)
C(6)	10229(4)	243(5)	2235(1)	33(1)
C(7)	9971(5)	1075(5)	1914(1)	33(1)
C(8)	11049(6)	1892(5)	1329(1)	42(1)
C(9)	9579(6)	2719(6)	1266(1)	50(1)
C(10)	8620(6)	2868(6)	1563(1)	48(1)
C(11)	8870(5)	2119(5)	1908(1)	35(1)
C(12)	8070(4)	2348(5)	2251(1)	31(1)
C(13)	6487(4)	3075(5)	2711(1)	28(1)
C(14)	5288(5)	3920(4)	2893(1)	30(1)
C(15)	5500(5)	3696(5)	3335(1)	33(1)
C(16)	4178(5)	4330(5)	3572(1)	39(1)
C(17)	3510(5)	3176(5)	3824(1)	39(1)
C(18)	2001(6)	3495(6)	4021(2)	52(1)
C(19)	826(6)	2947(7)	3742(2)	54(1)
C(20)	1515(5)	1648(6)	3588(2)	48(1)
C(21)	4271(5)	1387(5)	3393(1)	31(1)
C(22)	4808(5)	2694(6)	4089(1)	41(1)
C(23)	12315(7)	2795(6)	1469(2)	55(1)
C(24)	11487(7)	1178(6)	956(2)	54(1)
C(25)	5505(5)	5463(5)	2811(1)	38(1)
C(26)	3708(5)	3500(5)	2737(1)	34(1)
N(1)	6948(4)	3272(4)	2337(1)	29(1)
N(2)	3155(4)	1987(4)	3581(1)	34(1)
N(3)	5959(4)	2220(4)	3876(1)	38(1)
O(1)	10765(4)	780(3)	1584(1)	39(1)
O(2)	4164(4)	338(3)	3194(1)	38(1)

O(3)	4792(4)	2780(4)	4434(1)	54(1)

Table 3. Bond lengths [Å] and angles [°] for sarpong107.

C(1)-N(3)	1.483(5)	C(16)-C(17)	1.541(7)
C(1)-C(2)	1.504(6)	C(16)-H(16A)	0.9900
C(1)-C(15)	1.539(7)	C(16)-H(16B)	0.9900
C(1)-C(21)	1.540(6)	C(17)-N(2)	1.468(6)
C(2)-C(3)	1.482(6)	C(17)-C(18)	1.529(6)
C(2)-H(2A)	0.9900	C(17)-C(22)	1.545(7)
C(2)-H(2B)	0.9900	C(18)-C(19)	1.519(8)
C(3)-C(13)	1.367(6)	C(18)-H(18A)	0.9900
C(3)-C(4)	1.435(6)	C(18)-H(18B)	0.9900
C(4)-C(5)	1.413(6)	C(19)-C(20)	1.499(8)
C(4)-C(12)	1.416(6)	C(19)-H(19A)	0.9900
C(5)-C(6)	1.391(6)	C(19)-H(19B)	0.9900
C(5)-H(5)	0.9500	C(20)-N(2)	1.480(6)
C(6)-C(7)	1.404(6)	C(20)-H(20A)	0.9900
C(6)-H(6)	0.9500	C(20)-H(20B)	0.9900
C(7)-O(1)	1.382(5)	C(21)-O(2)	1.237(5)
C(7)-C(11)	1.402(6)	C(21)-N(2)	1.318(6)
C(8)-O(1)	1.423(6)	C(22)-O(3)	1.214(5)
C(8)-C(23)	1.500(8)	C(22)-N(3)	1.341(6)
C(8)-C(24)	1.532(7)	C(23)-H(23A)	0.9800
C(8)-C(9)	1.538(7)	C(23)-H(23B)	0.9800
C(9)-C(10)	1.349(6)	C(23)-H(23C)	0.9800
C(9)-H(9)	0.9500	C(24)-H(24A)	0.9800
C(10)-C(11)	1.428(7)	C(24)-H(24B)	0.9800
C(10)-H(10)	0.9500	C(24)-H(24C)	0.9800
C(11)-C(12)	1.411(6)	C(25)-H(25A)	0.9800
C(12)-N(1)	1.367(5)	C(25)-H(25B)	0.9800
C(13)-N(1)	1.386(5)	C(25)-H(25C)	0.9800
C(13)-C(14)	1.481(6)	C(26)-H(26A)	0.9800
C(14)-C(25)	1.536(7)	C(26)-H(26B)	0.9800
C(14)-C(26)	1.548(6)	C(26)-H(26C)	0.9800
C(14)-C(15)	1.578(6)	N(1)-H(1)	0.8800
C(15)-C(16)	1.557(6)	N(3)-H(3)	0.8800
C(15)-H(15)	1.0000		

N(3)-C(1)-C(2)	110.4(3)	C(8)-C(9)-H(9)	121.0
N(3)-C(1)-C(15)	105.9(4)	C(9)-C(10)-C(11)	120.2(5)
C(2)-C(1)-C(15)	113.1(4)	C(9)-C(10)-H(10)	119.9
N(3)-C(1)-C(21)	104.7(3)	C(11)-C(10)-H(10)	119.9
C(2)-C(1)-C(21)	113.8(4)	C(7)-C(11)-C(12)	116.4(4)
C(15)-C(1)-C(21)	108.3(3)	C(7)-C(11)-C(10)	119.2(4)
C(3)-C(2)-C(1)	111.7(4)	C(12)-C(11)-C(10)	124.4(4)
C(3)-C(2)-H(2A)	109.3	N(1)-C(12)-C(11)	130.7(4)
C(1)-C(2)-H(2A)	109.3	N(1)-C(12)-C(4)	107.2(4)
C(3)-C(2)-H(2B)	109.3	C(11)-C(12)-C(4)	122.1(4)
C(1)-C(2)-H(2B)	109.3	C(3)-C(13)-N(1)	109.6(4)
H(2A)-C(2)-H(2B)	107.9	C(3)-C(13)-C(14)	127.7(4)
C(13)-C(3)-C(4)	106.5(4)	N(1)-C(13)-C(14)	122.7(4)
C(13)-C(3)-C(2)	124.2(4)	C(13)-C(14)-C(25)	111.8(4)
C(4)-C(3)-C(2)	129.3(4)	C(13)-C(14)-C(26)	110.0(3)
C(5)-C(4)-C(12)	119.3(4)	C(25)-C(14)-C(26)	107.6(4)
C(5)-C(4)-C(3)	133.3(4)	C(13)-C(14)-C(15)	105.3(4)
C(12)-C(4)-C(3)	107.3(4)	C(25)-C(14)-C(15)	107.6(4)
C(6)-C(5)-C(4)	119.4(4)	C(26)-C(14)-C(15)	114.7(3)
C(6)-C(5)-H(5)	120.3	C(1)-C(15)-C(16)	109.7(4)
C(4)-C(5)-H(5)	120.3	C(1)-C(15)-C(14)	114.8(3)
C(5)-C(6)-C(7)	120.0(4)	C(16)-C(15)-C(14)	112.6(4)
C(5)-C(6)-H(6)	120.0	C(1)-C(15)-H(15)	106.4
C(7)-C(6)-H(6)	120.0	C(16)-C(15)-H(15)	106.4
O(1)-C(7)-C(11)	119.1(4)	C(14)-C(15)-H(15)	106.4
O(1)-C(7)-C(6)	118.1(4)	C(17)-C(16)-C(15)	107.7(4)
C(11)-C(7)-C(6)	122.7(4)	C(17)-C(16)-H(16A)	110.2
O(1)-C(8)-C(23)	111.6(4)	C(15)-C(16)-H(16A)	110.2
O(1)-C(8)-C(24)	103.8(4)	C(17)-C(16)-H(16B)	110.2
C(23)-C(8)-C(24)	110.9(4)	C(15)-C(16)-H(16B)	110.2
O(1)-C(8)-C(9)	109.8(4)	H(16A)-C(16)-H(16B)	108.5
C(23)-C(8)-C(9)	111.5(5)	N(2)-C(17)-C(18)	103.7(4)
C(24)-C(8)-C(9)	108.9(4)	N(2)-C(17)-C(16)	108.6(4)
C(10)-C(9)-C(8)	118.1(4)	C(18)-C(17)-C(16)	116.3(4)
C(10)-C(9)-H(9)	121.0	N(2)-C(17)-C(22)	105.6(4)

C(18)-C(17)-C(22)	115.5(4)
C(16)-C(17)-C(22)	106.4(4)
C(19)-C(18)-C(17)	103.2(4)
C(19)-C(18)-H(18A)	111.1
C(17)-C(18)-H(18A)	111.1
C(19)-C(18)-H(18B)	111.1
C(17)-C(18)-H(18B)	111.1
H(18A)-C(18)-H(18B)	109.1
C(20)-C(19)-C(18)	104.6(4)
C(20)-C(19)-H(19A)	110.8
C(18)-C(19)-H(19A)	110.8
C(20)-C(19)-H(19B)	110.8
C(18)-C(19)-H(19B)	110.8
H(19A)-C(19)-H(19B)	108.9
N(2)-C(20)-C(19)	102.3(4)
N(2)-C(20)-H(20A)	111.3
C(19)-C(20)-H(20A)	111.3
N(2)-C(20)-H(20B)	111.3
C(19)-C(20)-H(20B)	111.3
H(20A)-C(20)-H(20B)	109.2
O(2)-C(21)-N(2)	126.1(4)
O(2)-C(21)-C(1)	124.2(4)
N(2)-C(21)-C(1)	109.7(4)
O(3)-C(22)-N(3)	126.1(5)
O(3)-C(22)-C(17)	124.8(4)
N(3)-C(22)-C(17)	109.1(4)
C(8)-C(23)-H(23A)	109.5
C(8)-C(23)-H(23B)	109.5
H(23A)-C(23)-H(23B)	109.5
C(8)-C(23)-H(23C)	109.5
H(23A)-C(23)-H(23C)	109.5
H(23B)-C(23)-H(23C)	109.5
C(8)-C(24)-H(24A)	109.5
C(8)-C(24)-H(24B)	109.5
H(24A)-C(24)-H(24B)	109.5
C(8)-C(24)-H(24C)	109.5
H(24A)-C(24)-H(24C)	109.5
H(24B)-C(24)-H(24C)	109.5
C(14)-C(25)-H(25A)	109.5
C(14)-C(25)-H(25B)	109.5
H(25A)-C(25)-H(25B)	109.5
C(14)-C(25)-H(25C)	109.5
H(25A)-C(25)-H(25C)	109.5
H(25B)-C(25)-H(25C)	109.5
C(14)-C(26)-H(26A)	109.5
C(14)-C(26)-H(26B)	109.5
H(26A)-C(26)-H(26B)	109.5
C(14)-C(26)-H(26C)	109.5
H(26A)-C(26)-H(26C)	109.5
H(26B)-C(26)-H(26C)	109.5
C(12)-N(1)-C(13)	109.3(3)
C(12)-N(1)-H(1)	125.3
C(13)-N(1)-H(1)	125.3
C(21)-N(2)-C(17)	118.7(4)
C(21)-N(2)-C(20)	129.5(4)
C(17)-N(2)-C(20)	111.9(4)
C(22)-N(3)-C(1)	118.1(4)
C(22)-N(3)-H(3)	121.0
C(1)-N(3)-H(3)	121.0
C(7)-O(1)-C(8)	117.3(3)

Symmetry transformations used to generate equivalent atoms:

Table 4. Anisotropic displacement parameters ($Å^2$x 10^3)for sarpong107. The anisotropic displacement factor exponent takes the form: $-2\pi^2[h^2a^{*2}U^{11} + ... + 2 h k a^* b^* U^{12}]$

	U^{11}	U^{22}	U^{33}	U^{23}	U^{13}	U^{12}
C(1)	26(2)	34(2)	35(2)	1(2)	2(2)	-4(2)
C(2)	28(2)	36(3)	31(2)	4(2)	-1(2)	4(2)
C(3)	19(2)	35(2)	34(2)	-4(2)	-2(2)	-1(2)
C(4)	29(2)	17(2)	31(2)	1(2)	-1(2)	-3(2)
C(5)	16(2)	37(3)	34(2)	2(2)	-1(2)	3(2)
C(6)	22(2)	35(3)	43(2)	-2(2)	5(2)	5(2)
C(7)	23(2)	32(3)	44(2)	0(2)	4(2)	0(2)
C(8)	45(3)	37(3)	43(3)	-3(2)	10(2)	5(2)
C(9)	64(3)	59(4)	29(2)	12(2)	9(2)	21(3)
C(10)	41(3)	57(3)	44(3)	3(2)	13(2)	9(3)
C(11)	28(2)	36(3)	40(2)	-1(2)	3(2)	-9(2)
C(12)	24(2)	34(3)	36(2)	-4(2)	-3(2)	-4(2)
C(13)	23(2)	27(2)	36(2)	-1(2)	6(2)	-3(2)
C(14)	30(2)	21(2)	40(2)	0(2)	4(2)	-3(2)
C(15)	29(2)	32(3)	38(2)	-8(2)	-3(2)	-2(2)
C(16)	37(2)	39(3)	42(2)	-9(2)	7(2)	3(2)
C(17)	37(2)	40(3)	38(2)	-5(2)	6(2)	1(2)
C(18)	38(3)	59(4)	59(3)	1(3)	18(2)	1(2)
C(19)	32(2)	70(4)	60(3)	-8(3)	13(2)	13(3)
C(20)	27(2)	59(4)	58(3)	-1(3)	3(2)	3(2)
C(21)	31(2)	37(3)	25(2)	9(2)	3(2)	-2(2)
C(22)	37(2)	49(3)	37(2)	-11(2)	6(2)	-2(2)
C(23)	69(4)	49(3)	47(3)	-4(2)	6(3)	-9(3)
C(24)	57(3)	58(4)	47(3)	-2(3)	18(3)	6(3)
C(25)	29(2)	40(3)	44(2)	-1(2)	-1(2)	10(2)
C(26)	28(2)	39(3)	35(2)	-1(2)	6(2)	1(2)
N(1)	25(2)	33(2)	29(2)	2(1)	-1(1)	5(2)
N(2)	26(2)	39(2)	37(2)	2(2)	2(2)	-2(2)
N(3)	27(2)	50(3)	38(2)	-5(2)	-4(2)	1(2)
O(1)	36(2)	39(2)	41(2)	-2(1)	14(1)	2(1)
O(2)	42(2)	35(2)	36(2)	-7(1)	3(1)	-3(2)

O(3)	58(2)	79(3)	26(2)	-6(2)	6(2)	7(2)

Table 5. Hydrogen coordinates (x 10^4) and isotropic displacement parameters (Å^{2}x 10^3) for sarpong107.

	x	y	z	U(eq)
H(2A)	8047	1782	3415	38
H(2B)	7010	530	3271	38
H(5)	9606	-74	2788	35
H(6)	10955	-481	2223	40
H(9)	9354	3114	1025	60
H(10)	7775	3474	1543	57
H(15)	6439	4213	3408	40
H(16A)	4563	5091	3734	47
H(16B)	3385	4702	3401	47
H(18A)	1928	3019	4269	63
H(18B)	1875	4499	4061	63
H(19A)	-147	2751	3873	65
H(19B)	639	3618	3535	65
H(20A)	1308	854	3757	58
H(20B)	1130	1440	3329	58
H(23A)	12067	3141	1724	83
H(23B)	12446	3574	1294	83
H(23C)	13259	2260	1480	83
H(24A)	12372	585	999	81
H(24B)	11736	1874	764	81
H(24C)	10632	617	866	81
H(25A)	6542	5736	2880	56
H(25B)	4776	5997	2962	56
H(25C)	5337	5640	2540	56
H(26A)	3682	3645	2461	51
H(26B)	2922	4064	2859	51
H(26C)	3522	2525	2793	51
H(1)	6578	3895	2180	35
H(3)	6809	1934	3982	46

^{1}H NMR (600 MHz, $CDCl_3$)

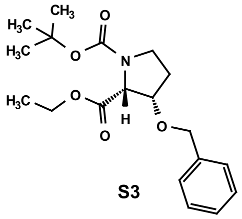

S3

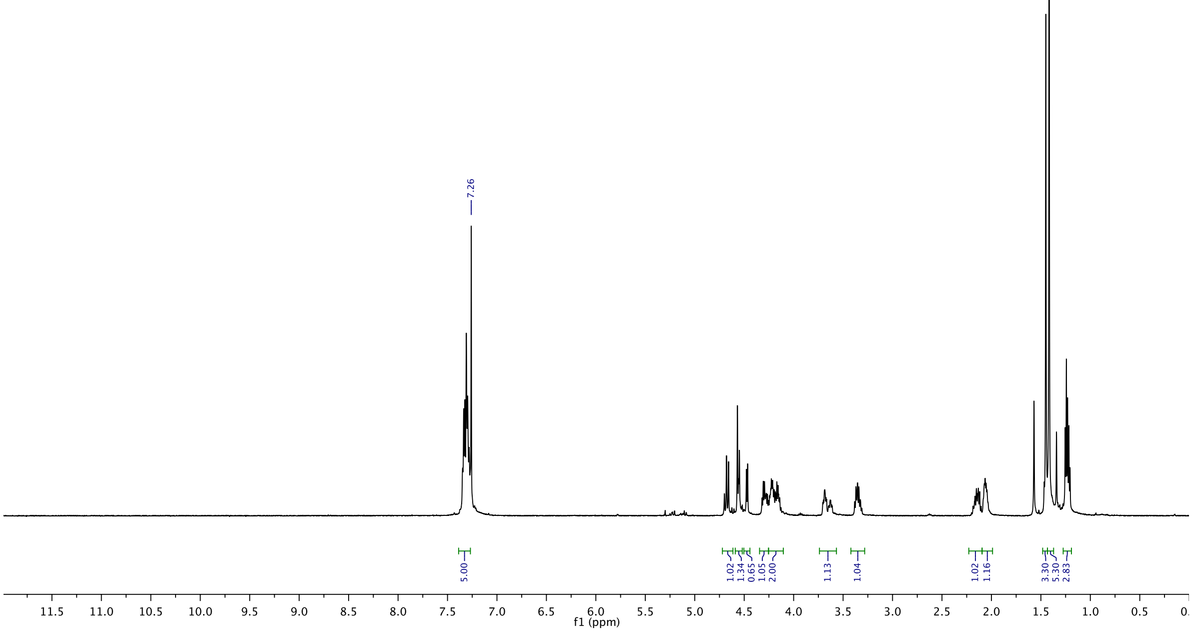

^{13}C NMR (150 MHz, $CDCl_3$)

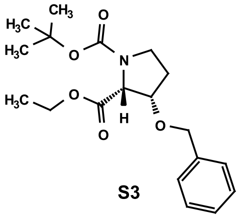

S3

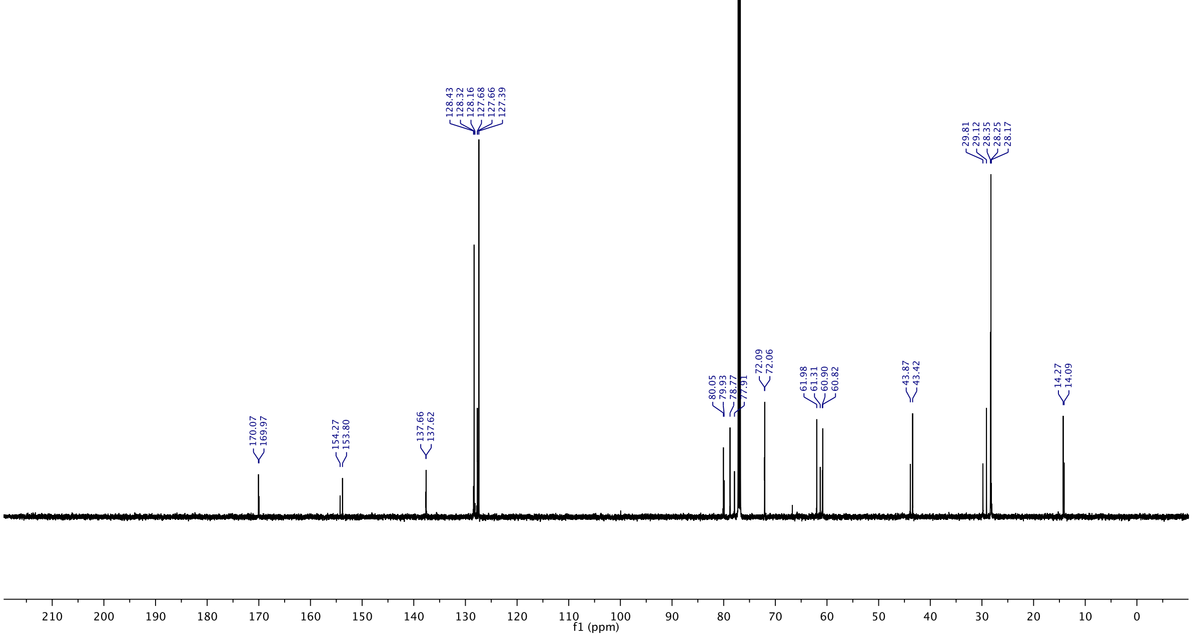

^{1}H NMR (600 MHz, $CDCl_3$)

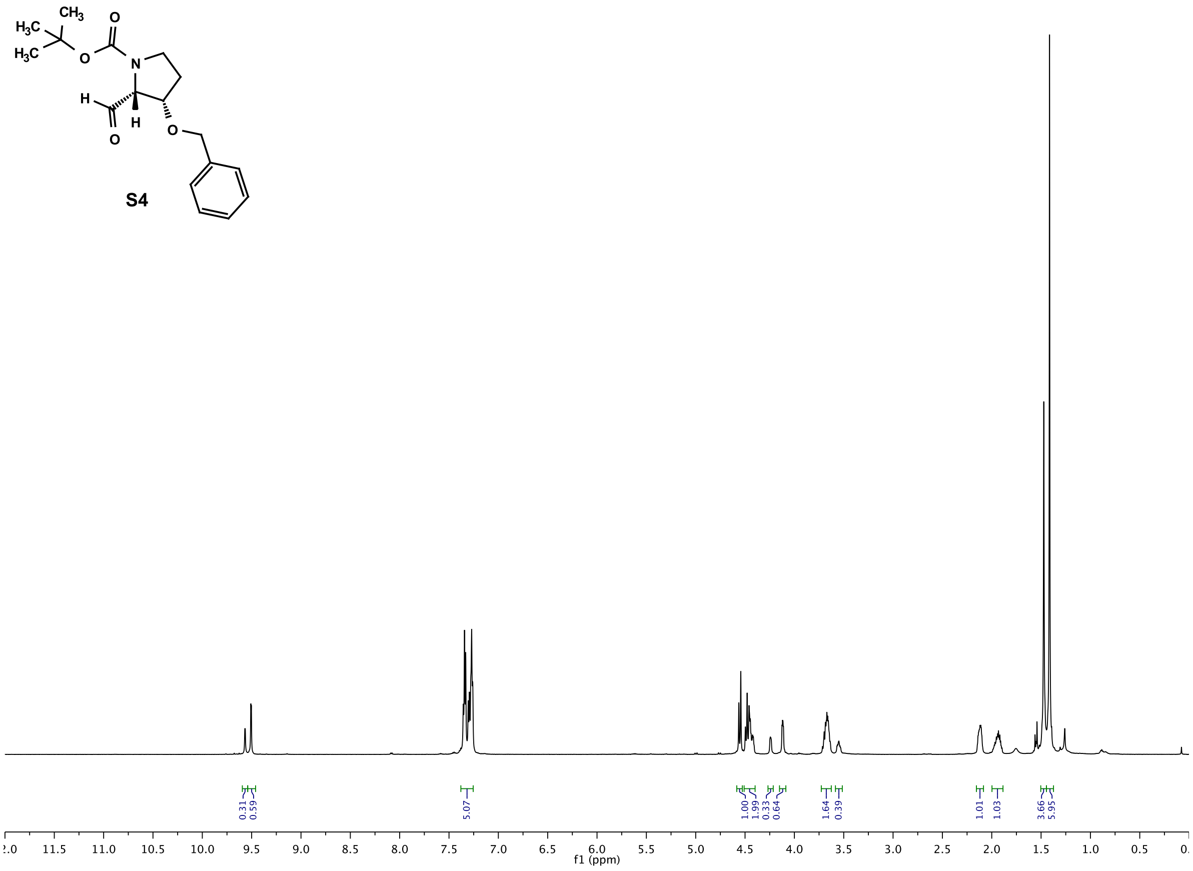

^{13}C NMR (150 MHz, $CDCl_3$)

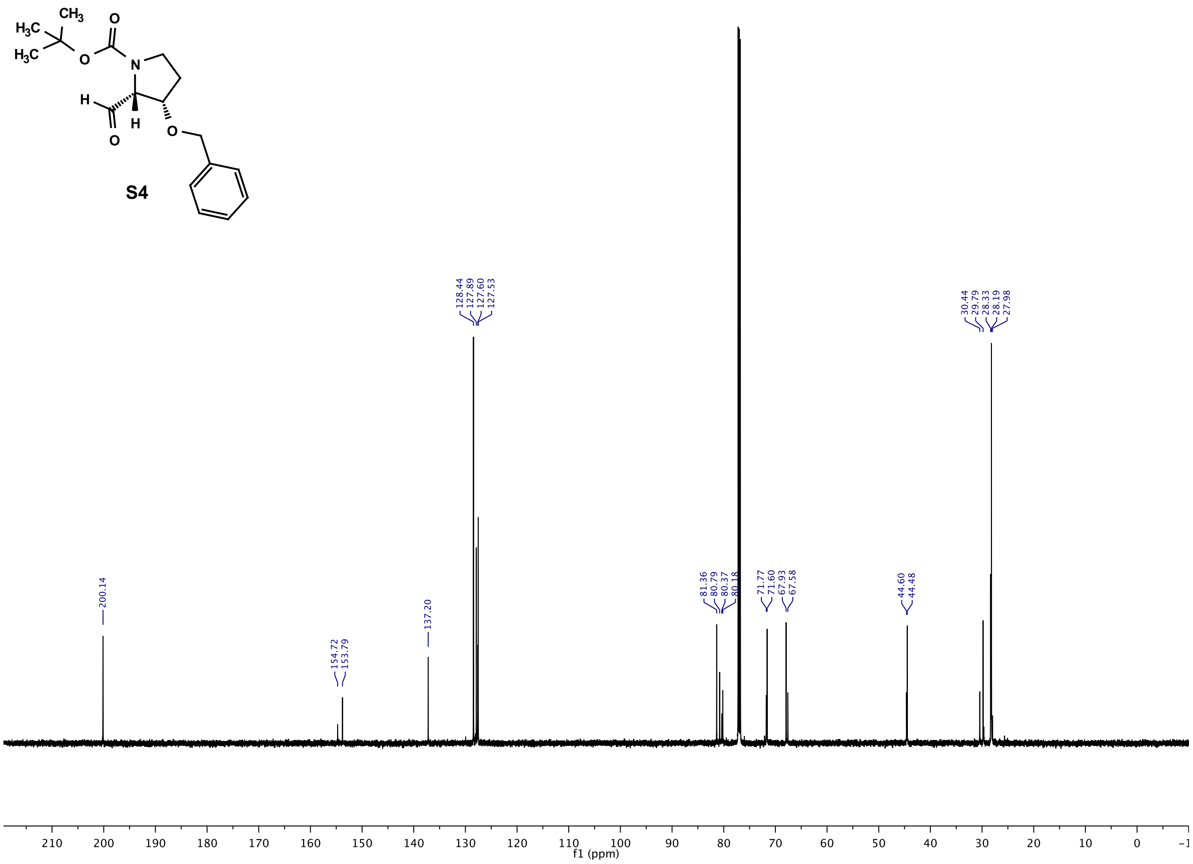

^1H NMR (600 MHz, $CDCl_3$)

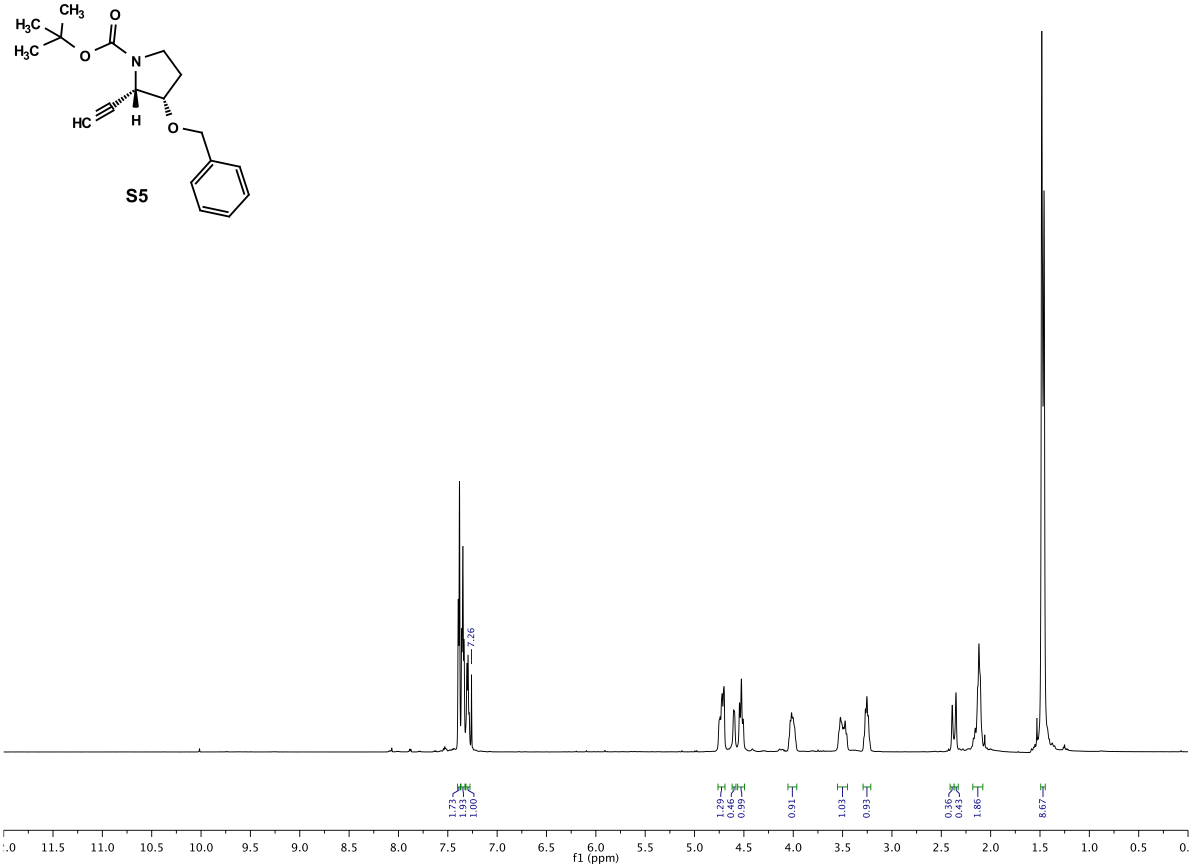

S5

^{13}C NMR (150 MHz, $CDCl_3$)

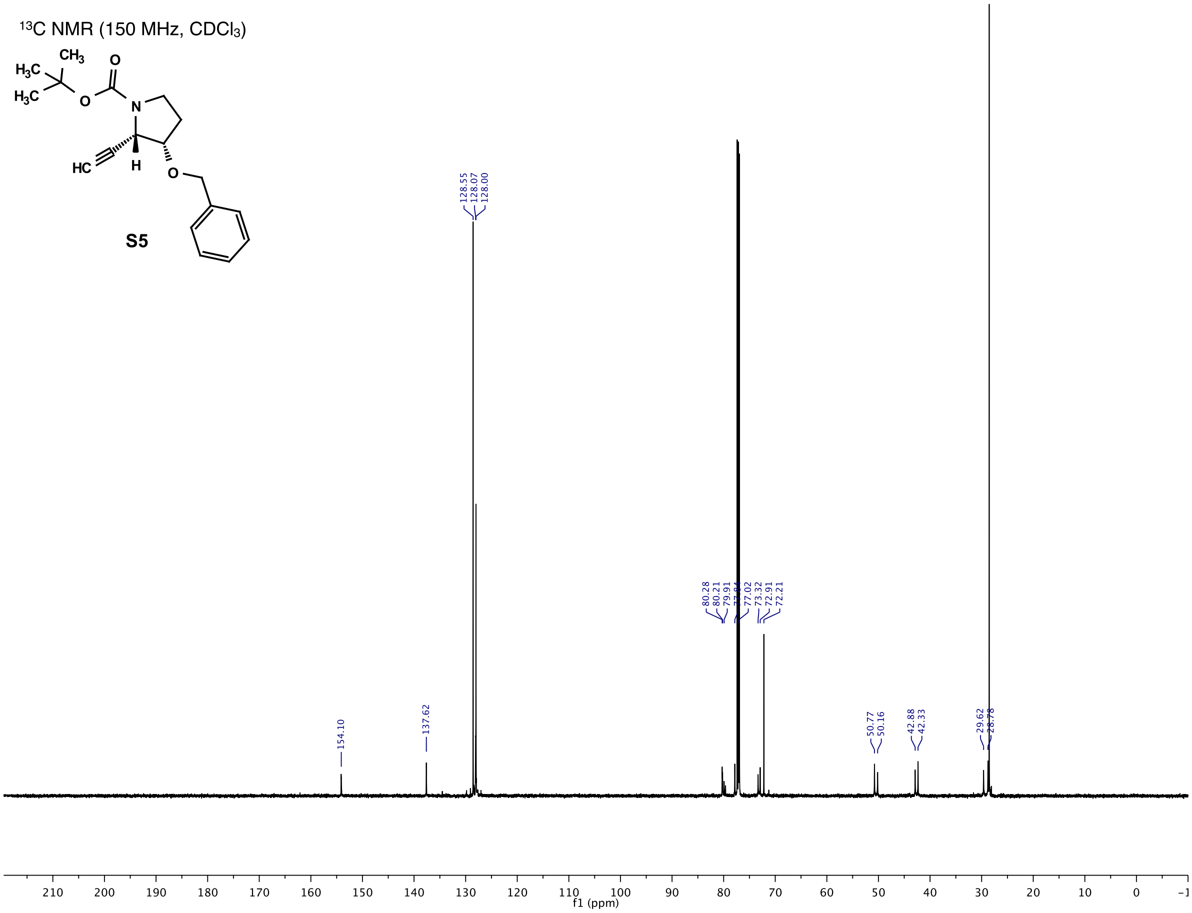

S5

^{1}H NMR (600 MHz, $CDCl_3$)

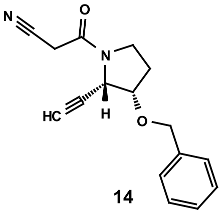

14

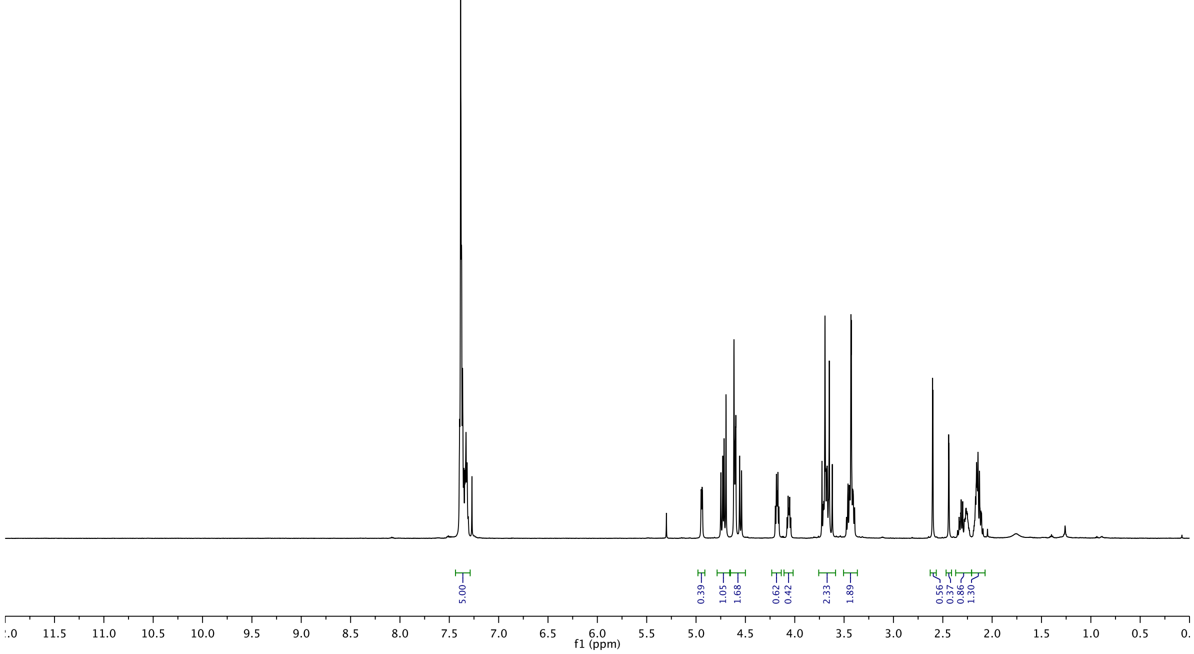

^{13}C NMR (150 MHz, $CDCl_3$)

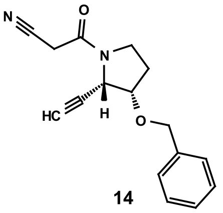

14

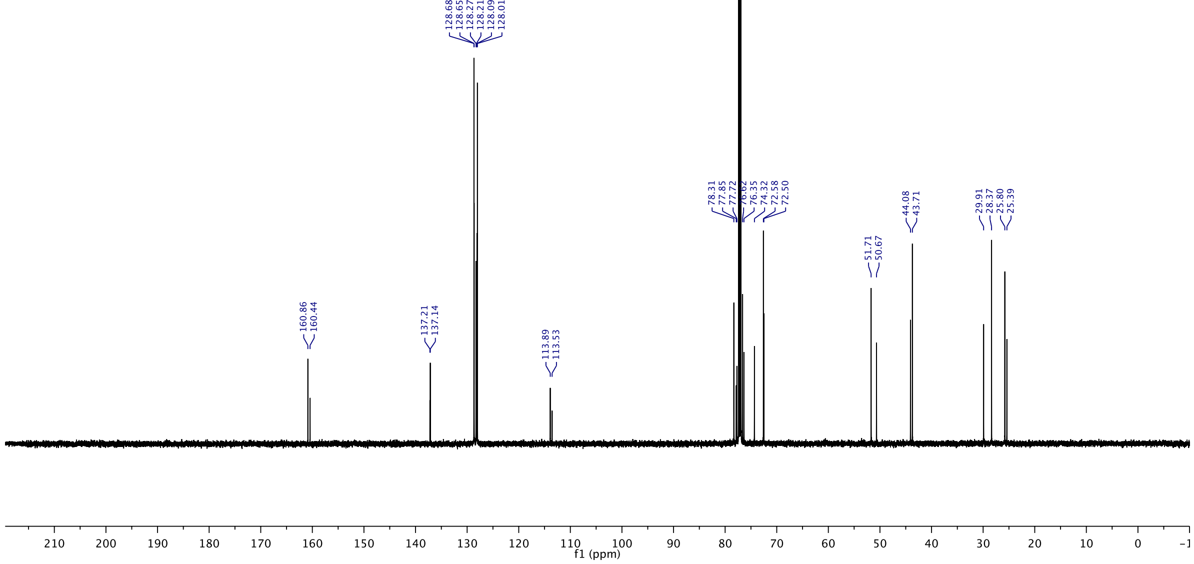

¹H NMR (600 MHz, $CDCl_3$)

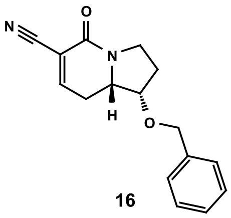

16

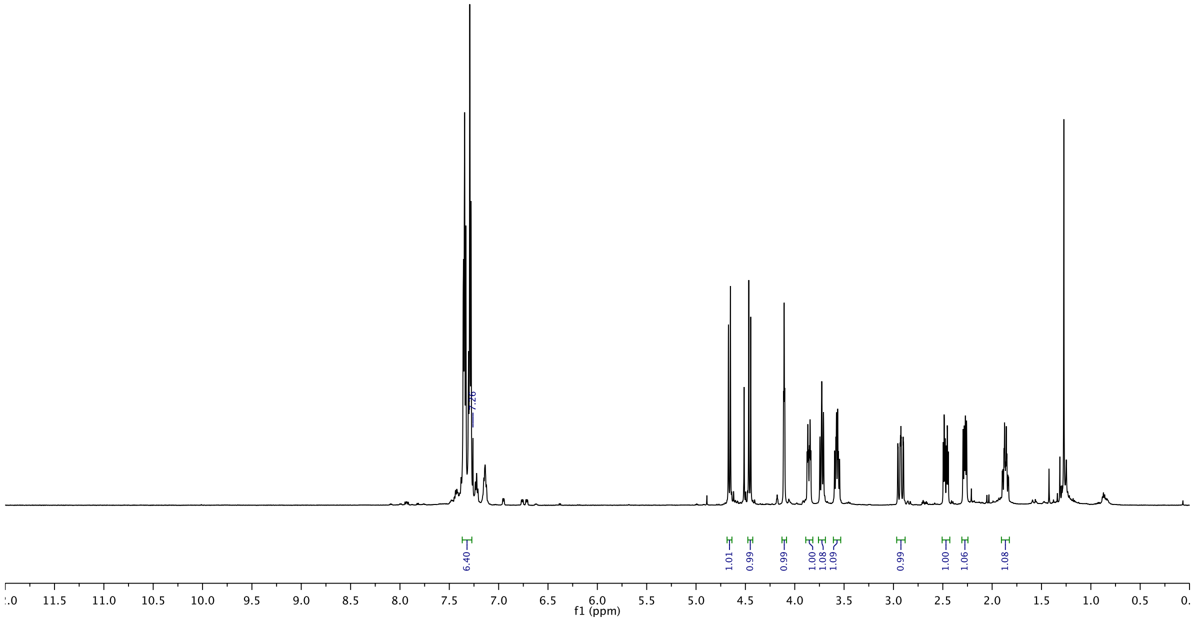

¹³C NMR (150 MHz, $CDCl_3$)

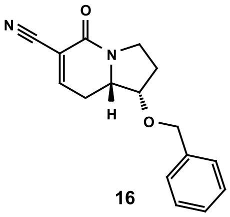

16

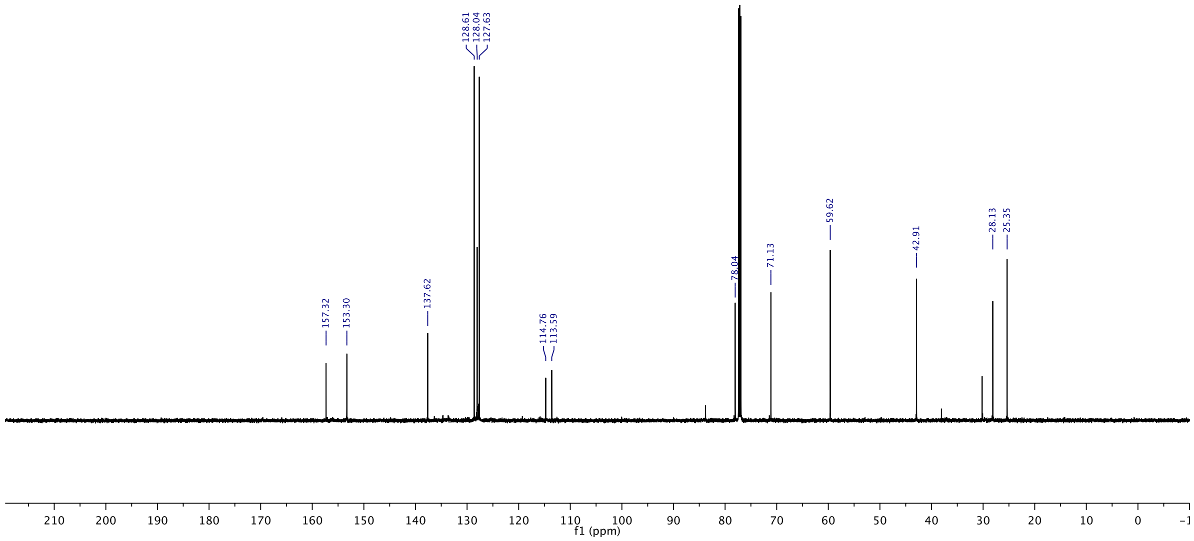

^1H NMR (600 MHz, $CDCl_3$)

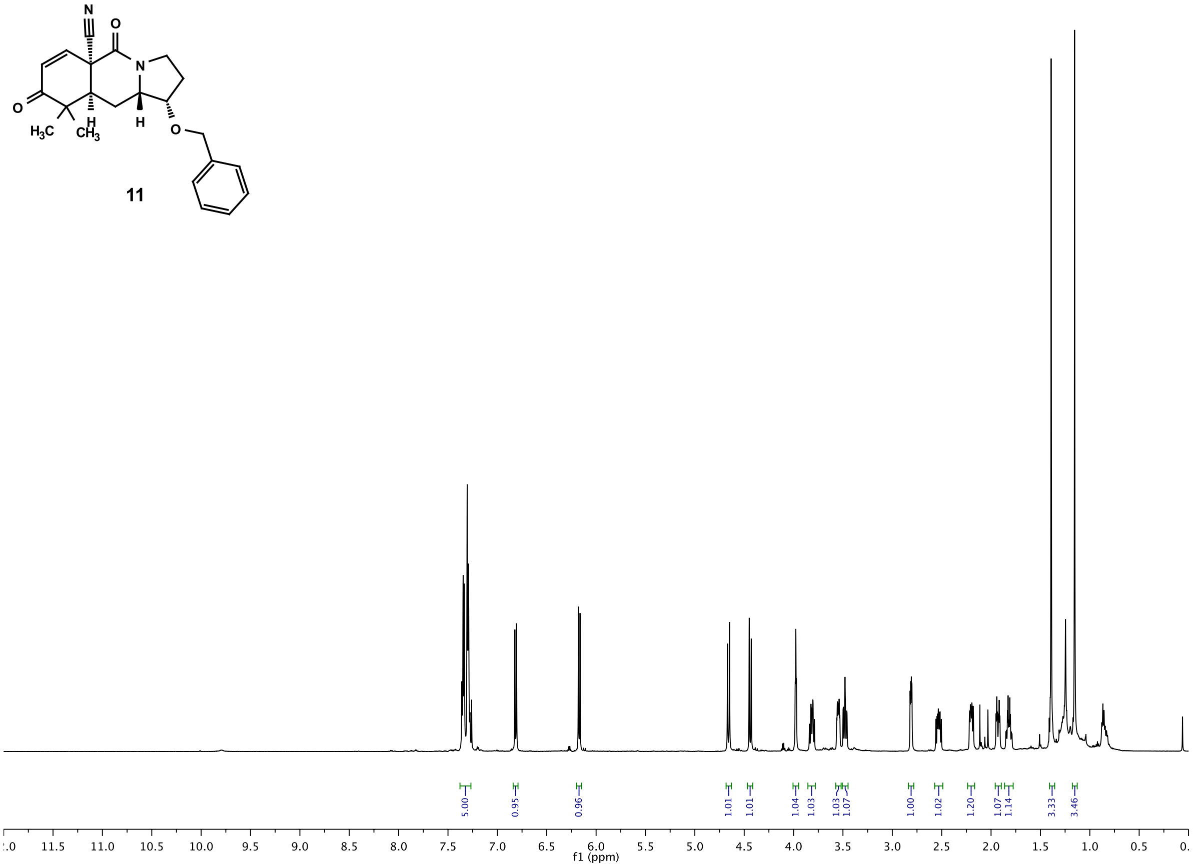

^{13}C NMR (150 MHz, $CDCl_3$)

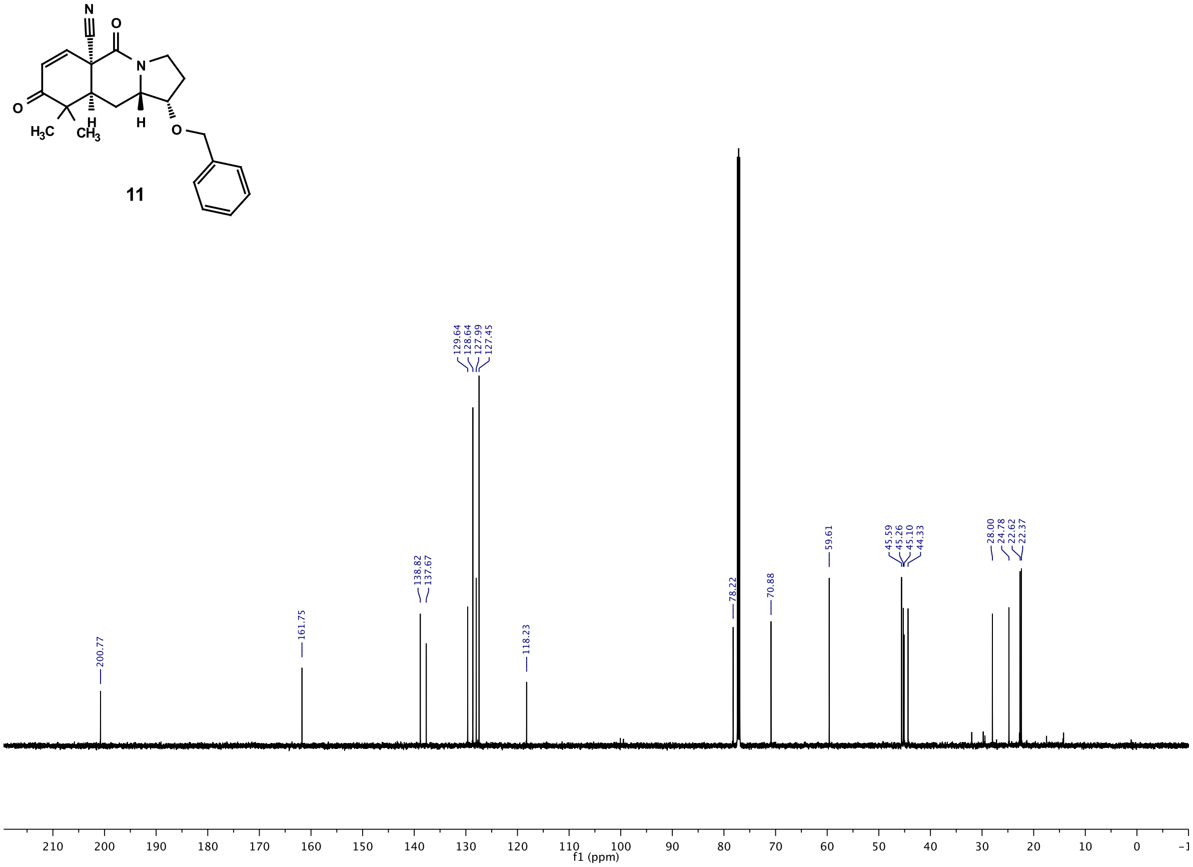

^{1}H NMR (600 MHz, $CDCl_3$)

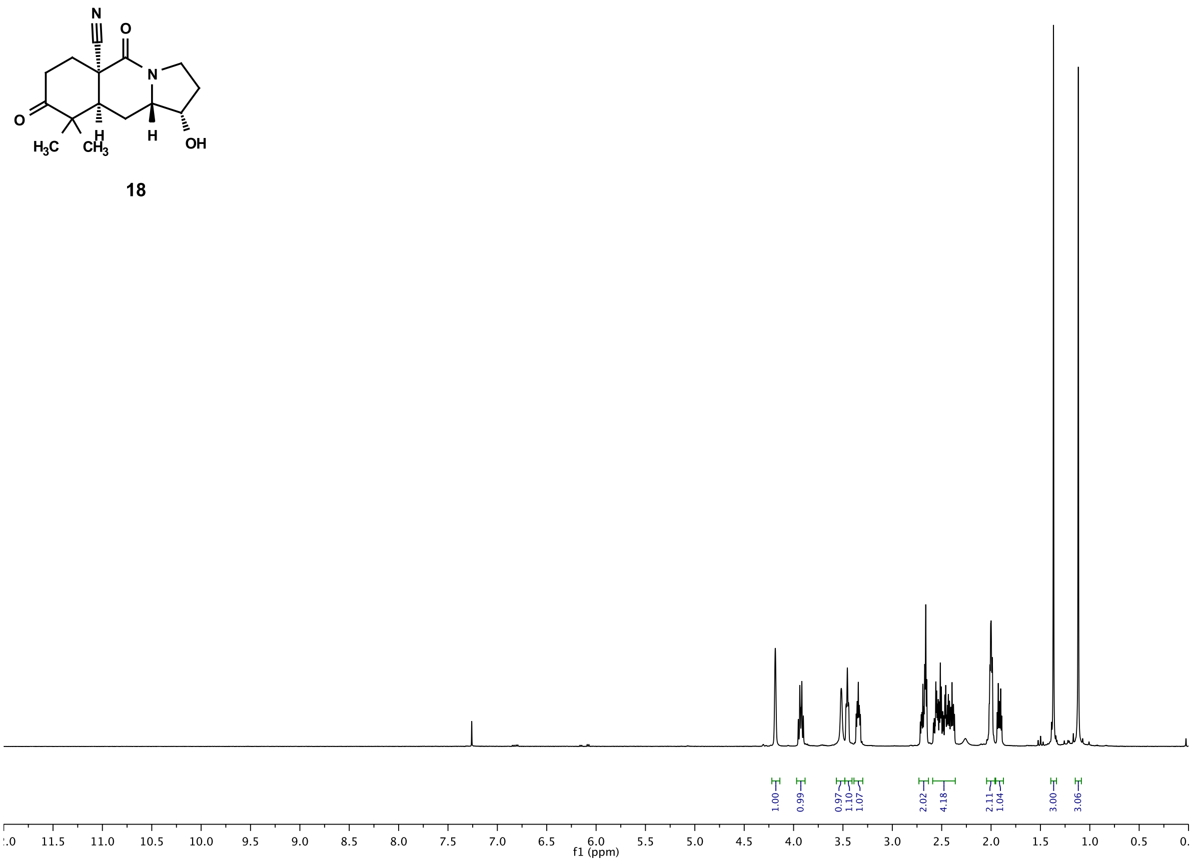

^{13}C NMR (150 MHz, $CDCl_3$)

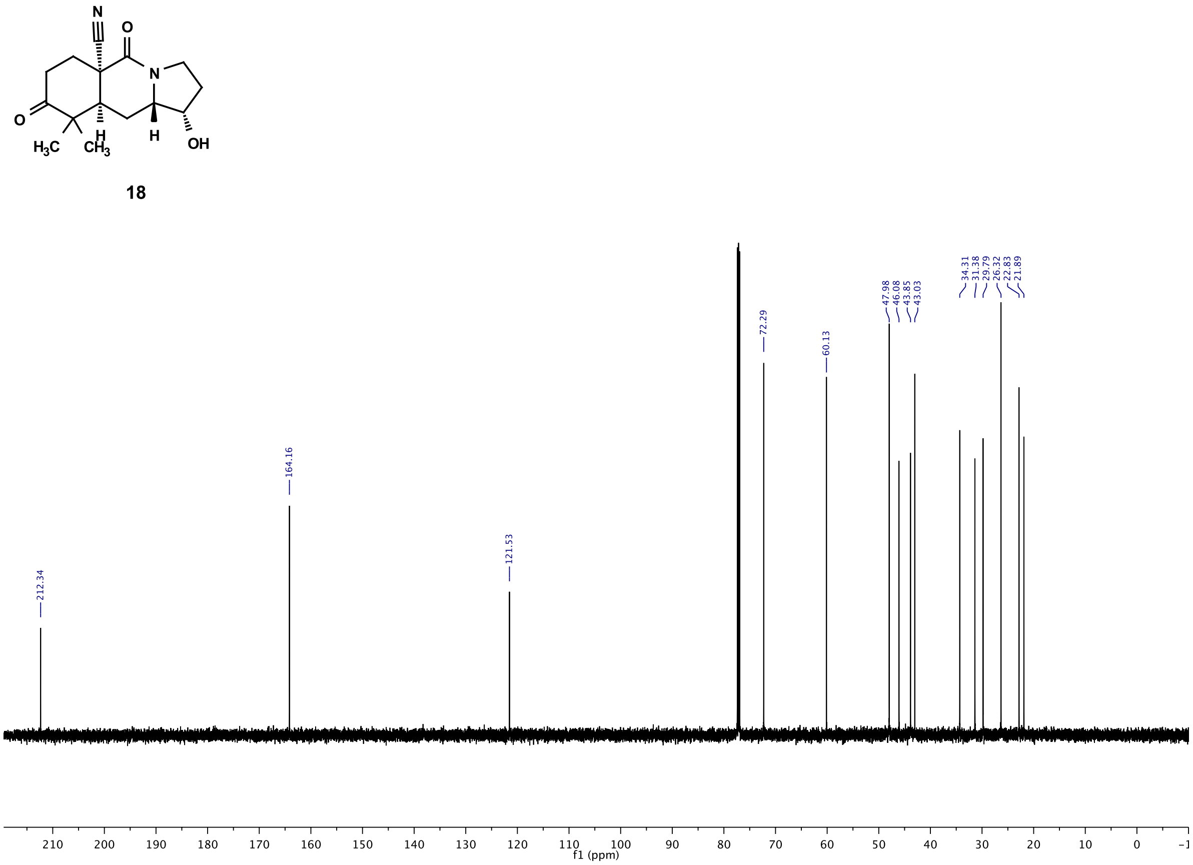

^{1}H NMR (600 MHz, $CDCl_3$)

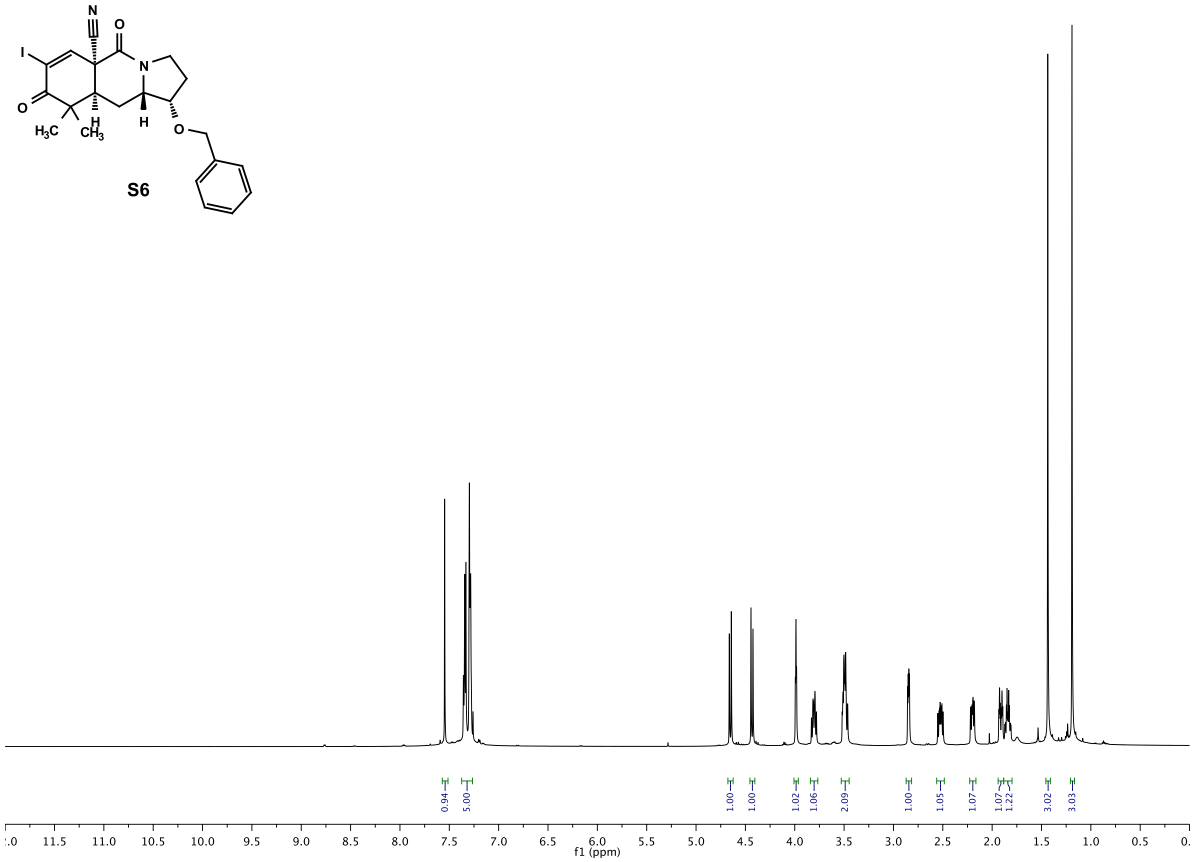

^{13}C NMR (150 MHz, $CDCl_3$)

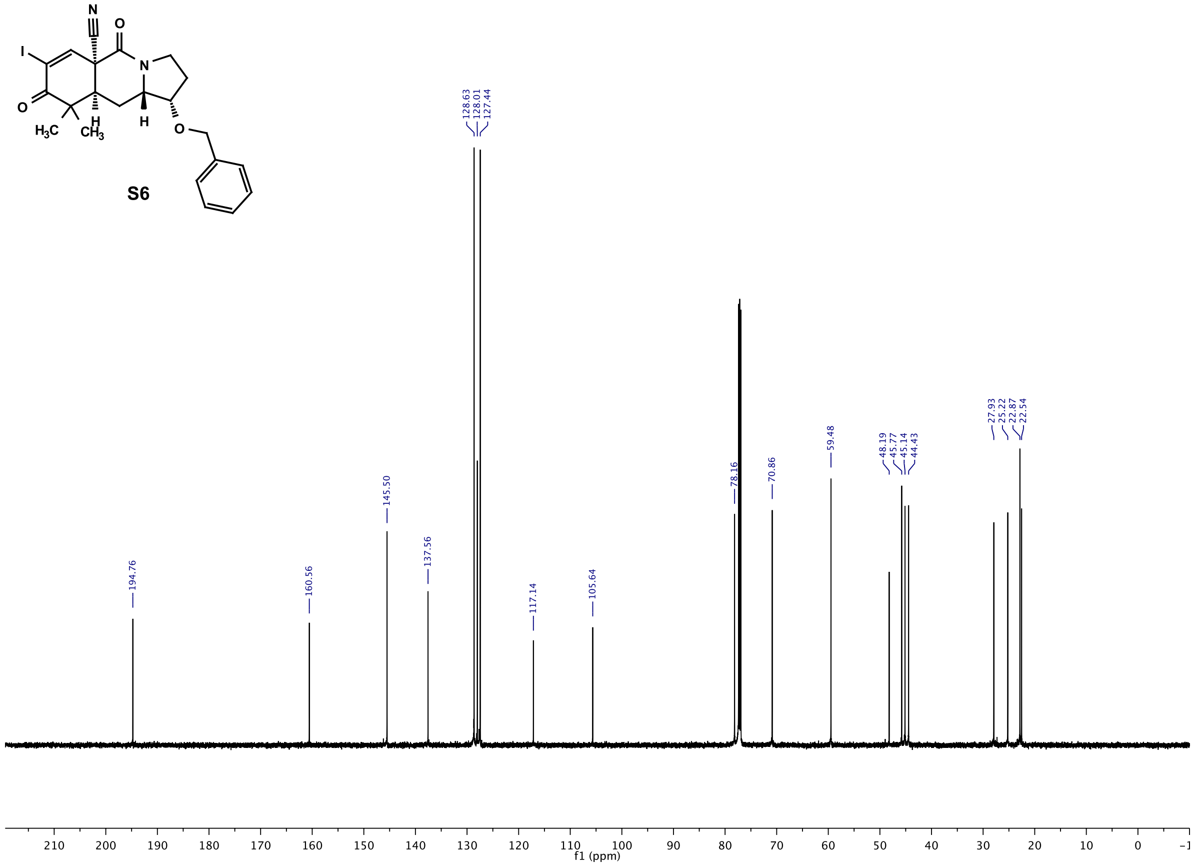

^{1}H NMR (600 MHz, $CDCl_3$)

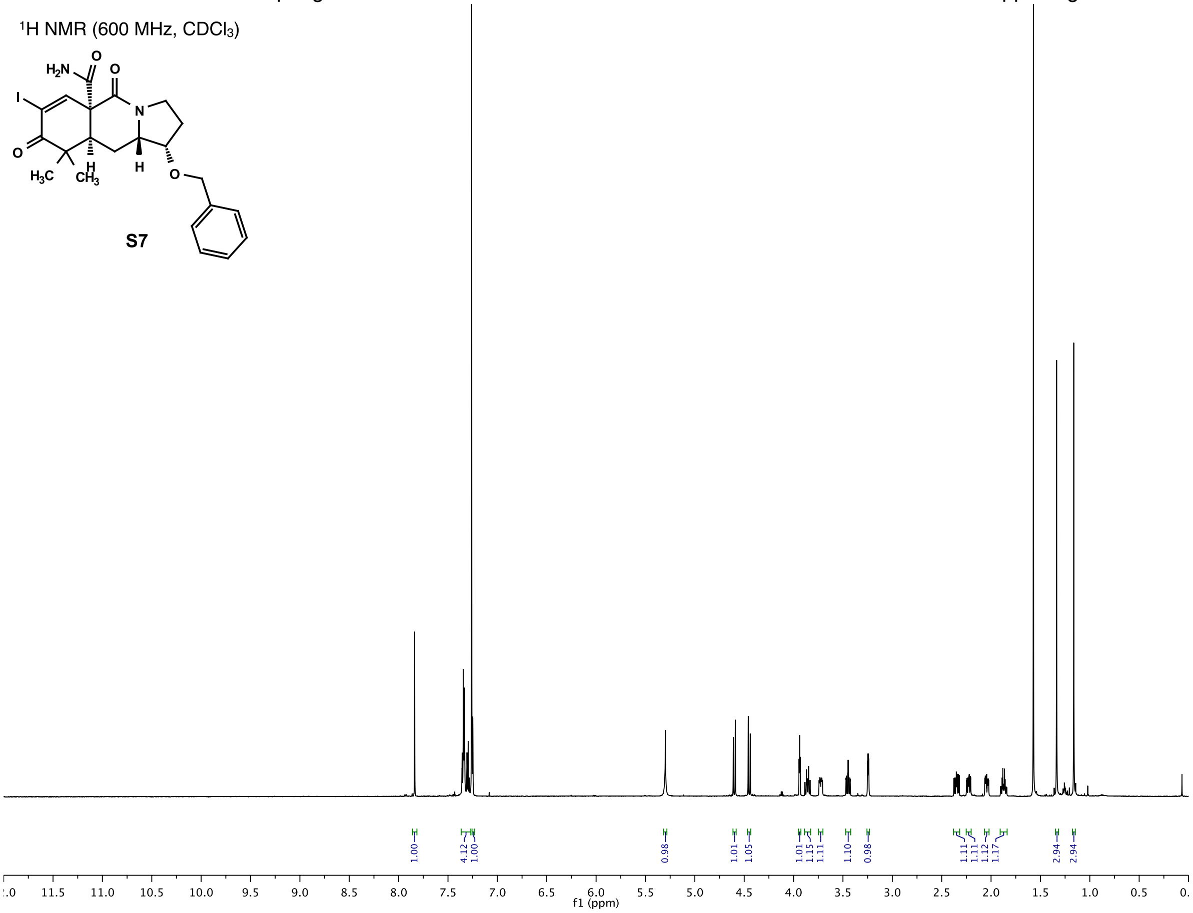

S7

^{13}C NMR (150 MHz, $CDCl_3$)

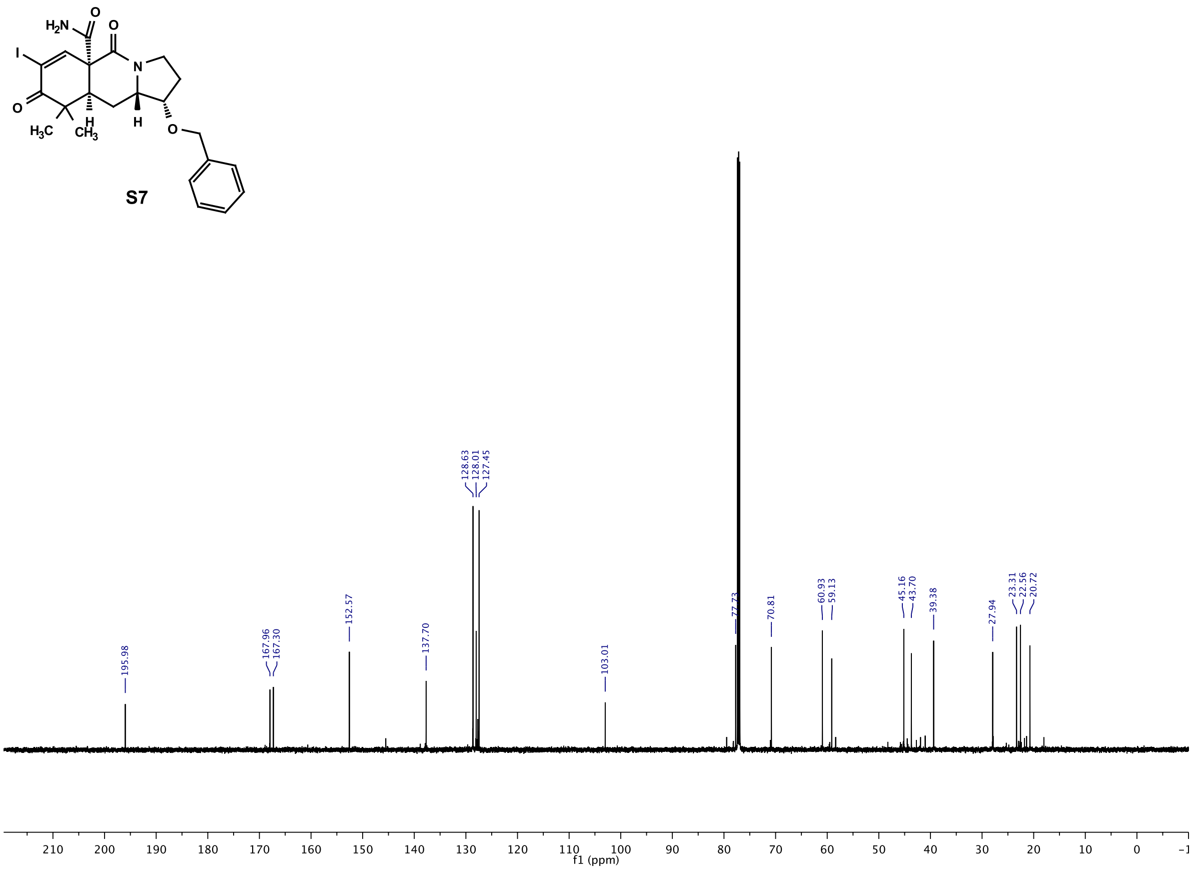

S7

^1H NMR (600 MHz, $CDCl_3$)

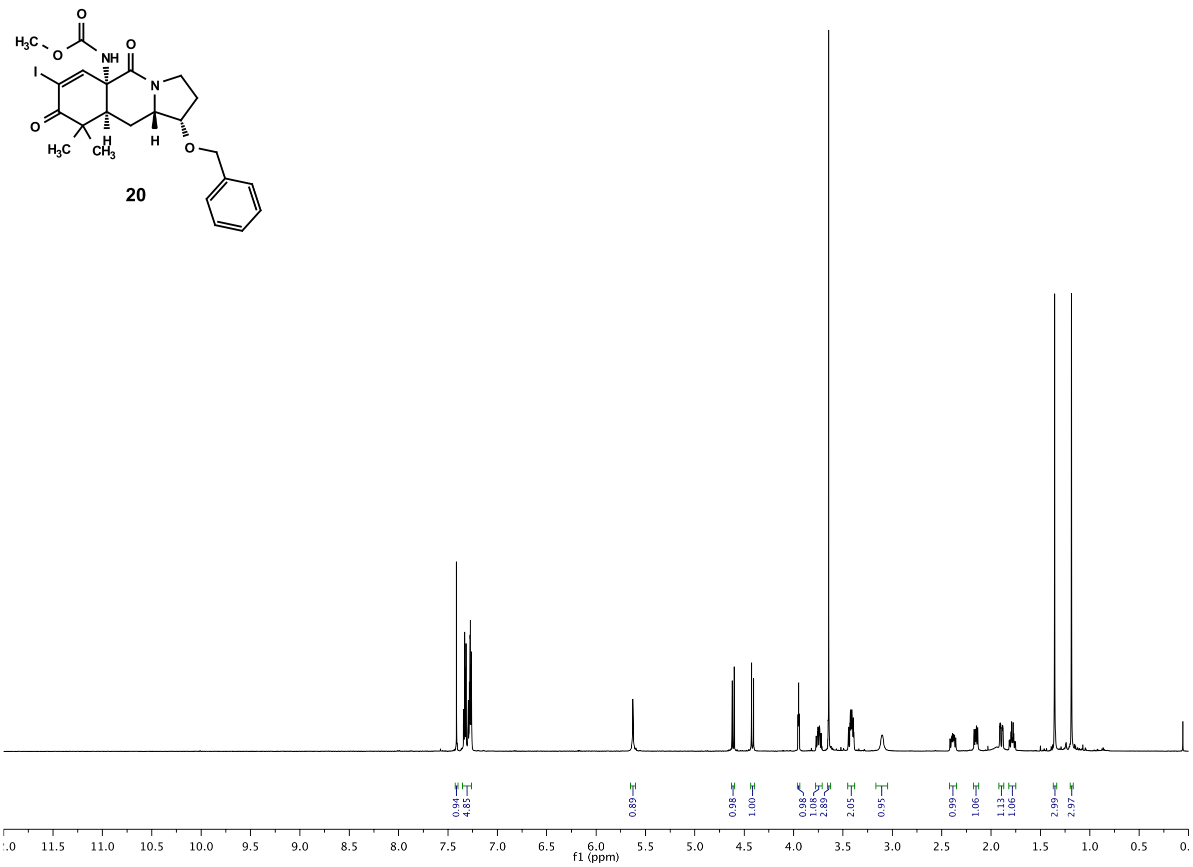

^{13}C NMR (150 MHz, $CDCl_3$)

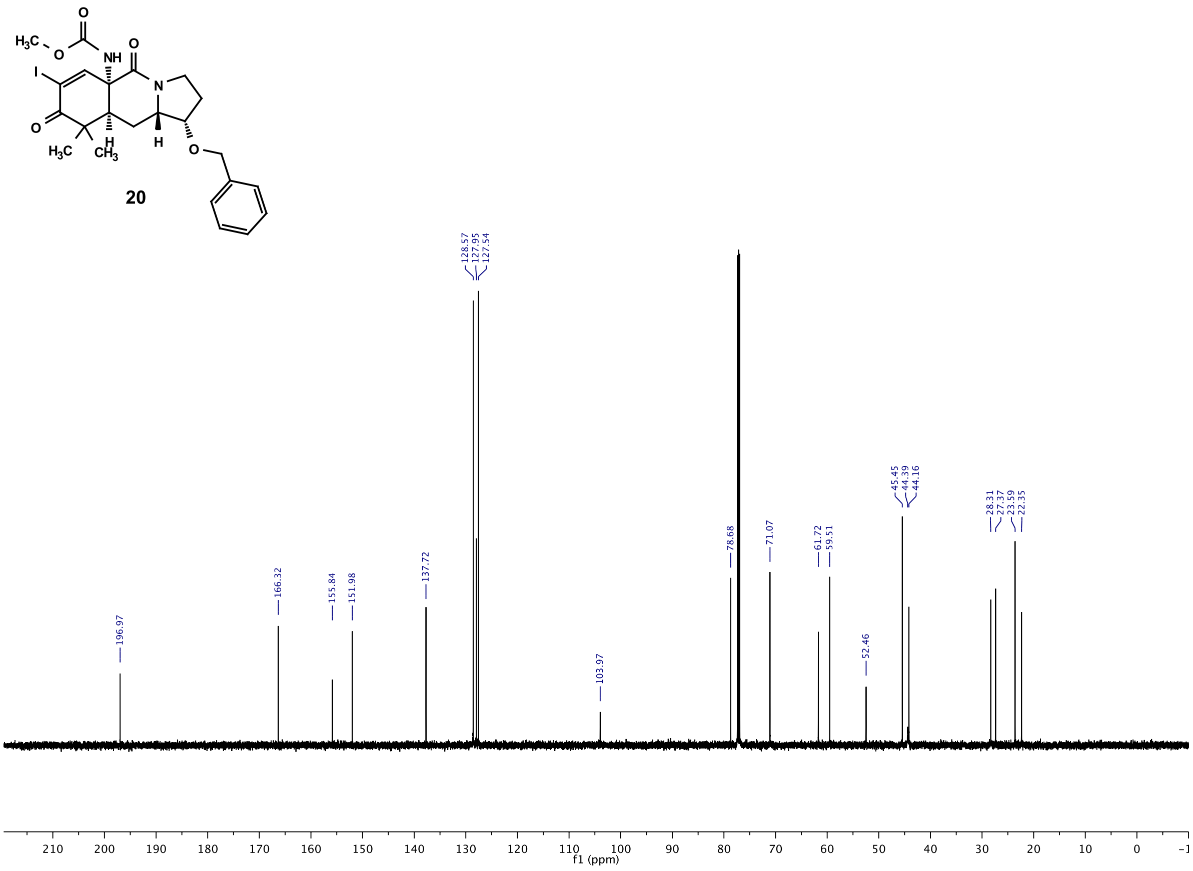

^{1}H NMR (600 MHz, $CDCl_3$)

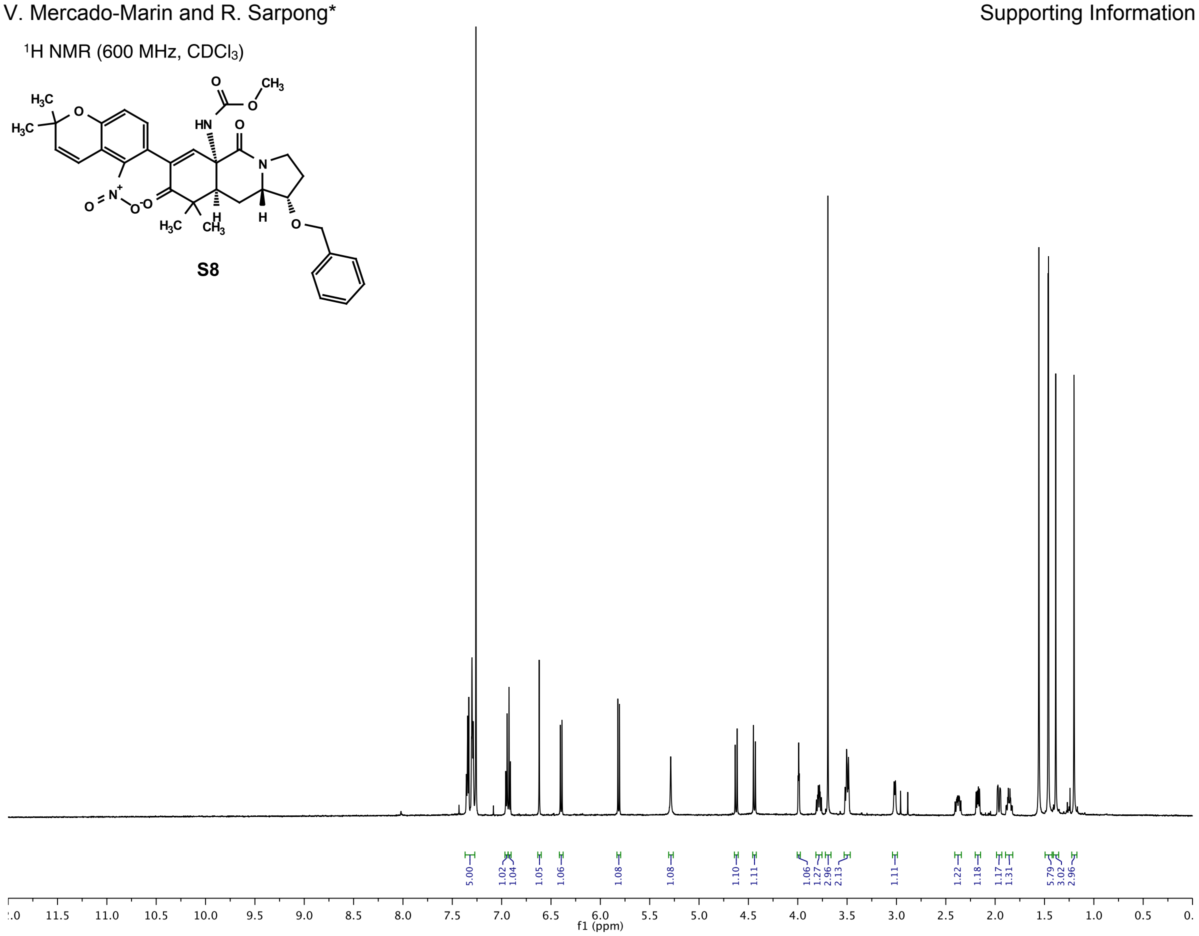

^{13}C NMR (150 MHz, $CDCl_3$)

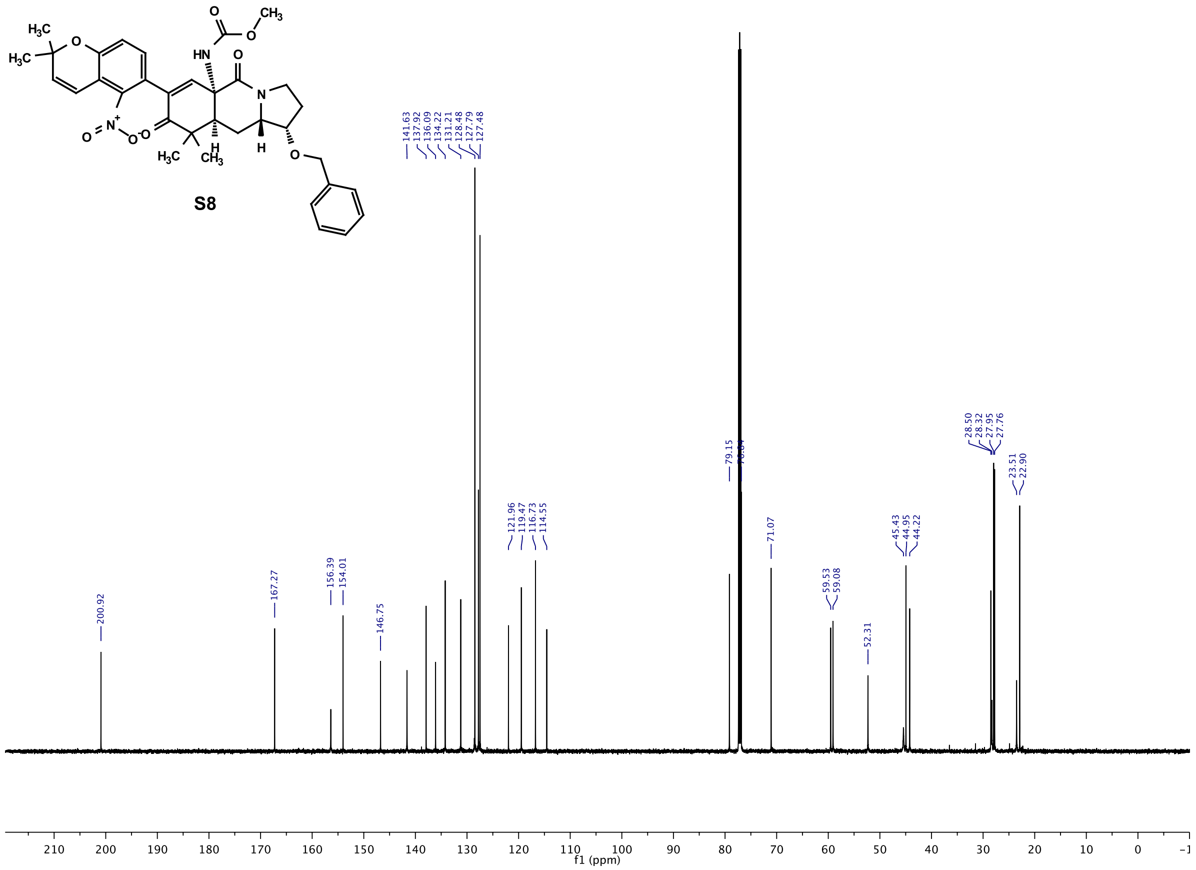

^{1}H NMR (600 MHz, $CDCl_3$)

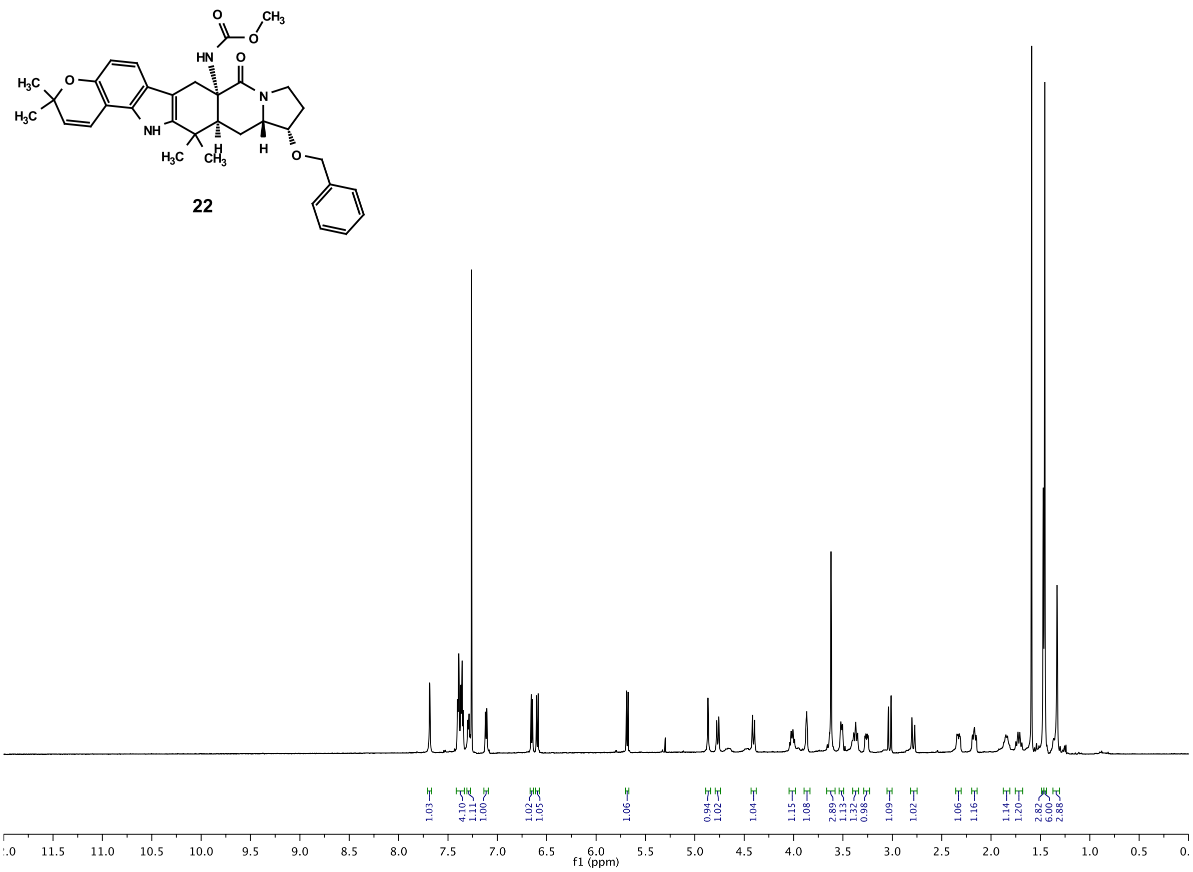

^{13}C NMR (150 MHz, $CDCl_3$)

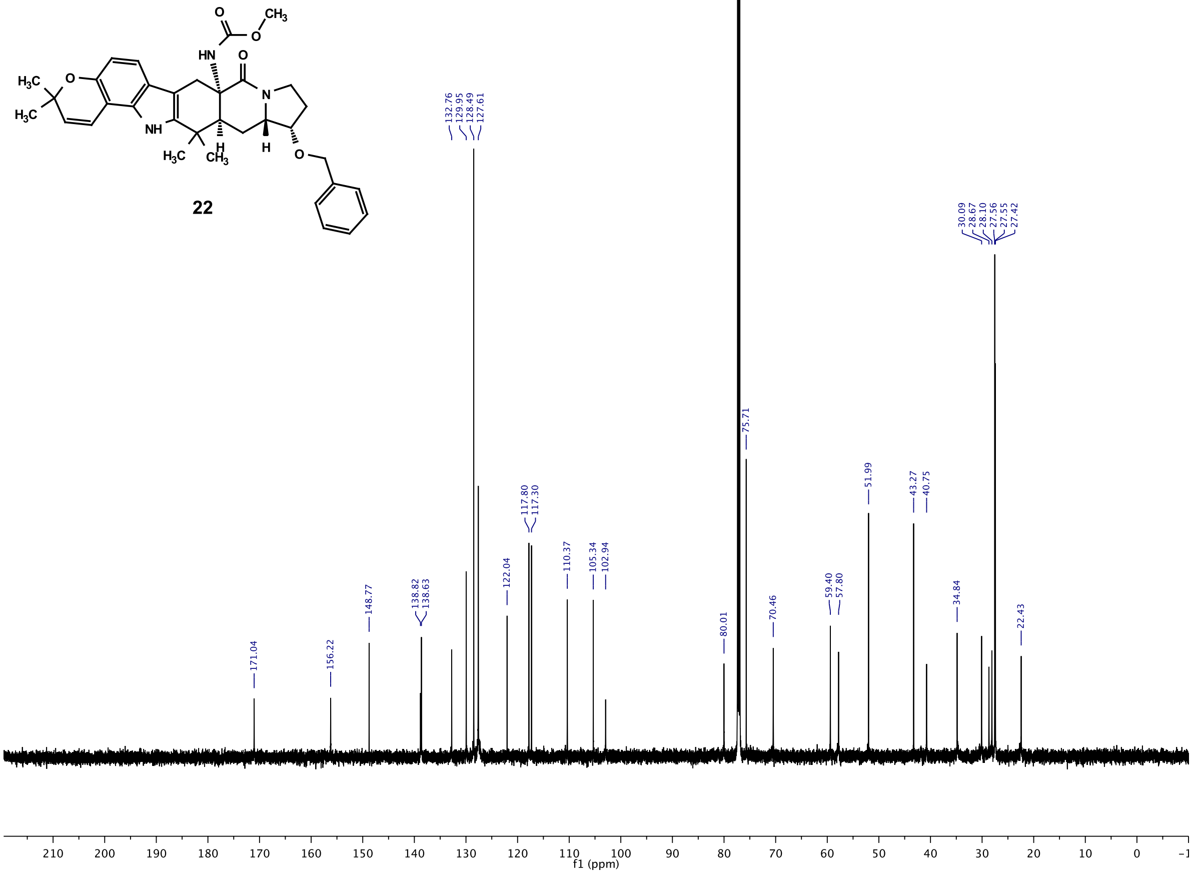

^{1}H NMR (600 MHz, $CDCl_3$)

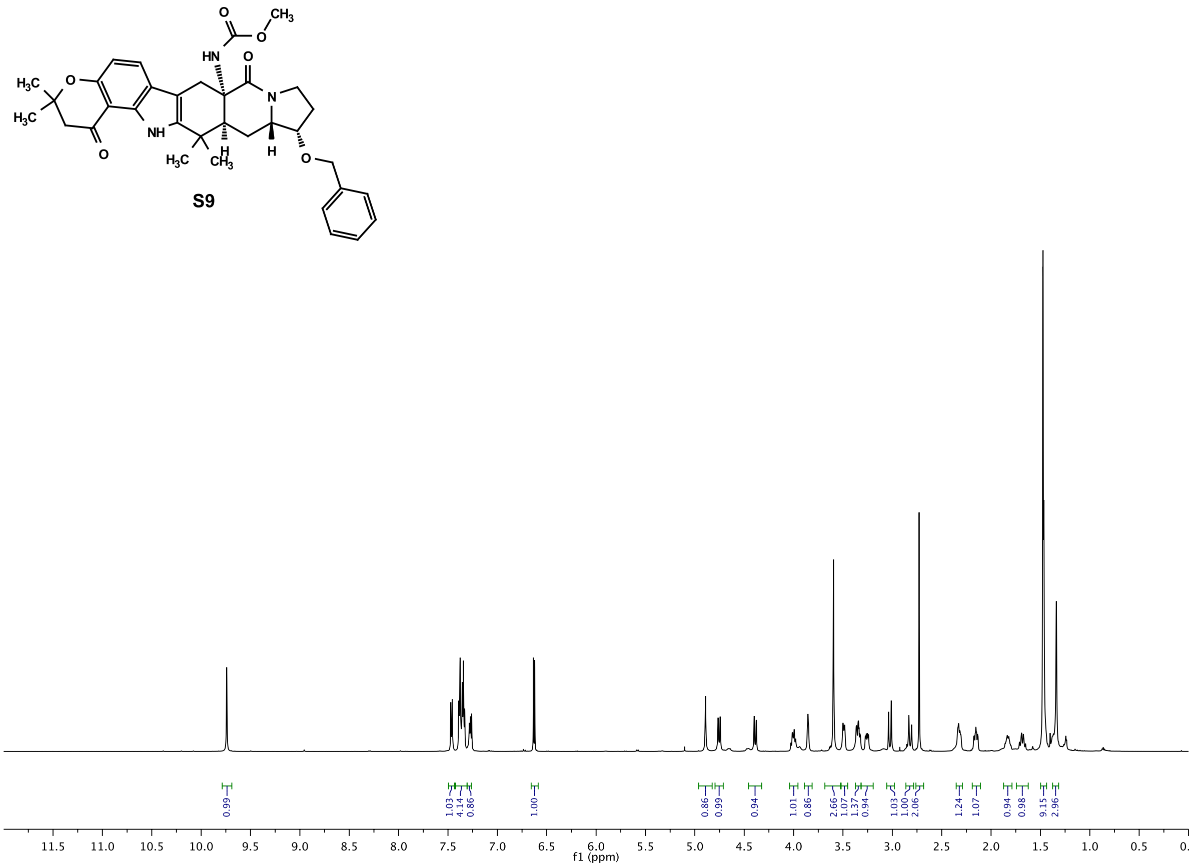

^{13}C NMR (150 MHz, $CDCl_3$)

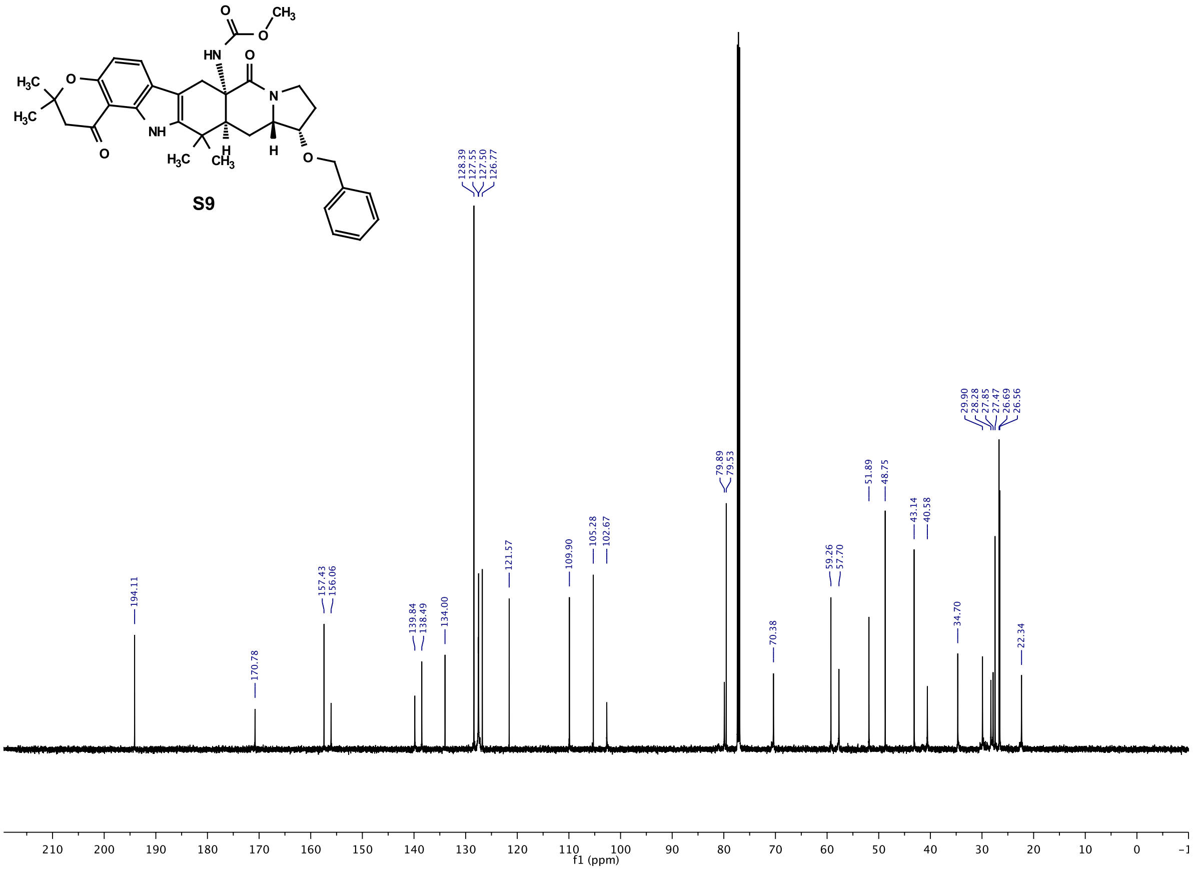

^{1}H NMR (600 MHz, $CDCl_3$)

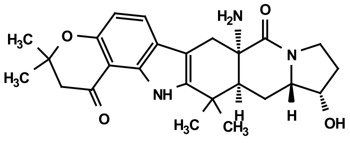

23

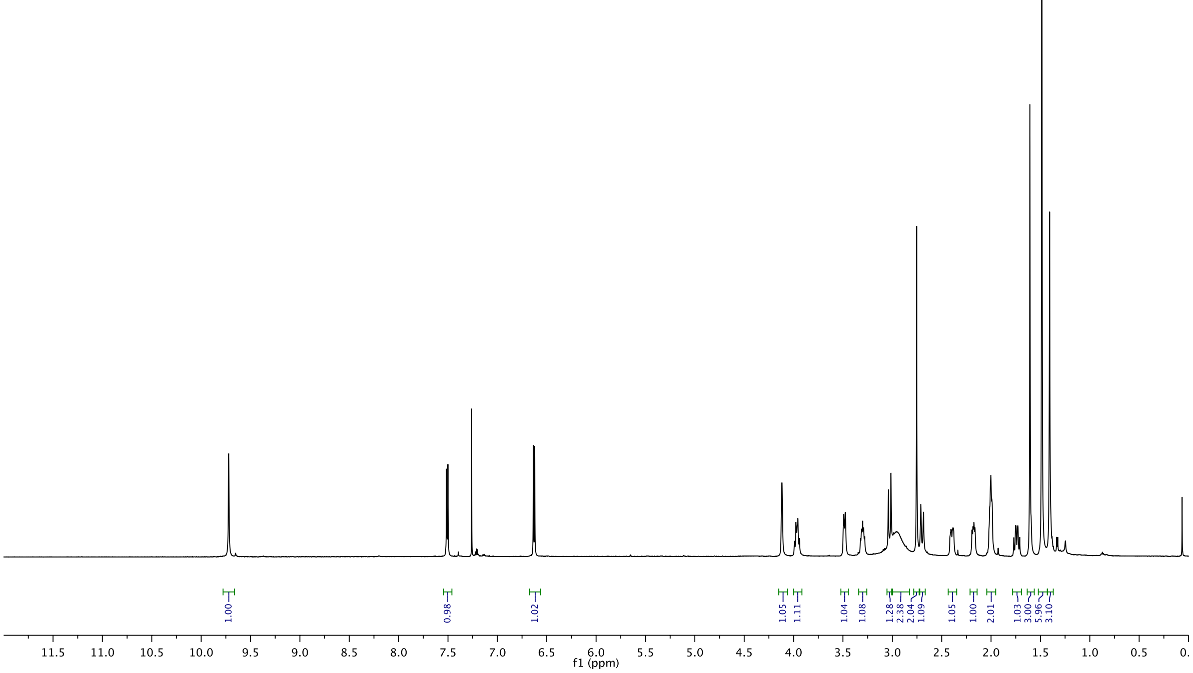

^{13}C NMR (150 MHz, $CDCl_3$)

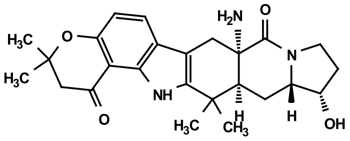

23

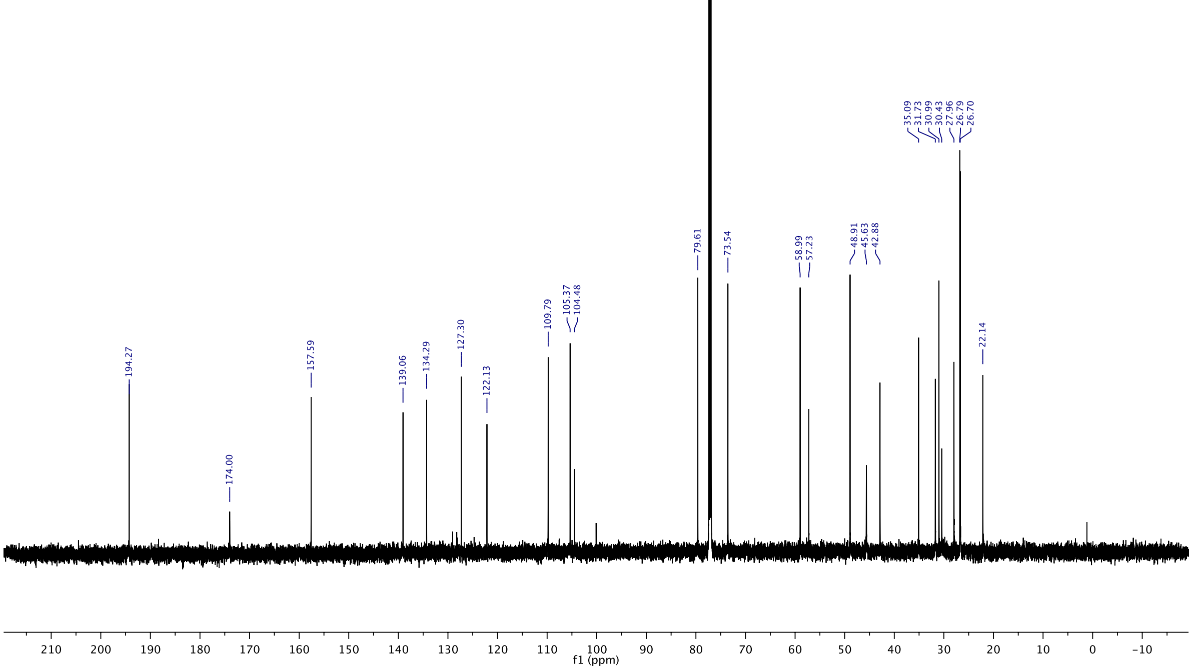

^{1}H NMR (600 MHz, $(CD_3)_2SO$)

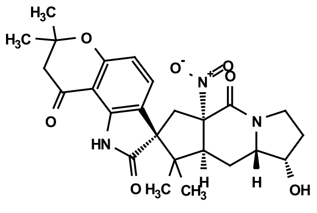

24

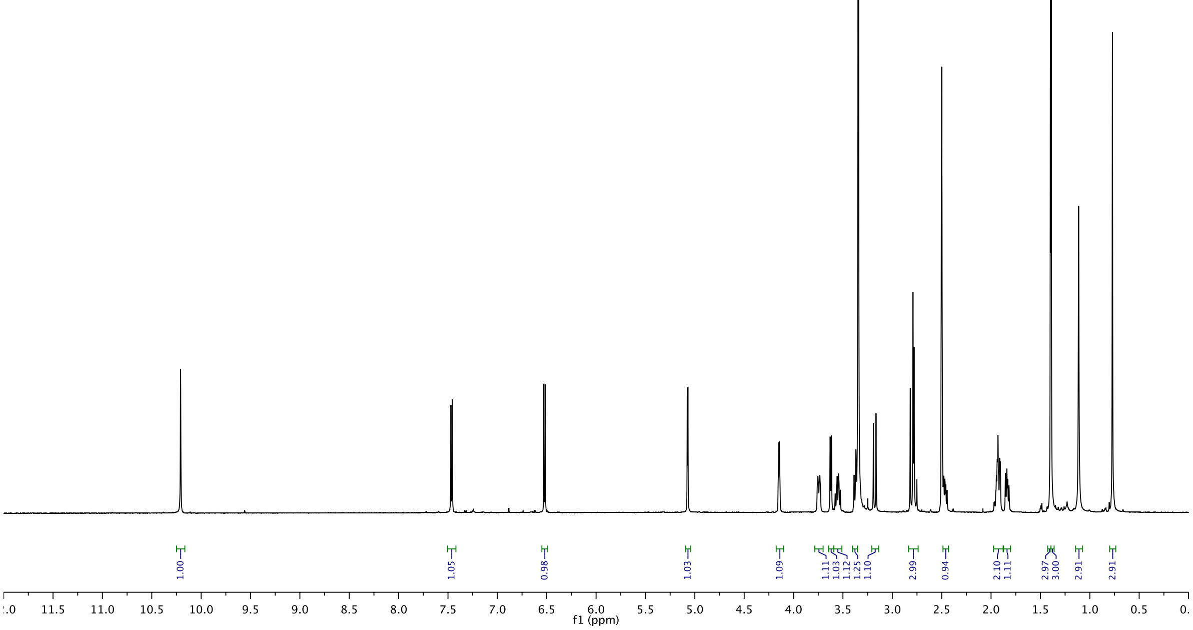

^{13}C NMR (150 MHz, $(CD_3)_2SO$)

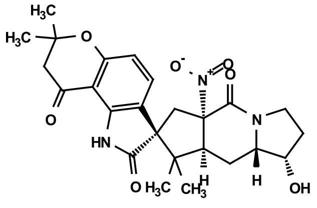

24

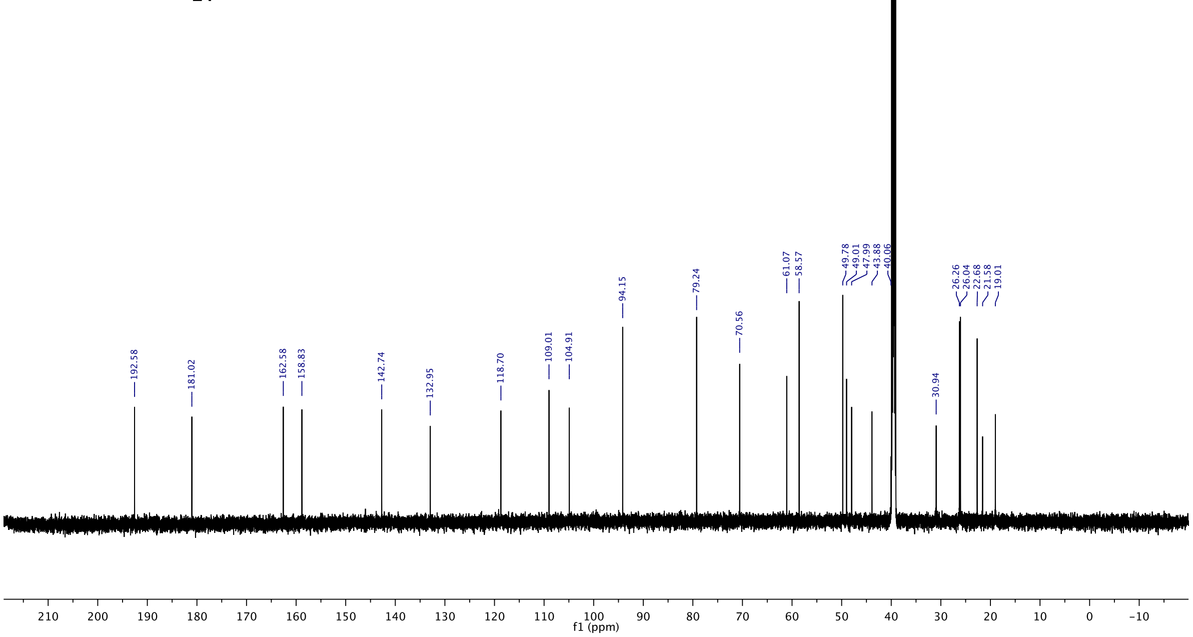

^{1}H NMR (600 MHz, CD_3OD)

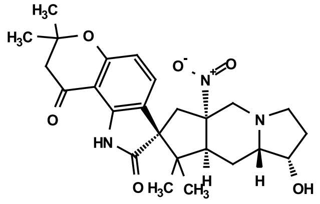

(–)-17-hydroxy-citrinalin B **(7)**

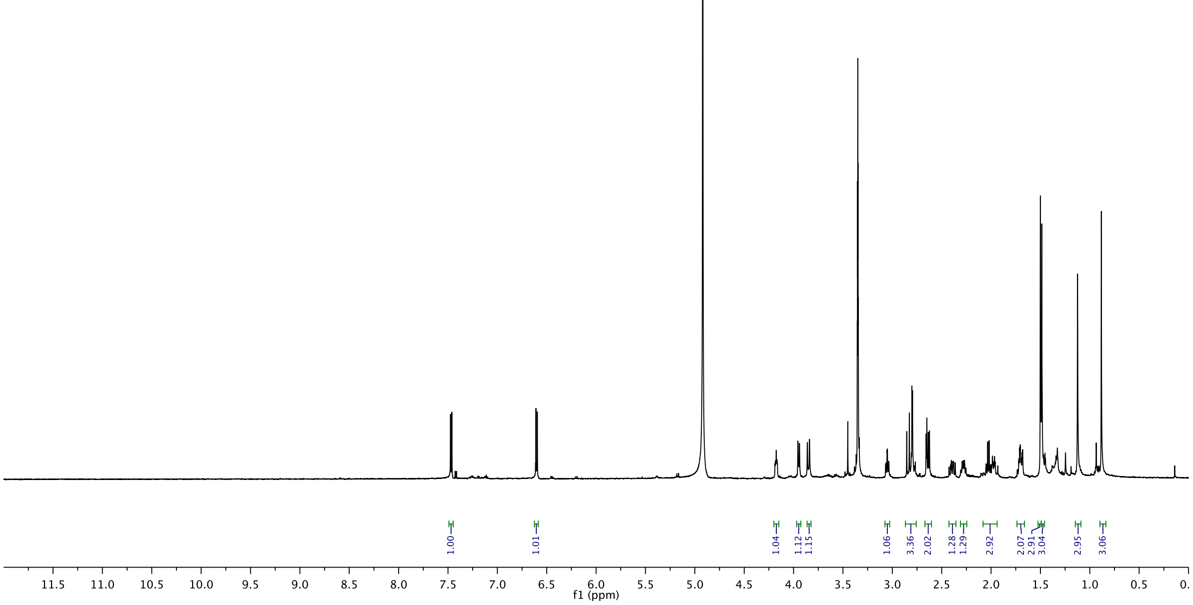

^{13}C NMR (150 MHz, CD_3OD)

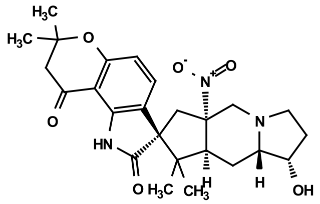

(–)-17-hydroxy-citrinalin B **(7)**

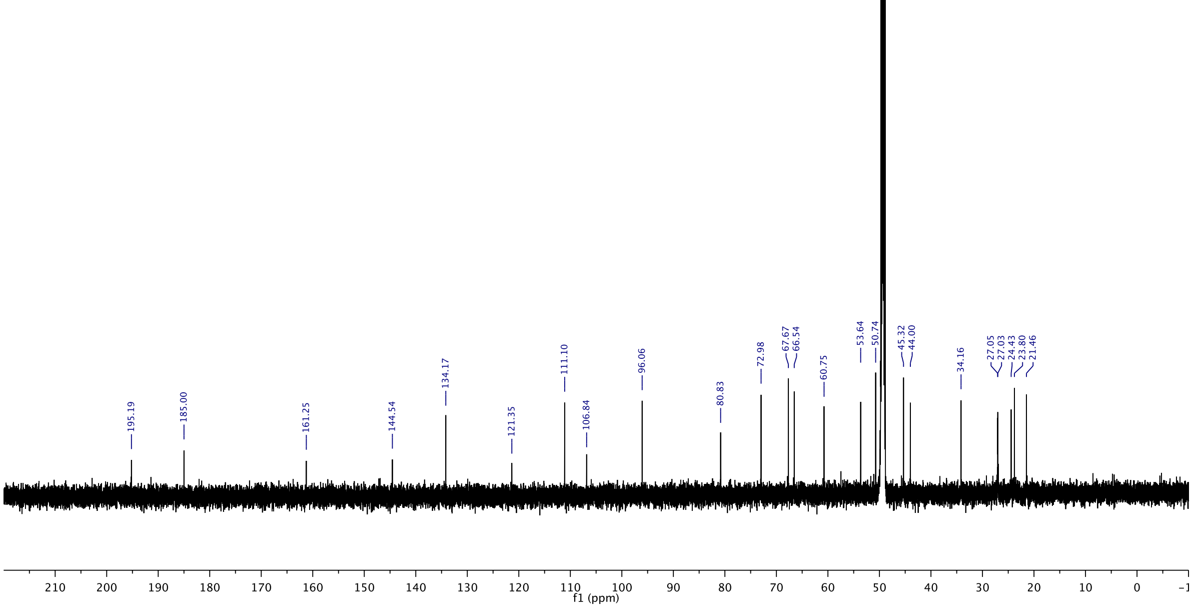

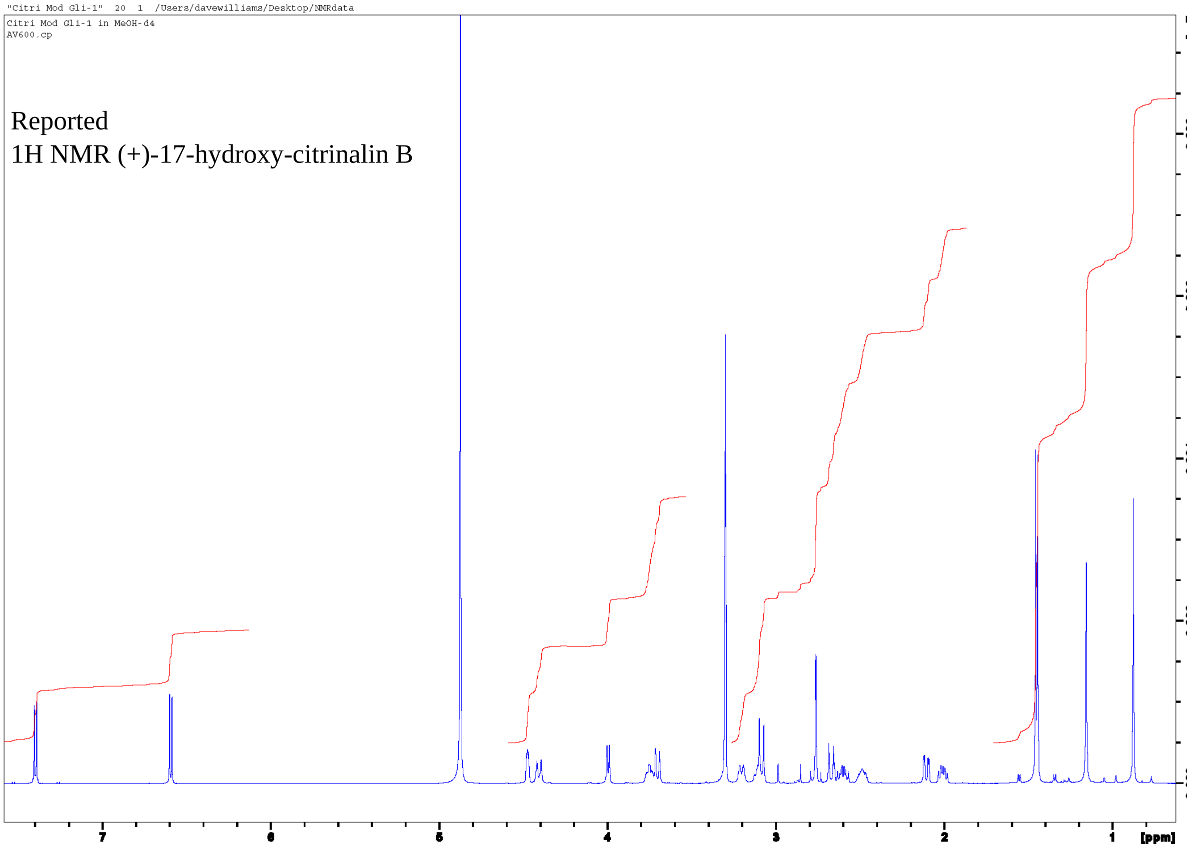

^{1}H NMR (600 MHz, CD_3OD)

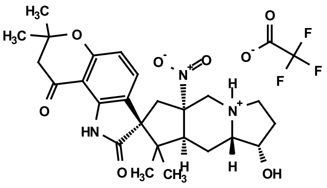

(–)-17-hydroxy-citrinalin B-TFA (**7a**)

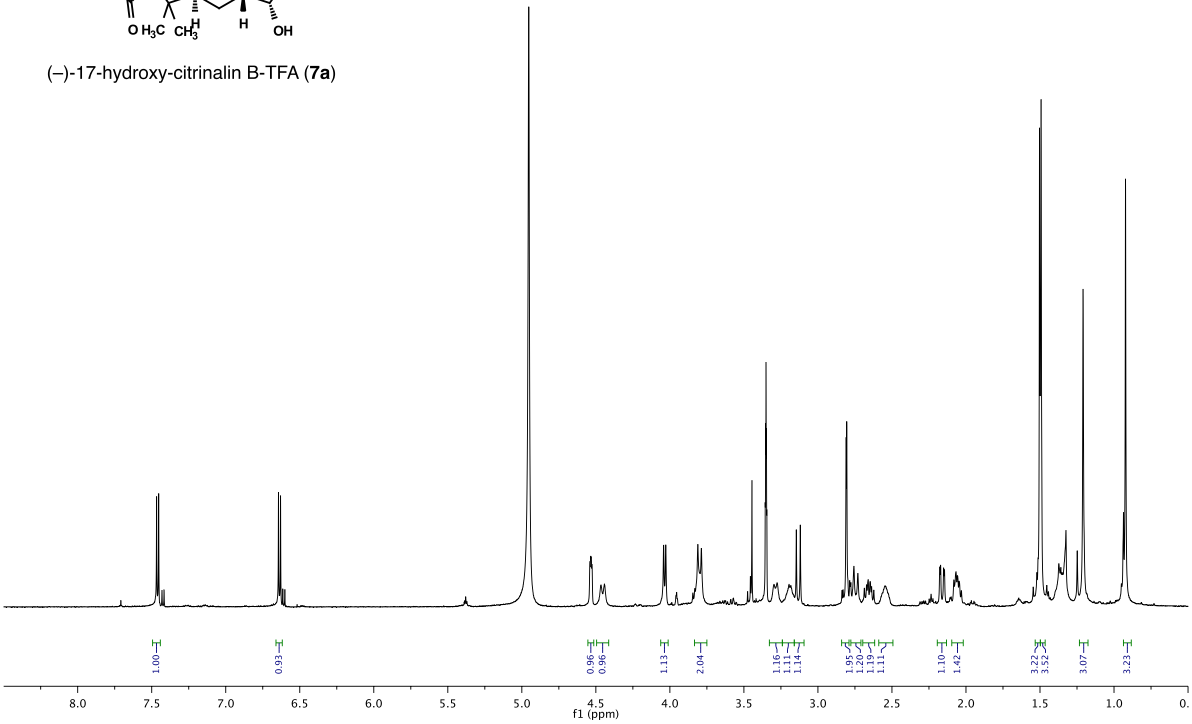

Reported
13C Natural (+)-17-hydroxy-citrinalin B

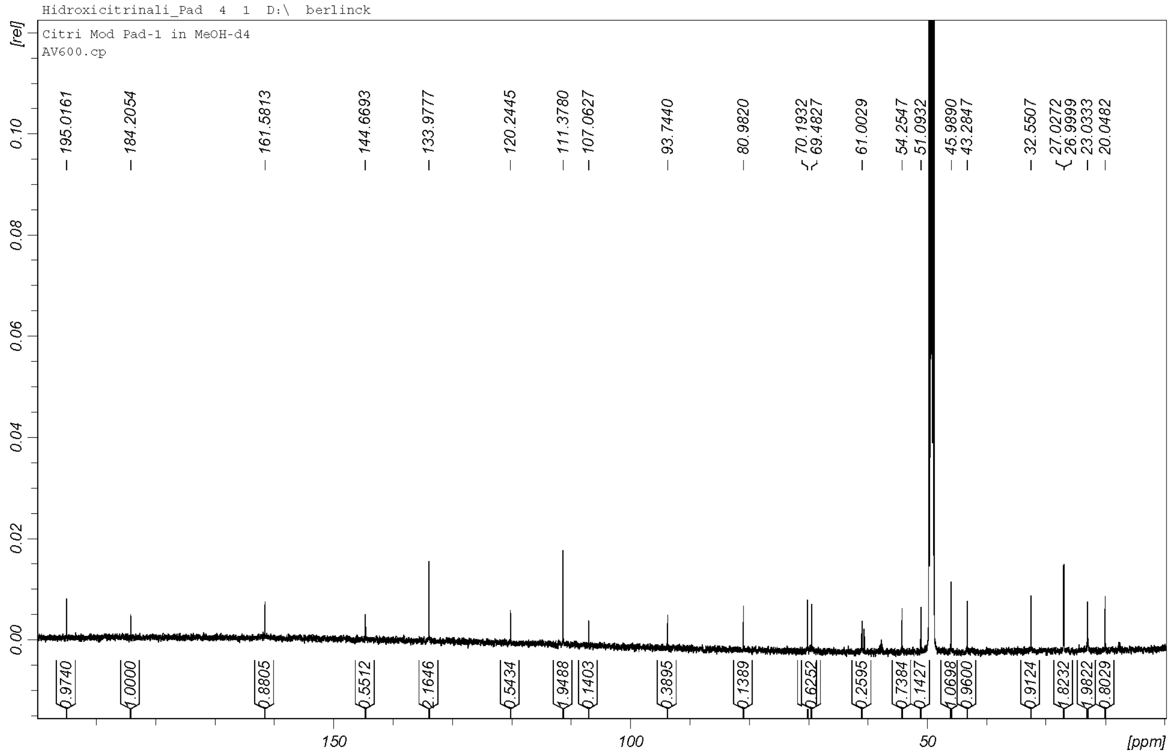

^{13}C NMR (150 MHz, CD_3OD)

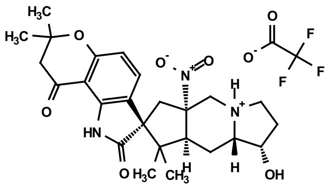

(–)-17-hydroxy-citrinalin B-TFA (**7a**)

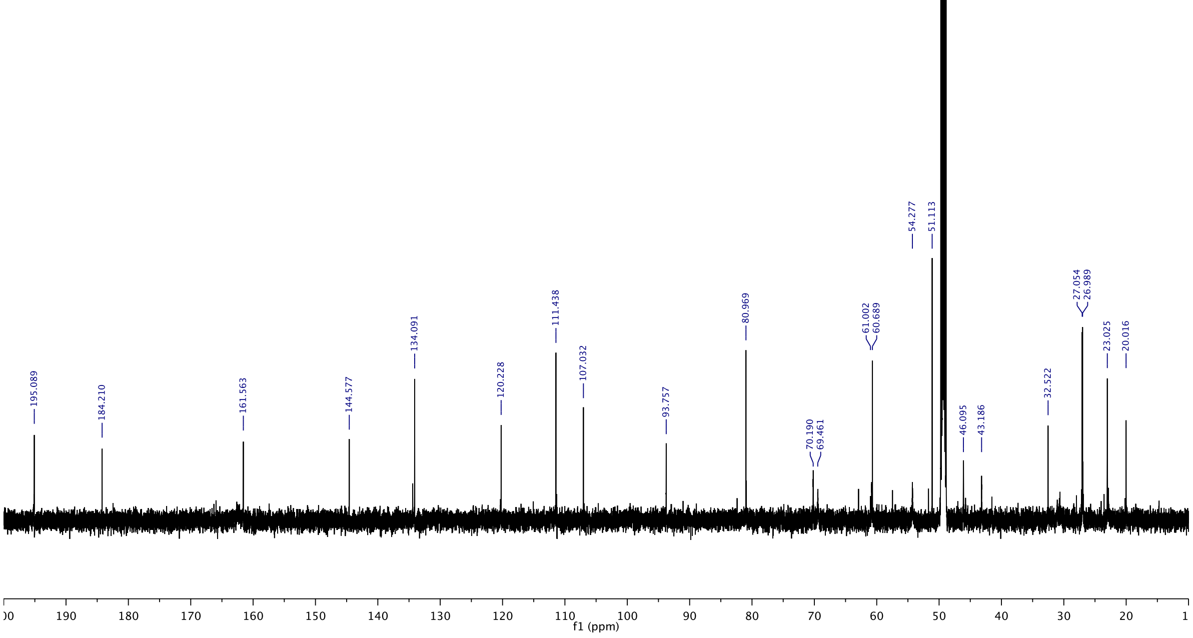

^{1}H NMR (600 MHz, CD_3OD)

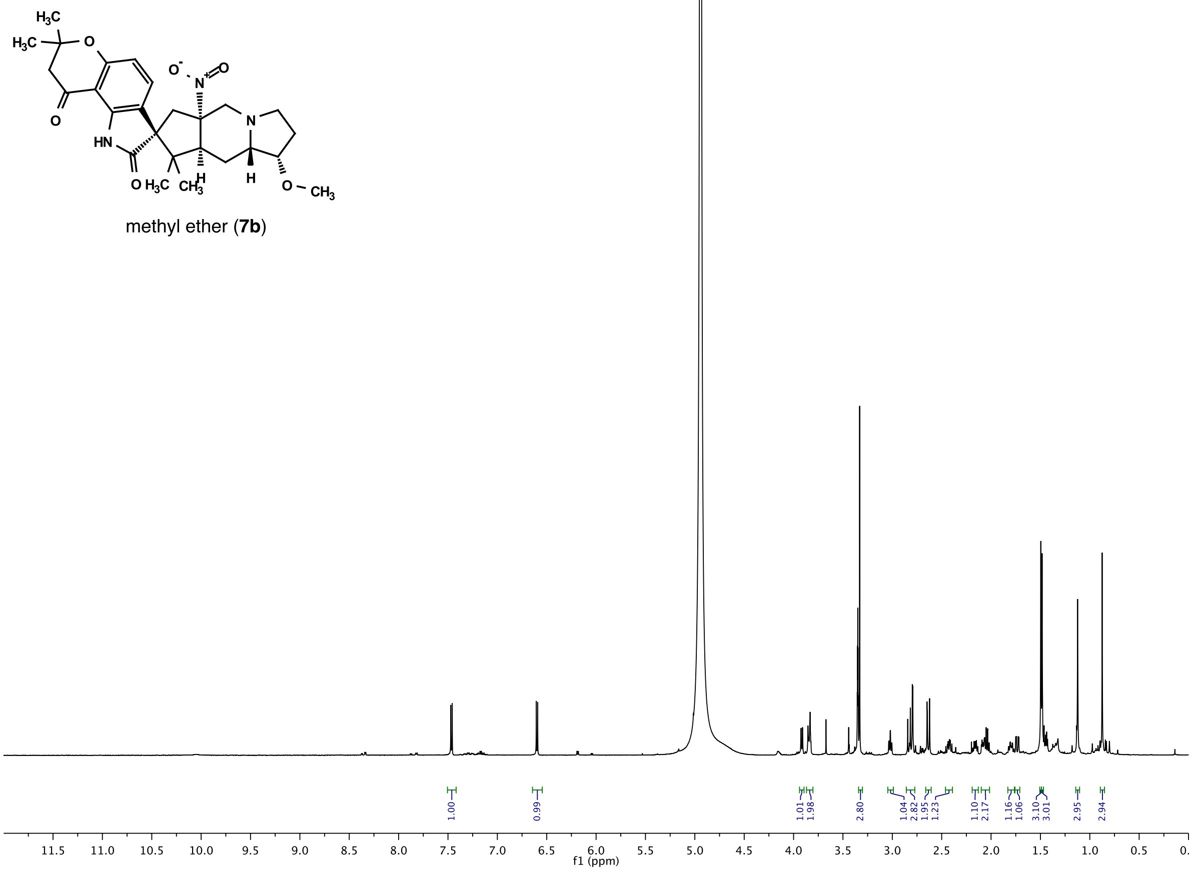

^{13}C NMR (150 MHz, CD_3OD)

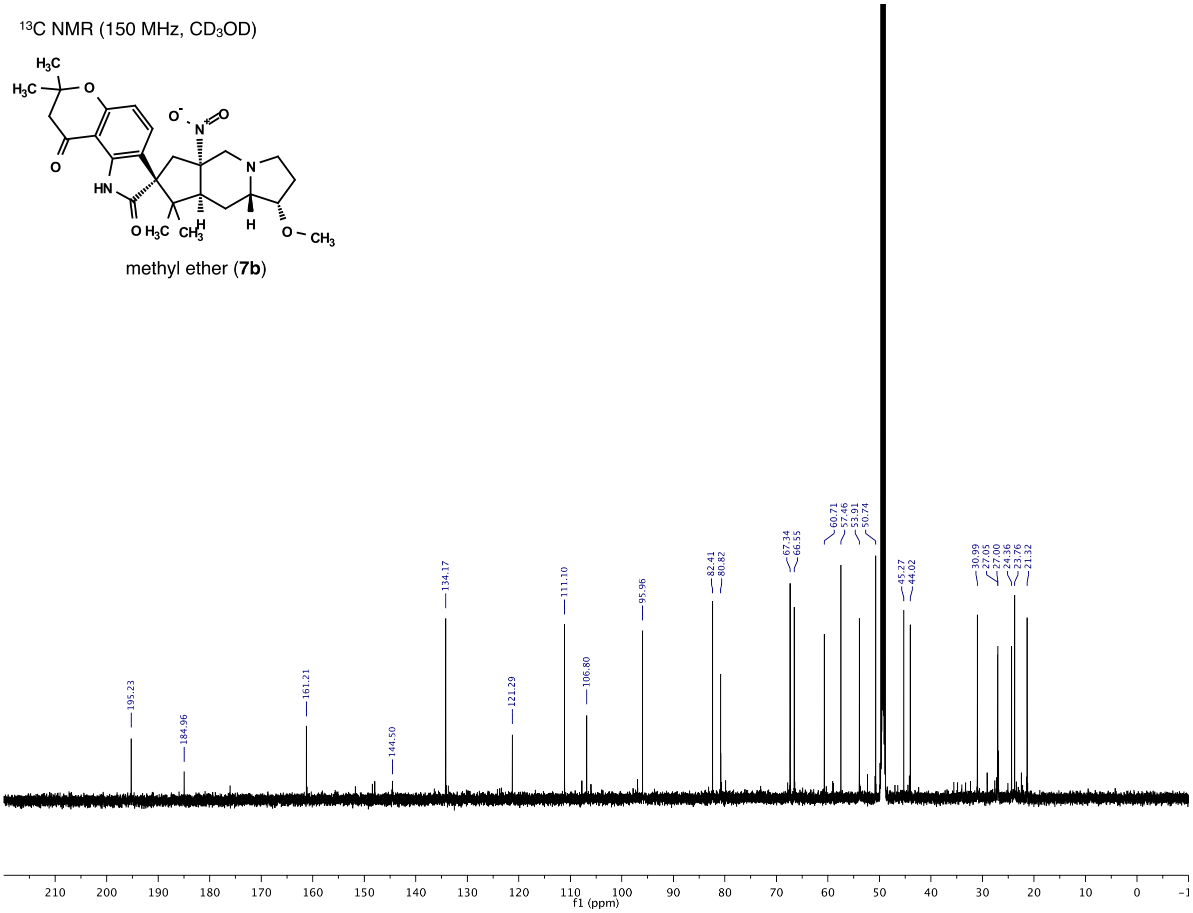

^{1}H NMR (600 MHz, $CDCl_3$)

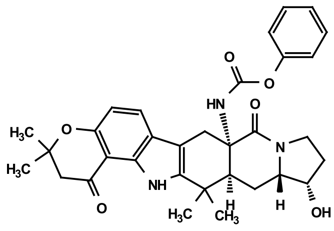

25

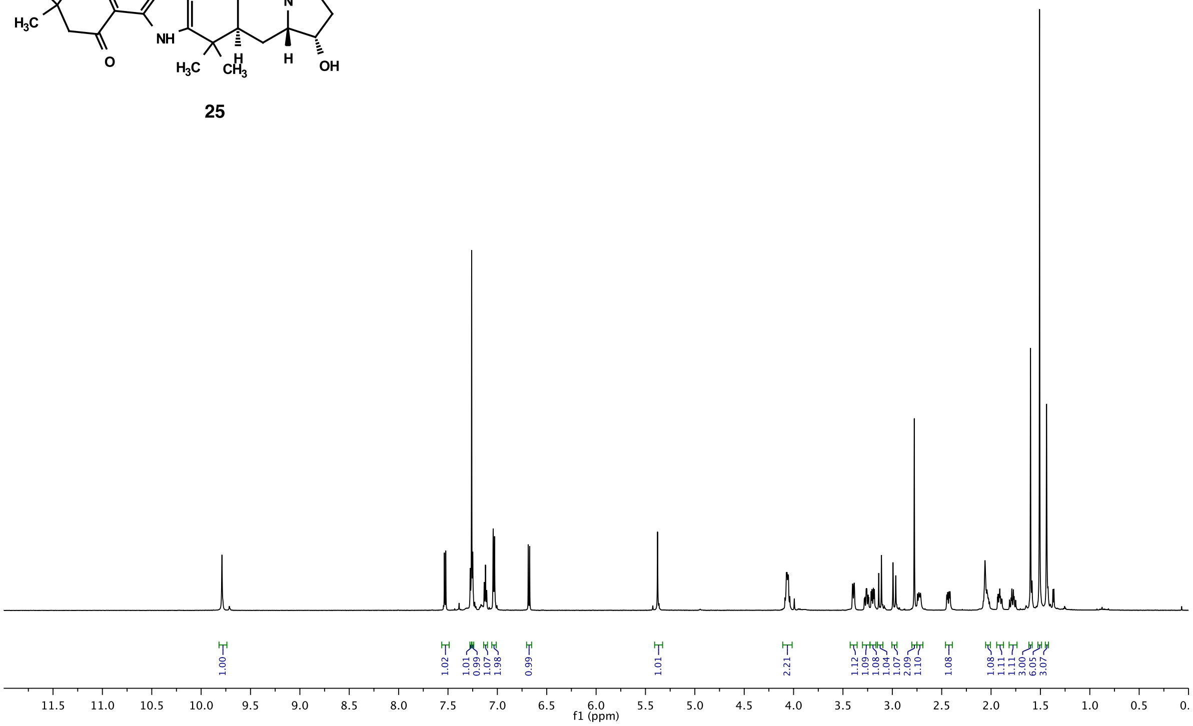

^{13}C NMR (150 MHz, $CDCl_3$)

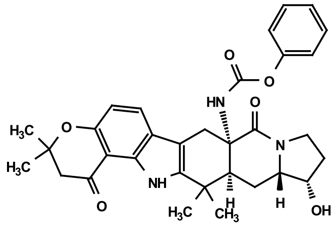

25

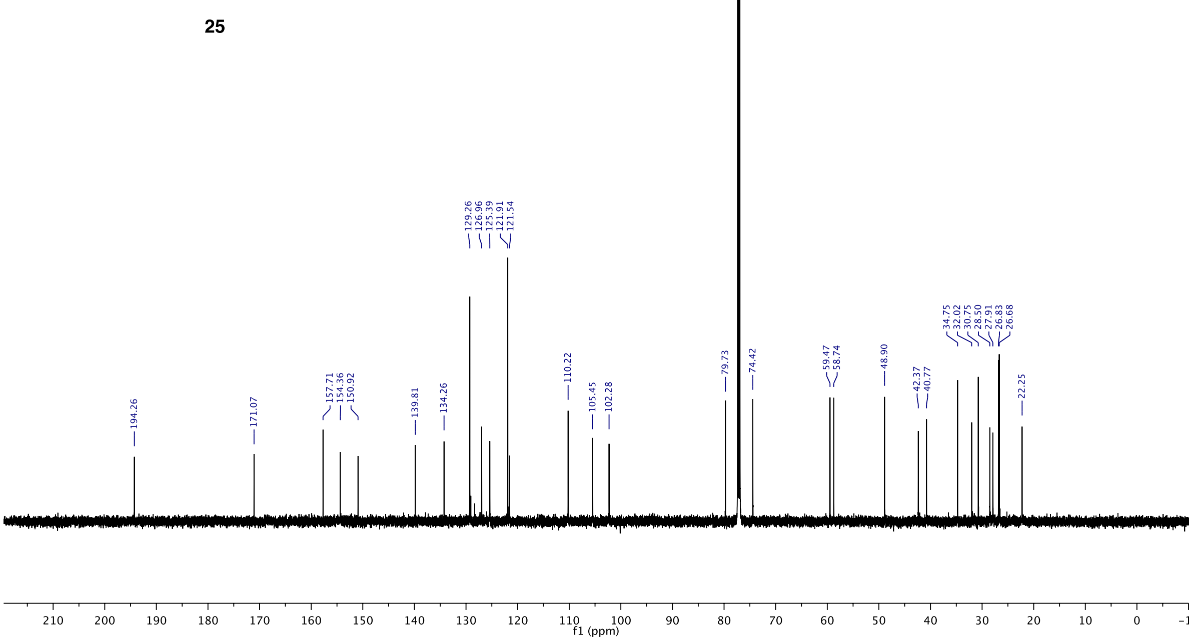

^{1}H NMR (600 MHz, $CDCl_3$)

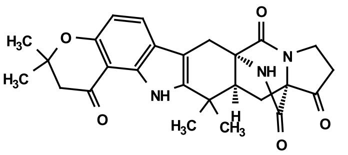

27

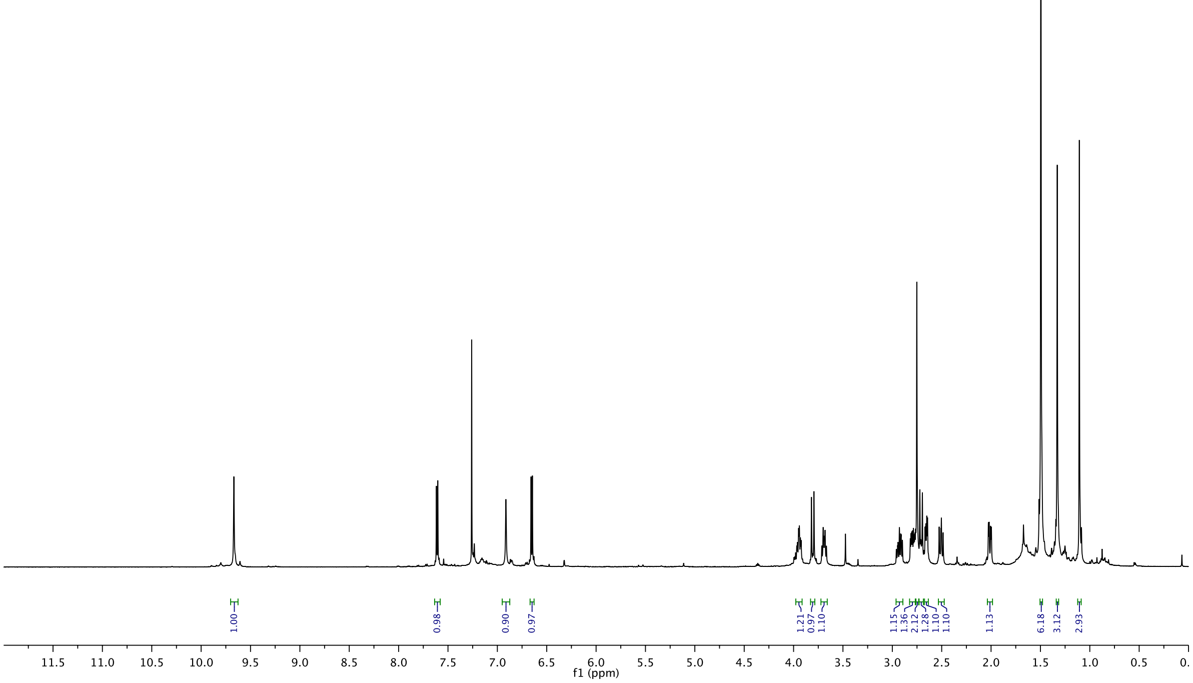

^{13}C NMR (150 MHz, $CDCl_3$)

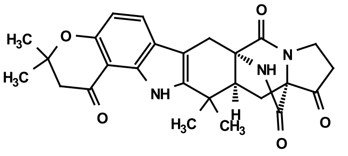

27

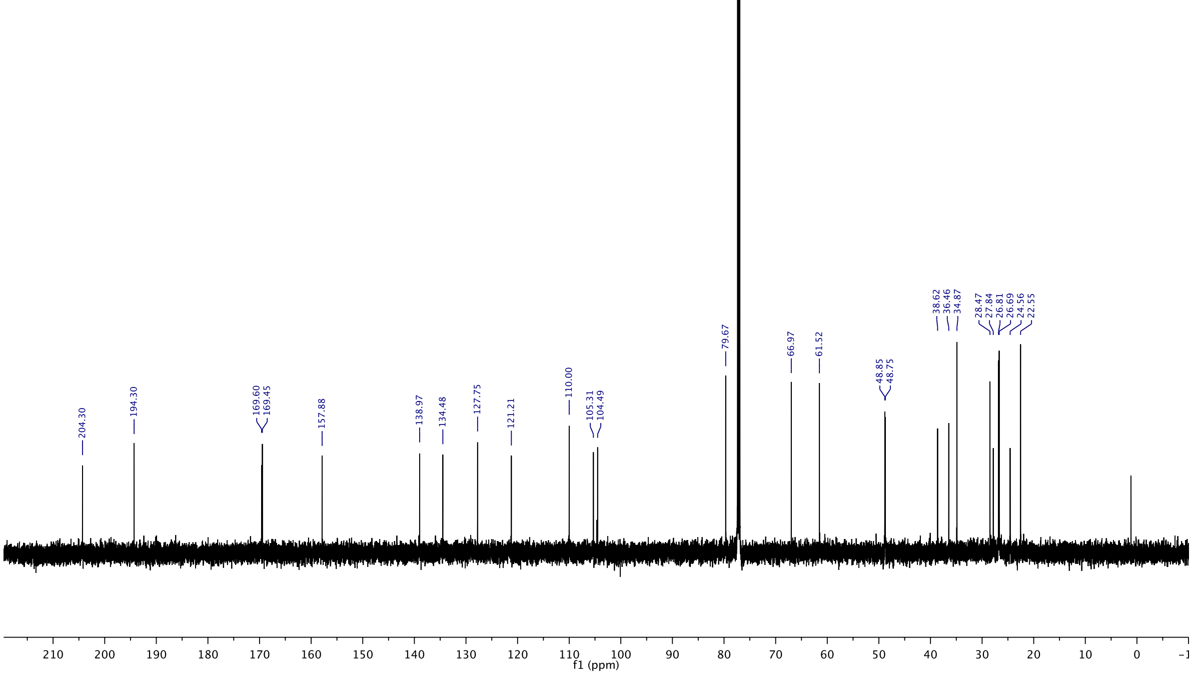

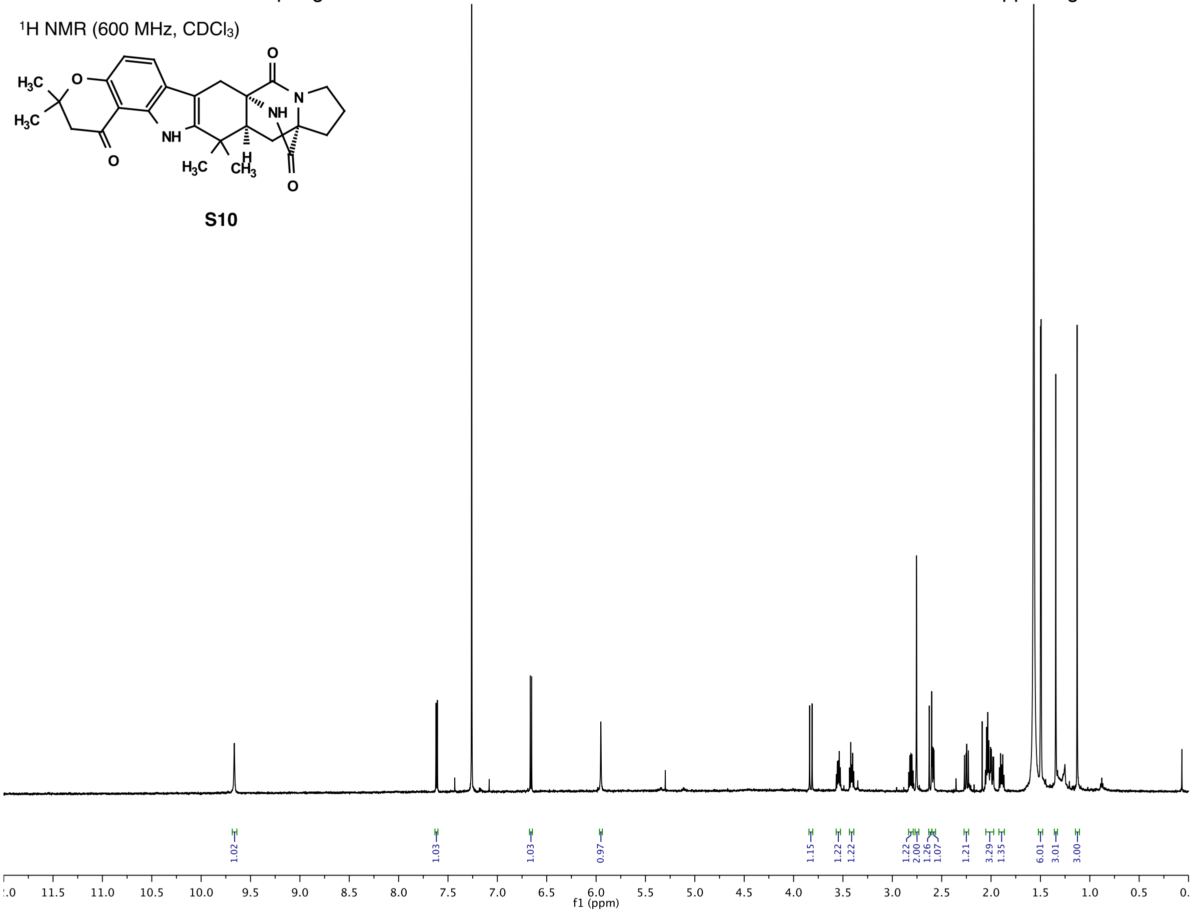

^{1}H NMR (600 MHz, CDCl$_3$)
S10
1.02
1.03
1.03
0.97
1.15
1.22
1.22
1.22
2.00
1.26
1.07
1.21
3.29
1.35
6.01
3.01
3.00
f1 (ppm)

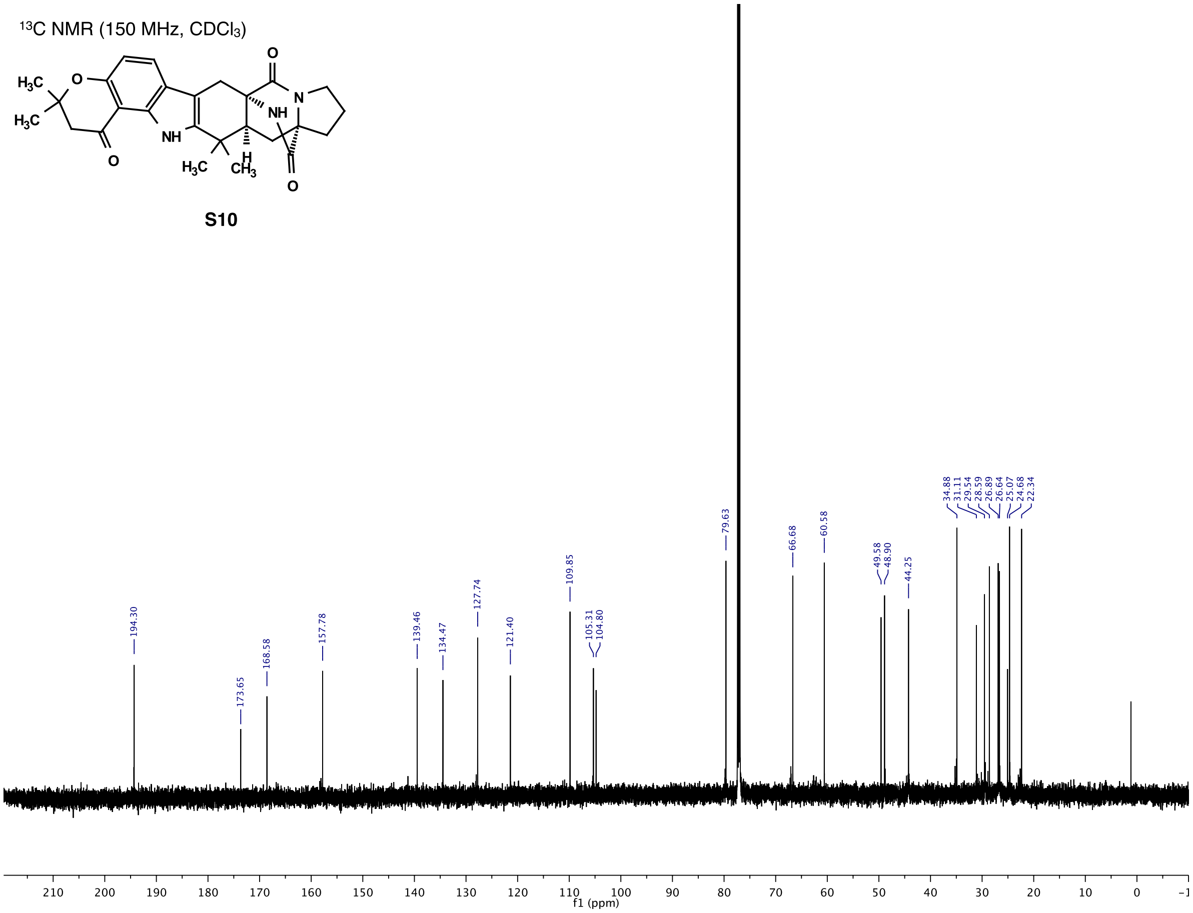

^{13}C NMR (150 MHz, CDCl$_3$)
S10
194.30
173.65
168.58
157.78
139.46
134.47
127.74
121.40
109.85
105.31
104.80
79.63
66.68
60.58
49.58
48.90
44.25
34.88
31.11
29.54
28.59
26.89
26.64
25.07
24.68
22.34
f1 (ppm)

Reported

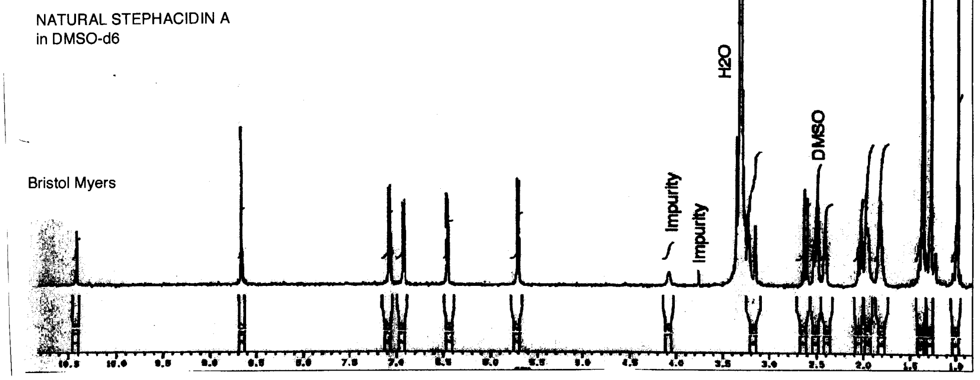

Reported

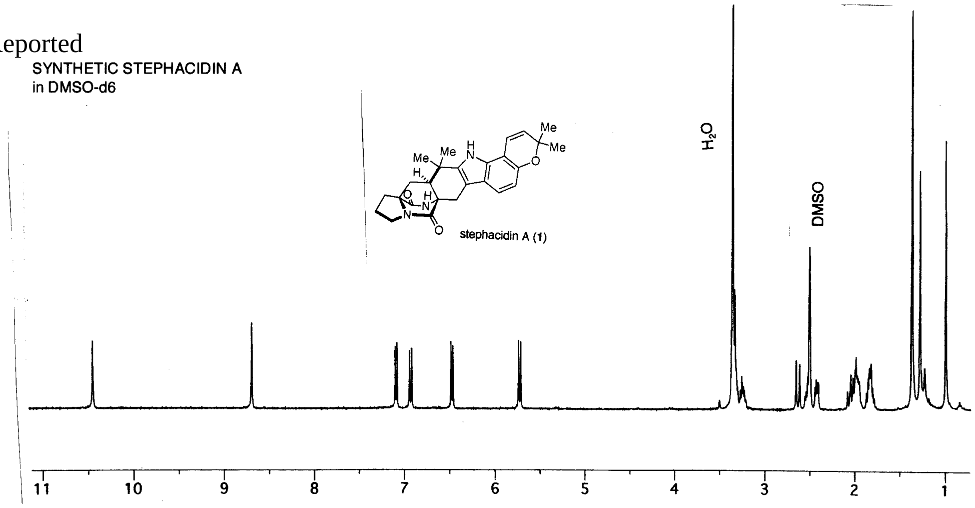

^1H NMR (600 MHz, $(CD_3)_2SO$)

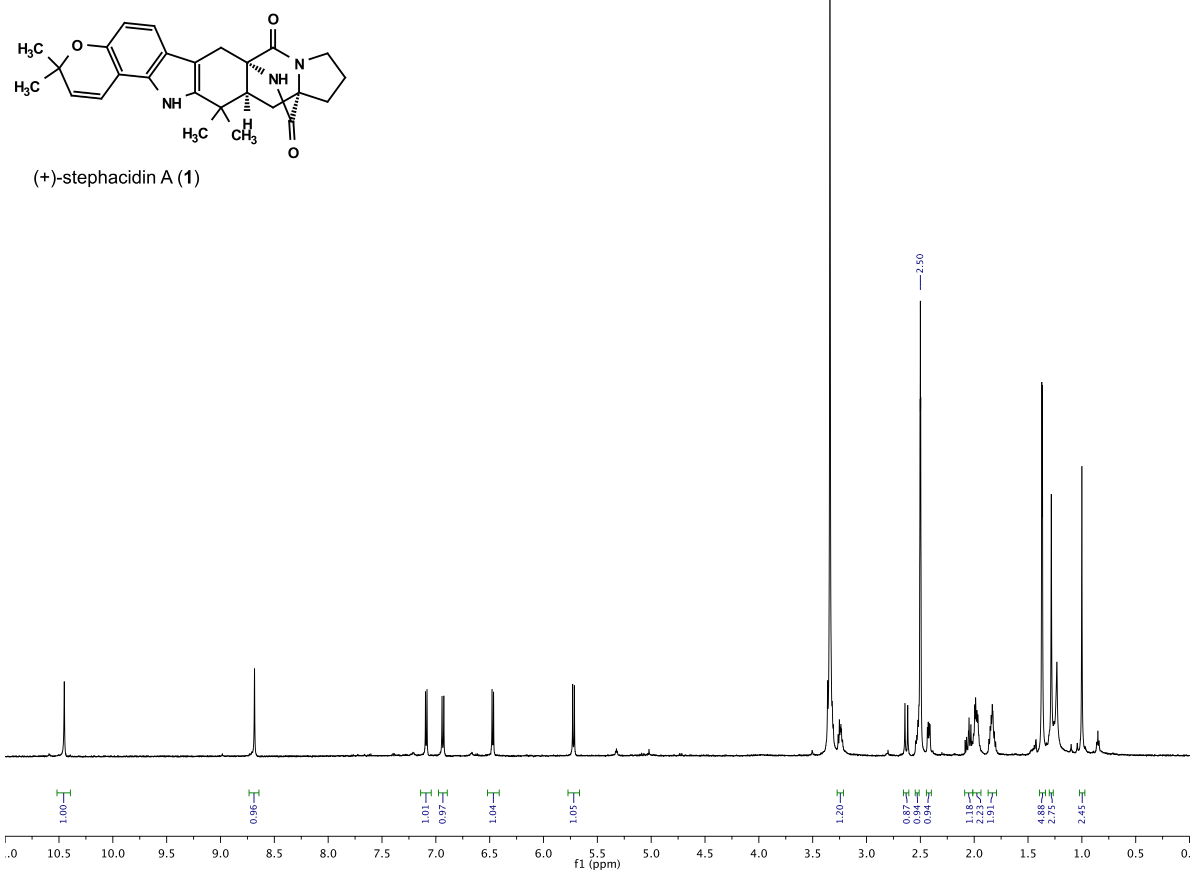

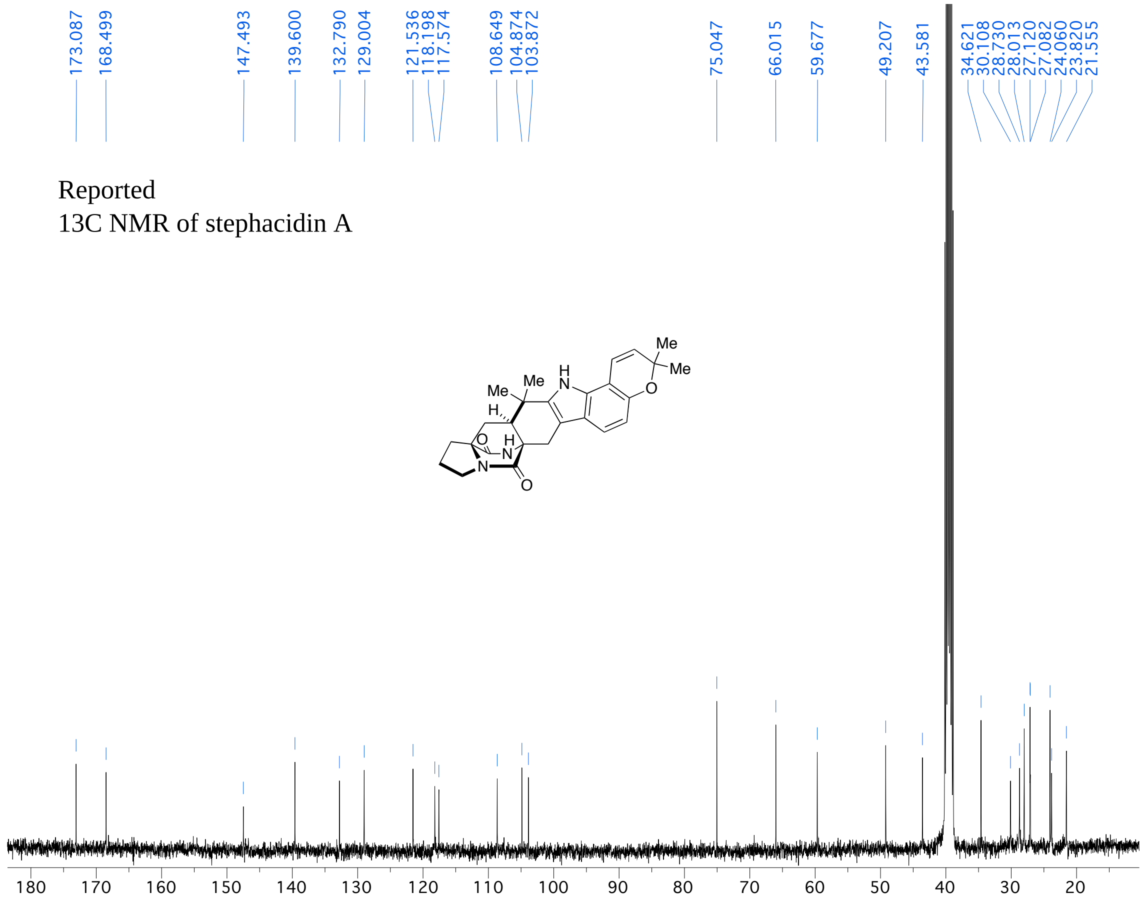

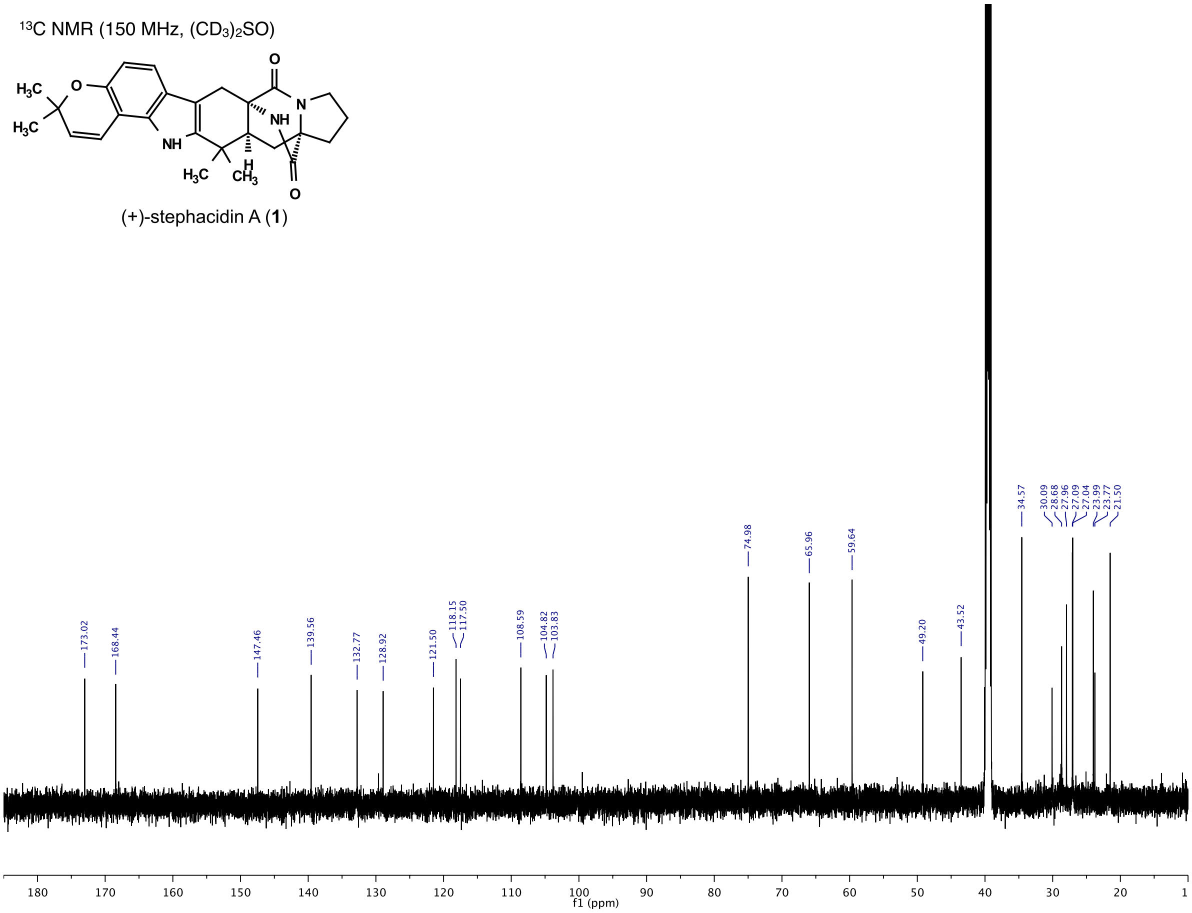

(+)-stephacidin A (**1**)

(a)

Reported
1H NMR Natural (+)-notoamide I

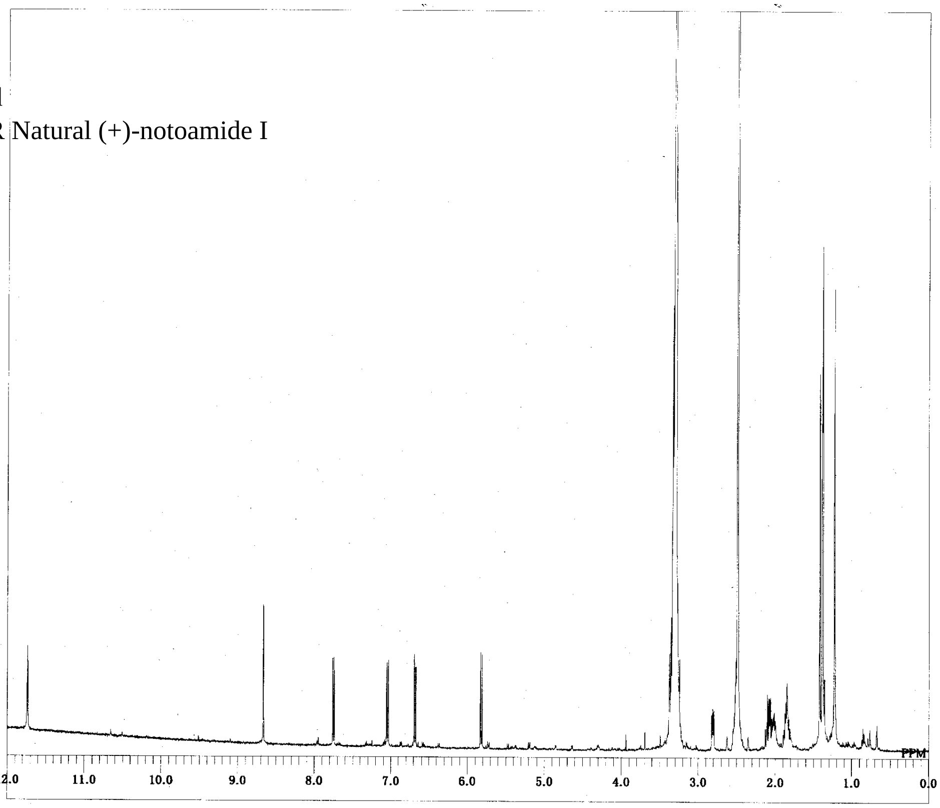

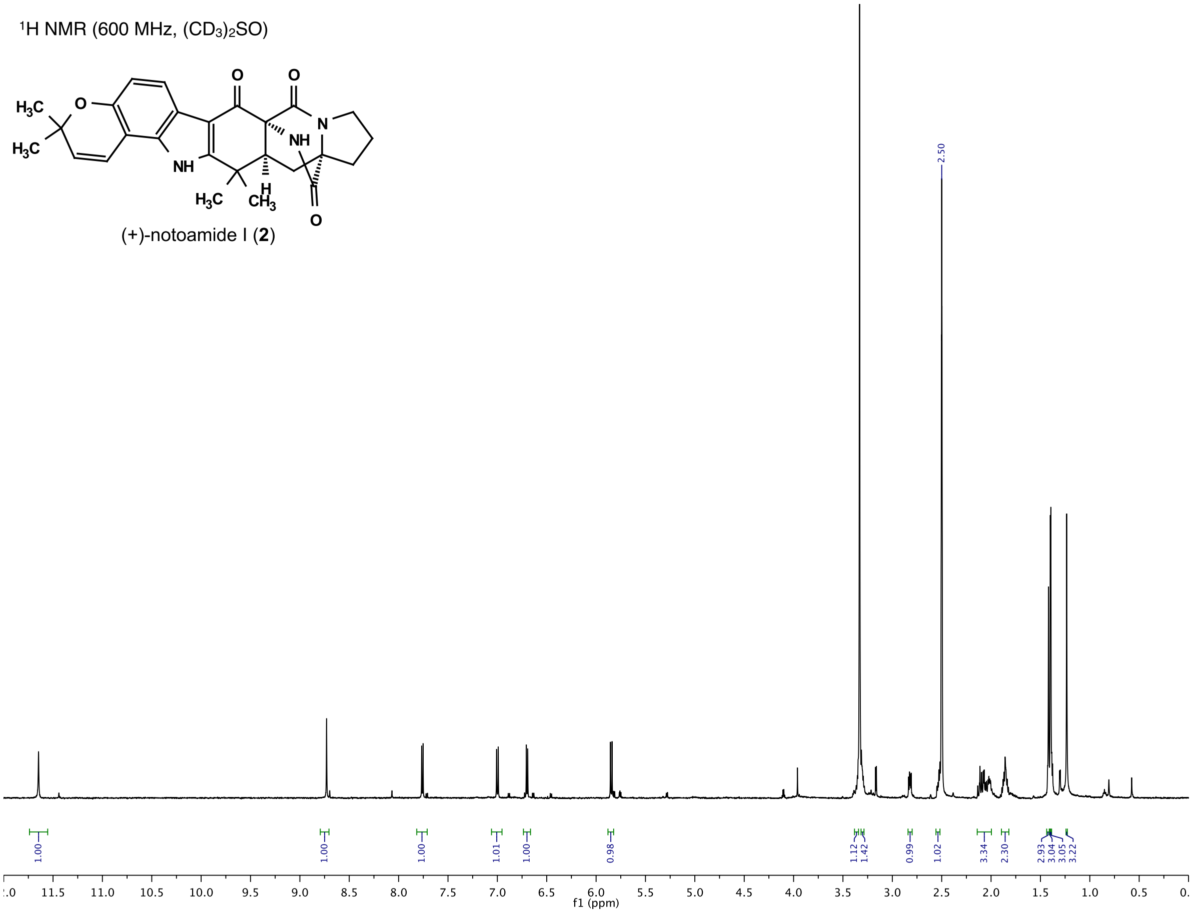

Supplement: Supplementary file 1 [file SC-006-C5SC01977J-s001.pdf]
